# Recent Advances in the Synthesis of Spiroindolines: Catalytic Strategies, Stereoselectivity, and Synthetic Utility (2020–2025)

**DOI:** 10.3390/molecules31142518

**Published:** 2026-07-19

**Authors:** Parthiena M. Keddis, Ahmed Mamdouh Antar, Trevina M. Keddis, Youssef Aboushady, Ashraf H. Abadi, Grigoris Zoidis, Matthias Engel, Mohammad Abdel-Halim, Mennatallah Abdallah

**Affiliations:** 1 Department of Pharmaceutical Chemistry, Faculty of Pharmacy and Biotechnology, German University in Cairo, Cairo 11835, Egypt; parthiena.keddis@guc.edu.eg (P.M.K.); ahmed.ahmed-antar@guc.edu.eg (A.M.A.); trevina.ibrahim@gmail.com (T.M.K.); youssef.aboushady@guc.edu.eg (Y.A.); ashraf.abadi@guc.edu.eg (A.H.A.); mennatallah.abdallah@guc.edu.eg (M.A.); 2 Division of Pharmaceutical Chemistry, Department of Pharmacy, School of Health Sciences, National and Kapodistrian University of Athens, Panepistimiopolis-Zografou, 15771 Athens, Greece; zoidis@pharm.uoa.gr; 3 Pharmaceutical and Medicinal Chemistry, Saarland University, Campus C2.3, D-66123 Saarbrücken, Germany

**Keywords:** spiroindoline, spirocyclization, transition metal catalysis, organocatalysis, photocatalysis, stereoselectivity

## Abstract

The spiroindoline framework is a privileged scaffold in medicinal chemistry, appearing in natural products and in synthetic bioactive compounds, such as BAY 1214784, RO8994, and RK-287107, with reported activities ranging from antimitotic effects to kinase inhibition. This review covers the methods developed between 2020 and 2025 for constructing spiroindoline frameworks, organized first by the site of spirocyclization (C2 versus C3 of the indole) and then by catalyst class: second- and third-row transition metals, first-row transition metals and main-group Lewis acids, organocatalysis, and visible-light photoredox. For each method we discuss the reaction design, the accessible substrate scope, and mechanistic insights, with particular attention to how stereochemistry is controlled. We also highlight representative downstream transformations that demonstrate the synthetic utility of the produced spiroindolines. Progress over the past five years has been substantial, particularly in enantioselective methods that create a single stereocenter and in cascade designs that build complex polycyclic frameworks in a single operation. Asymmetric construction of multiple adjacent stereocenters, gram-scale demonstrations, and genuinely sustainable conditions remain less developed; these areas are priorities for future work.

## 1. Introduction

**Figure 1:**
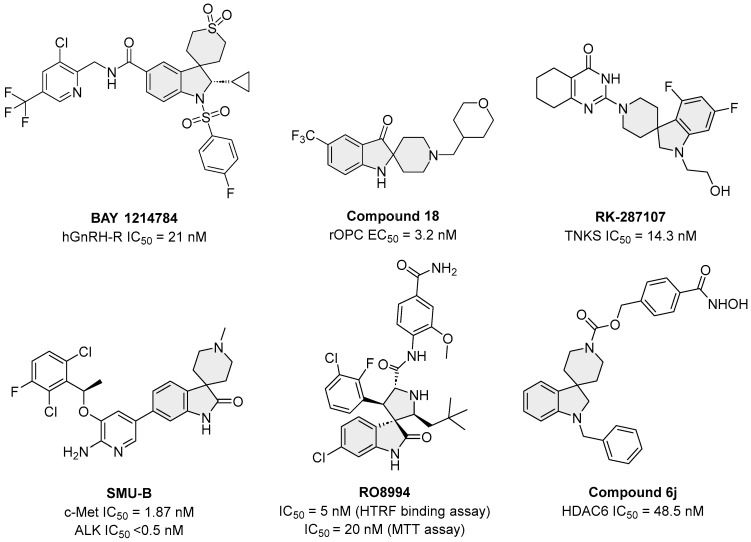
Previously reported potent synthetic spiroindoline-based derivatives.

Spiroindoline scaffolds occupy a special position in modern organic and medicinal chemistry due to their unique three-dimensional architecture and remarkable biological relevance [1,2]. These scaffolds are not only synthetically attractive but are also abundantly represented in nature, being widely distributed across diverse biological sources including plants, fungi, bacteria, actinomycetes, and marine invertebrates [3,4,5,6,7]. Remarkably, the clinically important natural alkaloids Vincristine and Vinblastine contain spiroindoline frameworks and are highly potent anticancer agents that exert their activity by disrupting microtubule assembly and inhibiting cancer cell division [8]. The discovery of spiroindolines in numerous natural alkaloids with complex architectures and potent bioactivities has driven sustained interest in their synthesis and functionalization [6]. Moreover, their structural rigidity combined with conformational adaptability of the spirocyclic core enables efficient interaction with biological targets, further explaining their frequent occurrence in bioactive natural products and drug candidates [9]. Consequently, spiroindolines have emerged as key scaffolds at the interface of natural product chemistry and synthetic methodology development [10]. Among the promising synthetic spiroindoline derivatives is BAY 1214784, identified in a large high-throughput screening (HTS) campaign by Bayer in Panknin et al. as an orally active selective antagonist of the human gonadotropin-releasing hormone receptor (hGnRH-R) with an IC_50_ value of 21 nM, which reached phase I clinical trials showing good tolerability in postmenopausal women (Figure 1) [11]. Katayama et al. developed compound 18, a potent spiroindoline-derived inducer of oligodendrocyte progenitor cell (OPC) differentiation, in a potential disease-modifying strategy for remyelination in multiple sclerosis. Compound 18 showed an EC_50_ of 3.2 nM in an experimental autoimmune encephalomyelitis rat model [12]. RK-287107, an HTS hit reported by Shirai et al., was found to be a spiroindoline-based tankyrase (TNKS1/2) inhibitor with a strong enzymatic potency (TNKS IC_50_ values = 14.3 nM). By blocking tankyrase-driven Wnt/*β*-catenin signaling, it suppressed colorectal cancer cell proliferation [13]. A potent spiroindoline-based dual c-Met/ALK inhibitor, SMU-B, was developed by Li et al. and showed very strong biochemical activity with IC_50_ values of 1.87 nM for c-Met and <0.5 nM for ALK. SMU-B suppresses downstream signaling leading to significant antitumor effects in gastric cancer xenograft models. It acts by blocking receptor tyrosine kinase signaling (c-Met/ALK), thereby inhibiting tumor cell proliferation and survival [14]. RO8994, a spiroindoline-based inhibitor of the p53–MDM2 protein–protein interaction presented in Zhang et al., exhibited a strong binding potency with an IC_50_ of 5 nM (HTRF binding assay) and 20 nM in MTT cell-based assays. By blocking MDM2, it restores p53 activity, leading to apoptosis and tumor growth inhibition in preclinical cancer models, highlighting its promise as a potent anticancer lead [15]. Compound 6j was identified by Saraswati et al. as the most potent spiroindoline-based HDAC6 inhibitor, showing an IC_50_ of 48.5 nM. It acts by selectively inhibiting HDAC6, leading to increased tubulin acetylation and induction of apoptosis in cancer cells, highlighting its potential as an epigenetic anticancer lead [16].

**Figure 2:**
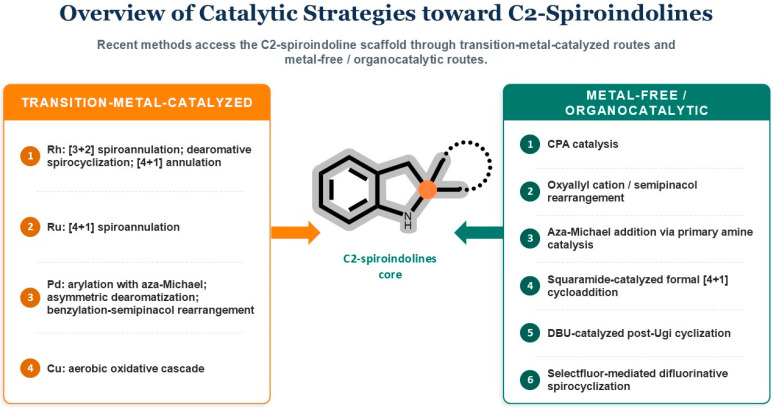
Overview of catalytic strategies toward C2-spiroindoline scaffolds. The methods are grouped into transition metal-catalyzed routes and metal-free/organocatalytic approaches.

Synthetic routes toward spirocyclic indolines have been systematically categorized into three main approaches: interrupted Fischer indolization, dearomatization of the indole nucleus, and condensation-based reactions. Spirocyclization at both the C2 and C3 positions of indole has been accomplished in good-to-excellent yields through the application of diverse inorganic and organic catalytic systems. Asymmetric spirocyclization is well-precedented at the C3 position; however, analogous transformations at C2 were comparatively underexplored, largely owing to the inherently lower reactivity of this position and the scarcity of reliable methodologies for its functionalization. Nevertheless, the past few years have witnessed a surge of interest in this area, with considerable synthetic ingenuity directed toward the development of novel C2-spirocyclic indoline scaffolds, establishing this as an emerging and increasingly important field (Figure 2).

The growing synthetic and pharmacological importance of spiroindoline scaffolds has inspired the publication of several review articles that have shaped this field. Bariwal et al. comprehensively reviewed recent advances in the spirocyclization of indole derivatives, cataloging methodologies for accessing both C3- and C2-spiroindoline frameworks and the diverse inorganic and organic catalytic systems employed, while highlighting the relative underdevelopment of asymmetric C2-spirocyclization strategies [17]. In the domain of cascade reactions, Chen, Lin, and Liu authored an article surveying the construction of polycyclic spiroindolines via 3-(2-isocyanoethyl)indole as a key versatile building block, showcasing how isocyanide-based cascade processes can efficiently forge complex polycyclic spiroindoline architectures in a single synthetic operation [18].

A very recent review by Buttard and Guinchard provided an extensive mechanistic review of spiroindoles as intermediates and products in transition metal-catalyzed dearomatization of indoles, with particular attention to the reactivity and mechanistic evolution of the spiroindoleninium intermediate [19]. Together, these contributions have laid important groundwork; however, they do not comprehensively cover the rapid methodological advances witnessed in the 2020–2025 period. Therefore, an updated review focusing specifically on recent advances during this timeframe is both timely and necessary.

The present review highlights recent advances (2020–2025) in the synthesis of both C2- and C3-spiroindoline derivatives via transition metal-catalyzed as well as metal-free strategies. Particular emphasis is placed on modern methodologies, including cascade and multicomponent reactions, C–H functionalization, and annulation approaches, which enable efficient construction of the spirocyclic framework with high regio- and stereoselectivity.

## 2. Spirocyclization via the 2-Position of the Indole Skeleton

### 2.1. Metal-Catalyzed Methods

#### 2.1.1. Rhodium-Catalyzed Methods

**Figure 3:**
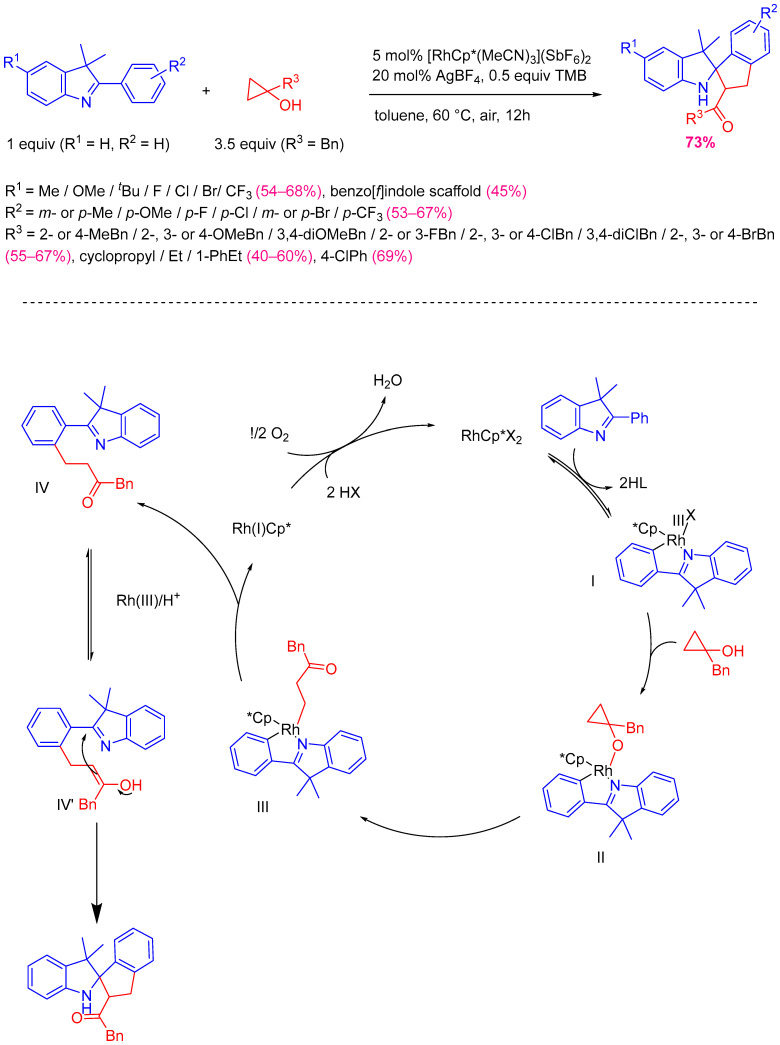
Rhodium(III)-catalyzed C–H activation/annulation of 2-arylindoles.

Yang et al. reported a Rh(III)-catalyzed formal [3 + 2] spiroannulation of 2-aryl-3*H*-indoles with cyclopropanols, in which the indole acts as both directing group and electrophile while the cyclopropanol serves as a masked homoenolate through C-C cleavage (Figure 3; Table S1). With air as the terminal oxidant under mild conditions, C2-spiroindolines were obtained in moderate-to-good yields (40–73%) across diverse indole and cyclopropanol substituents; indoles with C7-substitution, an *ortho*-methoxy aryl, or 2-thienyl, and unsubstituted 2-phenylindole were unreactive, defining the steric/electronic limits of the directing group. However, more complex indole frameworks were compatible, including spirocyclopentane (60%), spirocyclohexane (61%), and 3-ethyl-3-methyl variants (47%, 2:1 diastereomeric ratio (*dr*)). Additionally, the method extends to ibuprofen- (50%) and etodolac-derived (46%) substrates and to gram-scale (46%). Mechanistically, *ortho* C-H metalation forms a rhodacycle that opens the cyclopropanol; reductive elimination with air reoxidation furnishes a *β*-aryl ketone that cyclizes to the spirocycle [20].

**Figure 4:**
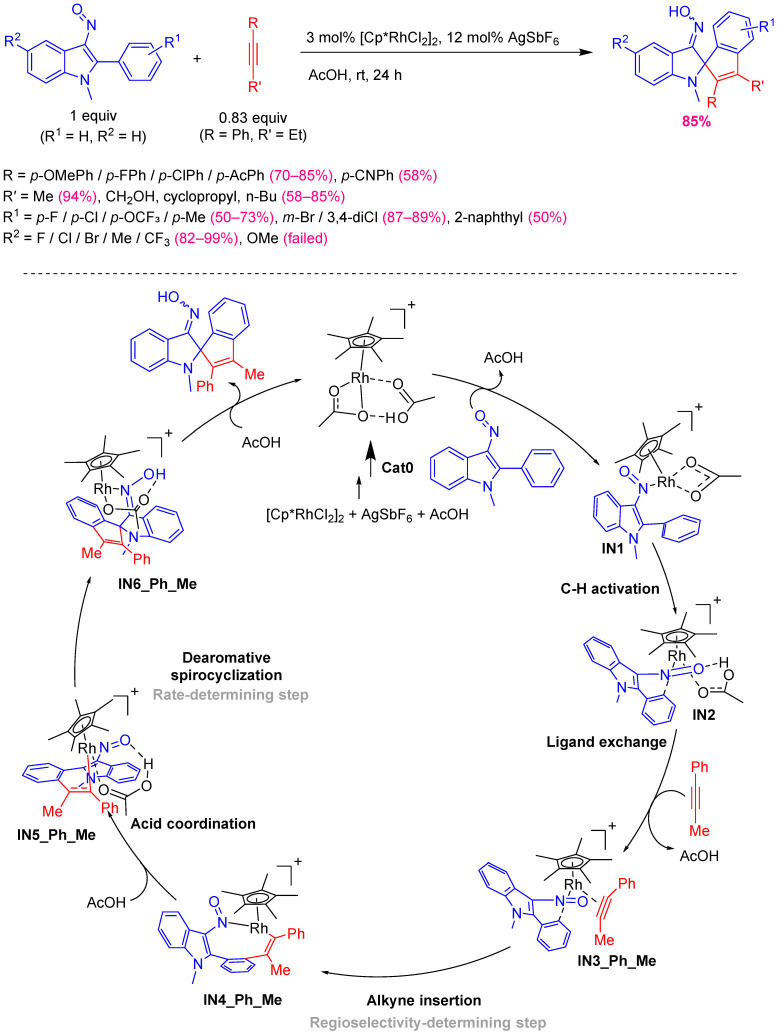
Rhodium(III)-catalyzed C–H activation/annulation of *N*-methyl-2-aryl-3-nitrosoindoles with alkynes.

Li et al. developed a Rh(III)-catalyzed intermolecular dearomative spirocyclization of 2-aryl-3-nitrosoindoles with unsymmetrical alkynes, giving C2-spiroindolin-3-one oximes under mild, redox-neutral conditions with 100% atom economy and complete regiocontrol (>20:1 regioisomer ratios (*rr*)) (Figure 4; Table S2). Yields range 50–99%; the nitroso group both directs C-H activation and is retained as an oxime handle. Ru, Co, and Ir catalysts were inferior. Scope spans alkyl, hydroxy, halo and acyl alkynes, and most indole substituents (F/Cl/Br/Me/CF_3_ 82–99%), while 5-methoxyindole was consistently inert and a cyano-substituted alkyne was less reactive, requiring slightly elevated temperature. A similar substituent tolerance was observed when 1,3-diynes were used in place of aryl alkyl alkynes: both aromatic and aliphatic diynes reacted successfully (47–86%), with 4:1–11:1 *rr*. Substituents including halogens (Cl, Br), trifluoromethoxy, and methyl at *para*- or *meta*-positions of the phenyl rings were tolerated (54–73%), and indole core variations (5-F, 5-Cl, 5-Br, 5-Me) performed well (70–85%), while 5-methoxyindole again failed to provide any detectable product. Density functional theory (DFT) supports nitroso-directed metalation, alkyne insertion, and rate-limiting acid-assisted dearomative spirocyclization, with regioselectivity set during alkyne insertion [21].

**Figure 5:**
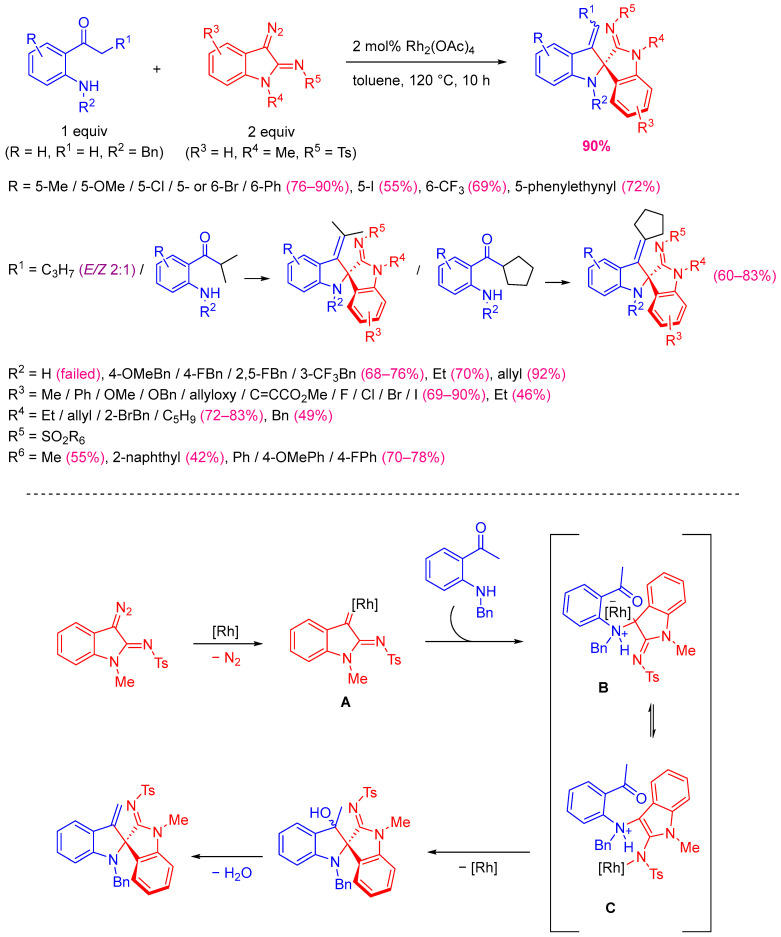
Rhodium-catalyzed [4 + 1]-annulation of easily accessible *o*-acylanilines and 3-diazoindoline-2-imines.

Rupa and Anbarasan reported a Rh_2_(OAc)_4_-catalyzed [4 + 1] annulation of *o*-acylanilines with 3-diazoindoline-2-imines, mainly giving exo-methylene C2-spiroindolines in up to 92% yield (Figure 5; Table S3). A secondary amine on the aniline proved essential (the free amine failed). Both partners tolerated broad substitution such as *N*-alkyl/allyl groups, varied acyl chains, and ring halogens/alkoxy/aryl substituents (generally 46–92%) with sulfonyl variation on the imine giving 42–78%. Additionally, using 7-aza-3-diazoindoline-2-imine exhibited a yield of 64%. The products are versatile, undergoing hydroarylation, LiAlH_4_ imine reduction, triflic-acid 1,2-migration to 2-arylindolines, and NaOH conversion to spirooxindoles. Mechanistically, an *α*-imino Rh carbene is trapped by the *o*-acylaniline to form an ammonium ylide, which cyclizes onto the carbonyl and dehydrates to the spiroindoline [22].

#### 2.1.2. Ruthenium-Catalyzed Methods

**Figure 6:**
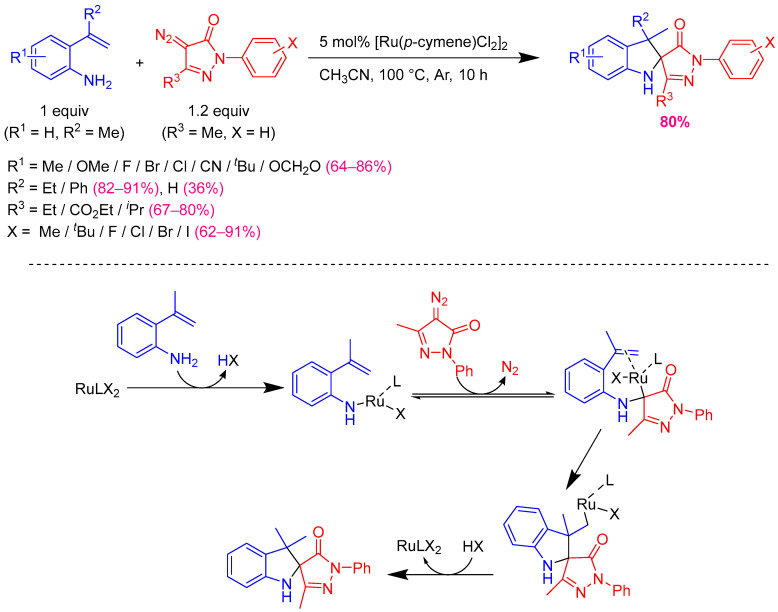
Ru(II)-catalyzed C2-spiroindolines synthesis.

Yu et al. introduced a condition-controlled Ru(II)-catalyzed reaction of alkenylanilines with diazopyrazolones in which atmosphere governs selectivity: aerobic conditions give spirodihydroquinolines through [5 + 1] annulation while argon delivers C2-spiroindolines via [4 + 1] annulation (Figure 6; Table S4). Optimal spiroindoline formation ([Ru(*p*-cymene)Cl_2_]_2_, MeCN, 100 °C, Ar) gave 80% yield, with exclusion of oxygen being essential. Substituents on the aniline aryl (64–86%) and substituted vinyl group (with ethyl/phenyl, 82–91%) were well tolerated. Using 2-(cyclopent-1-en-1-yl)aniline as a substrate afforded the corresponding product in 53% yield, whereas 2-aminostyrene gave only 36%. Replacing the *N*-phenyl of the diazopyrazolone with *N*-naphthyl resulted in the corresponding product in 64% yield. Scale-up and derivatizations underscore synthetic utility. The proposed cycle involves Ru insertion into the N-H bond, vinyl coordination and insertion, and then protonation to release the spiroindoline [23].

#### 2.1.3. Palladium-Catalyzed Methods

**Figure 7:**
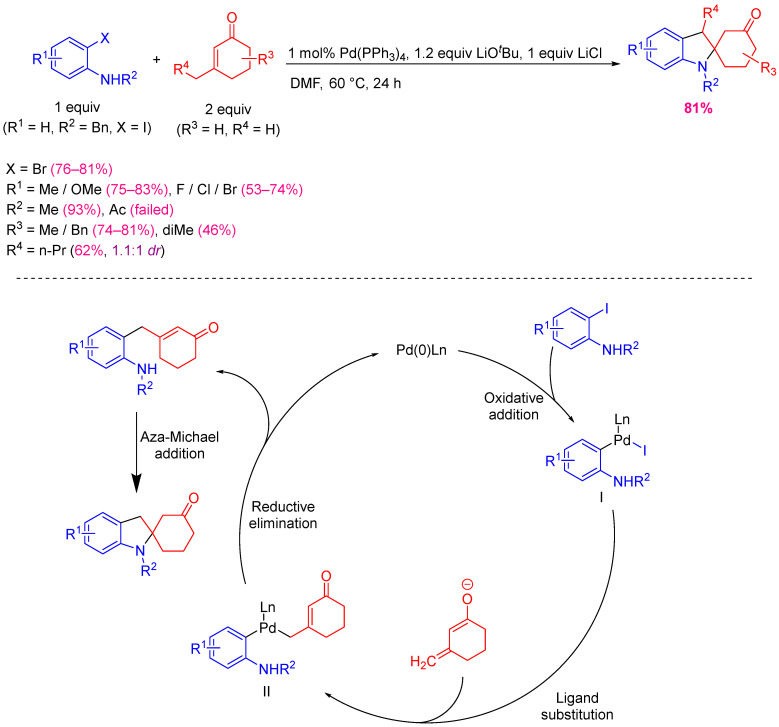
Palladium-catalyzed arylation of carbonyl compounds.

Zhang et al. developed a Pd-catalyzed vinylogous arylation of *β*-substituted cyclic enones with 2-haloanilines, coupled to an intramolecular aza-Michael in a cascade process that assembled complex C2-spiroindolines (Figure 7; Table S5). With Pd(PPh_3_)_4_/LiO*^t^*Bu/LiCl in DMF, the model gave 81%. Electron-donating groups (EDGs) at every position of the 2-iodoanilines gave 75–83%, while halogens gave 53–74% without dehalogenation. (*N*-Me 93%; *N*-Ac inert); 2-bromoanilines required higher catalyst loading/temperature (76–81%). Cyclic enones generally gave good yields and high *dr*, but cyclopentenone (19%) and acyclic enones failed, resulting in a complicated mixture of arylation and homo-condensation products that could not be separated. Additionally, 5,5-dimethyl-2-cyclohexen-1-ones afforded the desired product in only 46% yield along with 42% of the vinylogous arylation product. A related *α*’-arylation/aza-Michael sequence accesses azabicyclo[3.2.2]nonanones. The reaction required different Pd-catalysis derived from Pd(MeCN)_2_Cl_2_ and PPh_3_ and provided moderate-to-good yields. Attempts using chiral phosphine ligands were explored but resulted in only minimal enantiomeric excess (*ee*). The cycle proceeds via oxidative addition, enolate arylation, and base-promoted aza-Michael cyclization [24].

**Figure 8:**
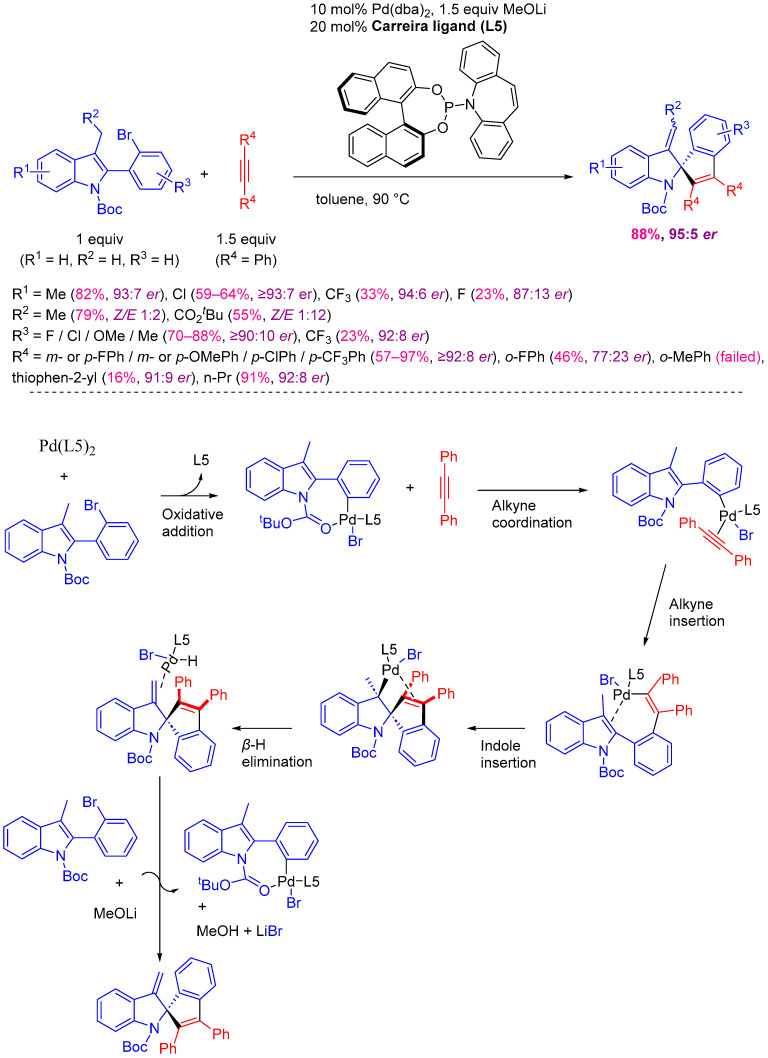
A palladium-catalyzed asymmetric intermolecular dearomatization of C2-arylindoles.

Gao and Jiao reported a Pd-catalyzed asymmetric intermolecular dearomatization of C2-arylindoles with internal alkynes to give C2-quaternary spiroindolines (Figure 8; Table S6). With Pd(dba)_2_/Carreira ligand and MeOLi, the model reached 88% yield and 95:5 enantiomeric ratio (*er*). Arylindoles’ C2-phenyl substitution generally gave 70–88% and >90:10 *er* (4,5-methylenedioxy 99%, 95:5 *er*; CF_3_ lower-yielding 23%, 92:8 *er*; C2-naphthyl 39%, 93:7 *er*). The use of 2-aryl-3-ethylindole substrate gave a mix of geometrical isomers, *Z/E* ratio 1:2, with diminished enantioselectivity and 79% yield. Symmetrical diarylacetylenes performed well with high *er* (except *o*-fluorophenyl 46%, 77:23 *er*; thiophen-2-yl 16%, 91:9 *er*; *o*-methylphenyl failed). Unsymmetrical alkyl/aryl-mixed acetylenes yielded the desired products in decent yields of 78–83% with good enantioselectivities (89:11–94:6 *er*). The Pd(0) cycle—oxidative addition, enantio-determining migratory alkyne insertion, dearomative indole insertion, and *β*-H elimination—delivers the product, which can undergo acid-promoted aza-semipinacol rearrangement to indolenines [25].

**Figure 9:**
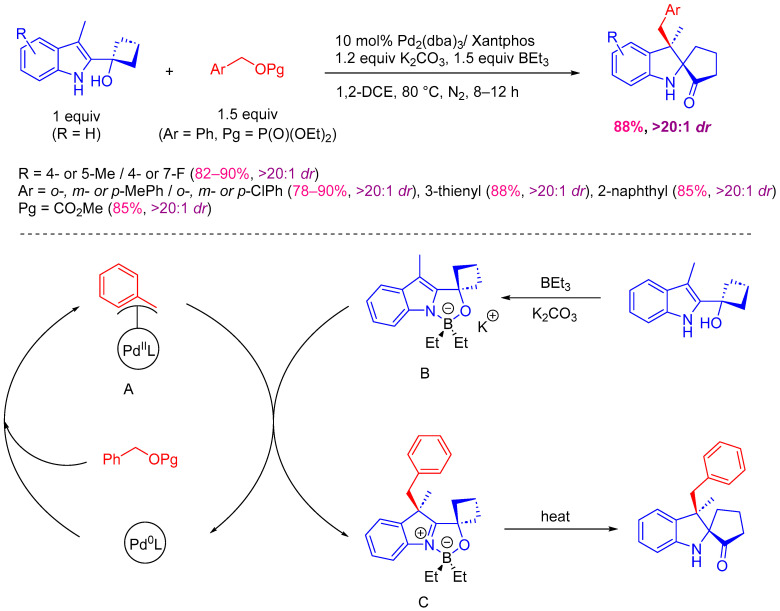
A novel Pd/BEt_3_ synergistic catalytic system enabling a tandem benzylation–semipinacol rearrangement.

Chang et al. devised a Pd/BEt_3_ synergistic system enabling a tandem benzylation-semipinacol rearrangement of 2-cyclobutanol-3-substituted indoles with benzyl phosphates to build C2-spiroindolines bearing a quaternary carbon (Figure 9; Table S7). With Pd_2_(dba)_3_/Xantphos/K_2_CO_3_/BEt_3_ in DCE, yields reached ~88% with >20:1 *dr*. BEt_3_ proved essential for reactivity and selectivity, as were the phosphate-leaving group and Xantphos. Indole ring halogens/alkyl substitution, diverse benzyl phosphates, and 3-thienyl/2-naphthyl instead of the benzyl phosphates’ phenyl all gave 78–90% (>20:1 *dr*). Additionally, a cyclobutanol ring spiro-substrated with cyclohexane produced a complex bis-spiro product (85%, >20:1 *dr*). The reaction efficiently scaled to 2 mmol. Mechanistically, an η^3^-benzylpalladium species couples with the boron-activated indole; strain-driven semipinacol rearrangement then forges the 3-quaternary carbon 2-spiroindolines [26].

#### 2.1.4. Copper-Catalyzed Methods

**Figure 10:**
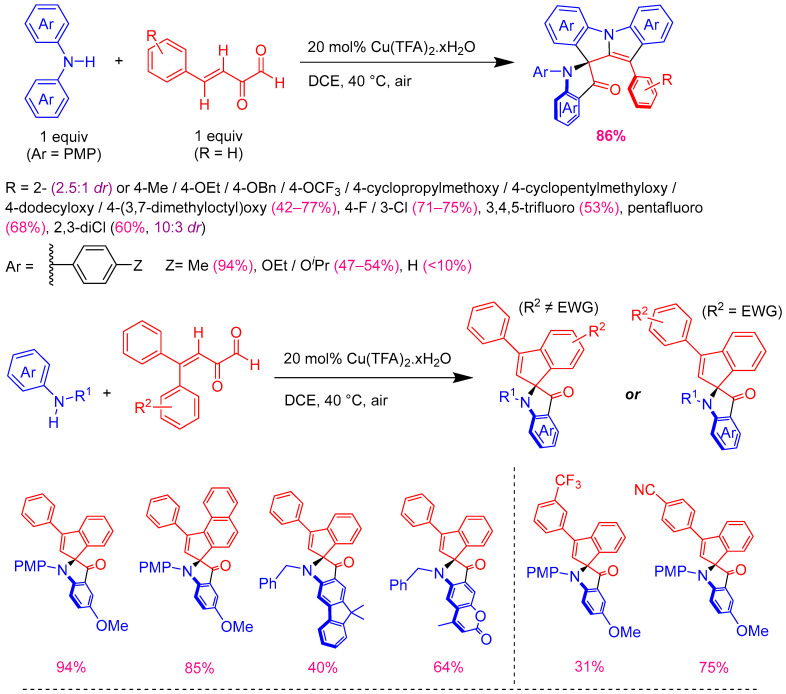

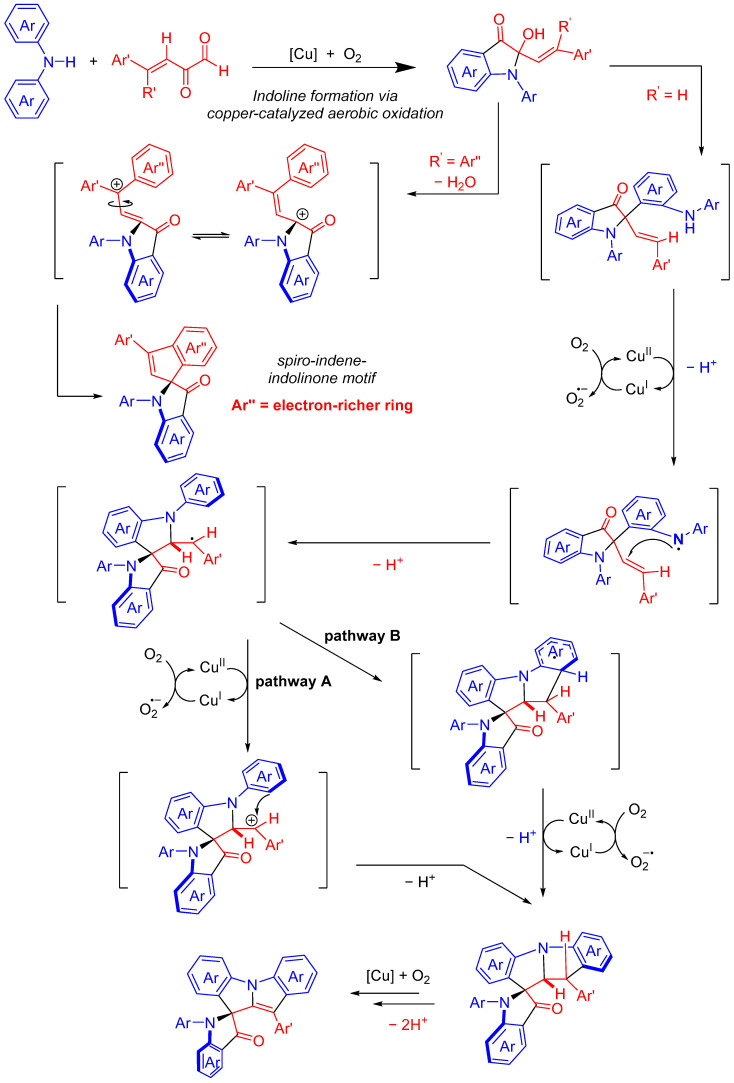
Copper-catalyzed aerobic oxidation.

Lei et al. modified an arylamine/arylglyoxal indolin-3-one synthesis to styrylglyoxals, enabling a Cu-catalyzed aerobic oxidative cascade that builds spiro-fused [6,5,5,6,5,6] hexacycles bearing indolinone, indoline and indole domains (Figure 10; Table S8). With Cu(TFA)_2_ in DCE under air at 40 °C, the model gave 86%. Styrylglyoxals tolerated diverse alkyl/alkoxy/halo and perfluoro groups (electron-withdrawing groups (EWGs) > EDGs), while replacing its phenyl with 1- or 2-naphthyl was successful (82–83%) with 6:1 *dr* for 1-naphthyl. Notably, a mixture of two diastereoisomers was produced upon *ortho* or *meta* substitution of the styrylglyoxals’ aryl. Symmetrical electron-rich diarylamines were required for high efficiency (diphenylamine < 10%). Moreover, the study demonstrated that 1,1-diaryl-substituted styrylglyoxals could yield complex spiro-indene-indolin-3-ones and tolerate EWGs on the aryl ring. The cascade proceeds via a 2-hydroxyindol-3-one intermediate that cyclizes divergently, depending on R’ electronics. Electron-rich aryls at R’ promote direct cyclization, whereas R’ of aryls bearing EWGs favor cyclization through the opposite aryl ring, likely enabled by olefinic *cis*–*trans* isomerization; in contrast, R’ = H results in a completely different pathway involving coupling with a second arylamine [27].

### 2.2. Metal-Free/Organocatalytic Methods (Transition from Metal to Greener/Organocatalytic Approaches)

**Figure 11:**
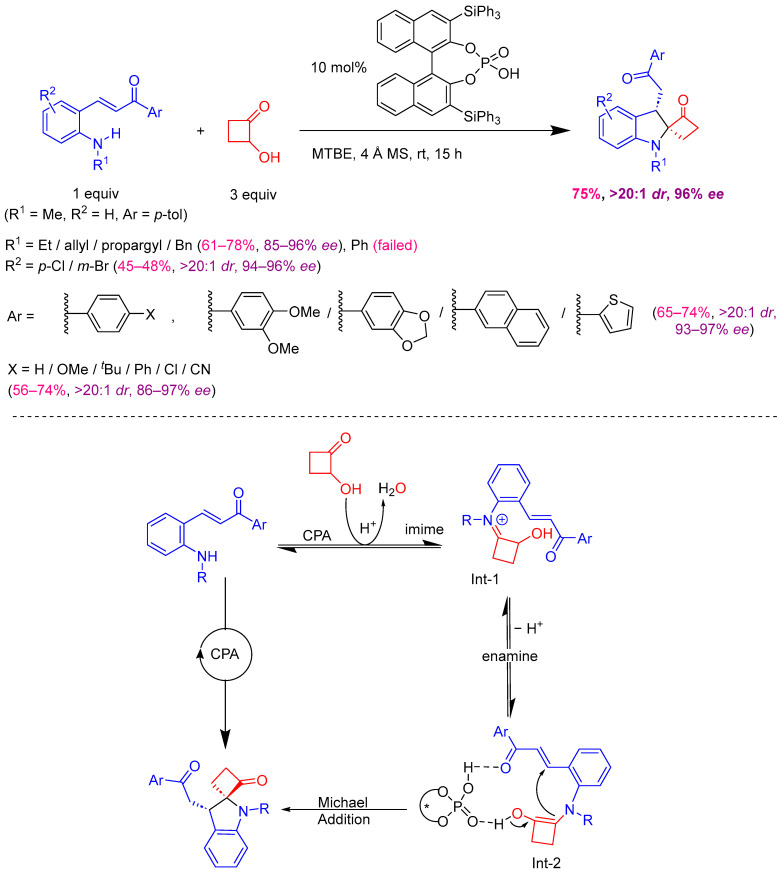
Chiral phosphoric acid (CPA)-catalyzed asymmetric annulation of *N*-substituted 2-arylanilines with 3-hydroxycyclobutanone. The asterisk (*) indicates the remaining BINOL-derived framework of the CPA catalyst depicted in the reaction scheme.

Panda and Ghorai reported the first enantioselective synthesis of cyclobutanone-containing spiroindolines and spiropyrrolidines via a chiral phosphoric acid (CPA) based on modified Heyns rearrangement (Figure 11; Table S9). With a TiPSY-containing CPA catalyst and 4 Å MS in MTBE, aminochalcones and *α*-hydroxycyclobutanone gave spiroindolines in 75% yield, >20:1 *dr*, 96% *ee*. Non-polar ethereal solvents (65–89% *ee*) and 4 Å MS were optimal; Brønsted bases and thiourea/squaramide catalysts failed. The substrate scope was generally broad with high *dr* (>20:1); the *N*-phenyl substrate failed, and bulky *N*-propargyl and *N*-benzyl needed higher loading (20 mol%) and longer times (90 h). Mechanism includes amine condensation to an iminium, enamine isomerization, then enantio-determining Michael addition/spirocyclization [28].

**Figure 12:**
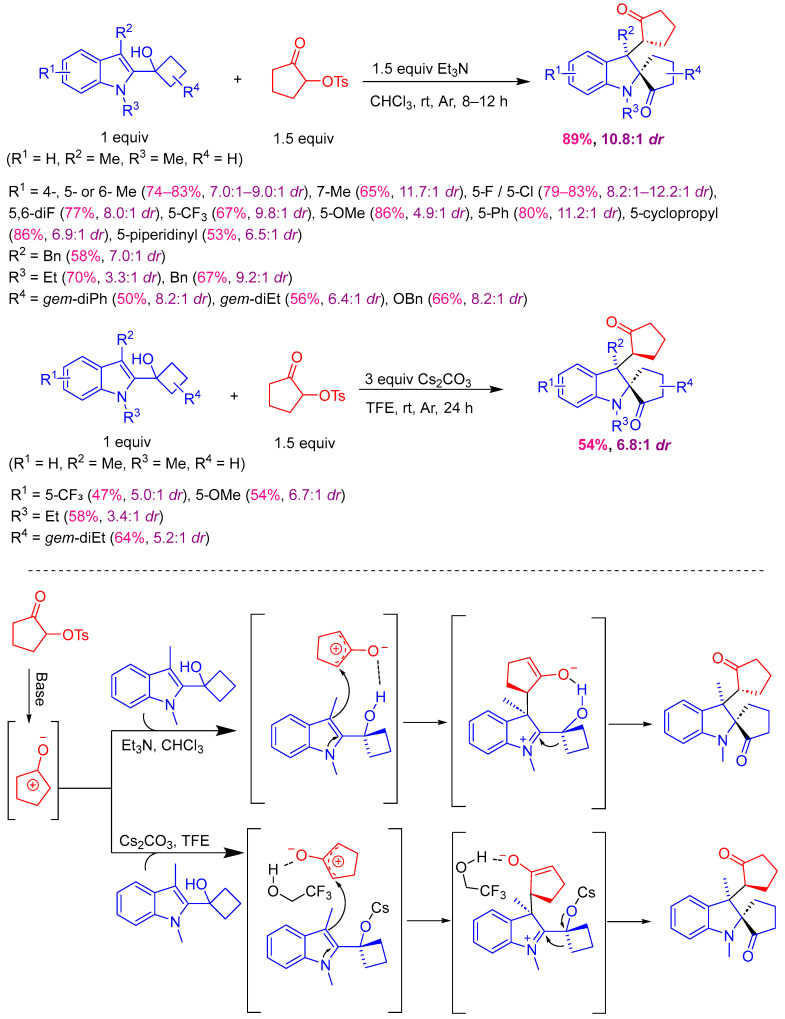
Oxyallyl-cation-promoted semipinacol rearrangement.

Xie et al. reported an oxyallyl-cation-promoted semipinacol rearrangement of indolyl allylic alcohols with 2-tosyloxyketones for stereodivergent C2-spiroindolines bearing vicinal quaternary stereocenters (Figure 12; Table S10). With Et_3_N in CHCl_3_, the model gave 89% (10.8:1 *dr*); base/solvent choice tuned both yield and *dr*. 2-Tosyloxycyclohexanone, 2-bromocyclopentan-1-one and 2-chlorocyclopentan-1-one, as oxyallyl cation sources only gave trace amounts. The free allylic OH was essential (trimethylsilyl (TMS) protection suppressed reactivity). Indole-ring and *N*/C3 substituents were broadly tolerated, with sterics and electronics modulating *dr*. Among cyclobutanol moieties (R^4^), the substrate with oxy-cyclobutanol afforded the corresponding product in 78% yield with the best diastereoselectivity (13.0:1 *dr*). Switching to Cs_2_CO_3_/trifluoroethanol (TFE) inverts selectivity to the complementary diastereomer. Mechanistically, a base-generated oxyallyl cation engages the indole in a dearomative 1,2-migration, with H-bonding possibly controlling diastereodivergence [29].

**Figure 13:**
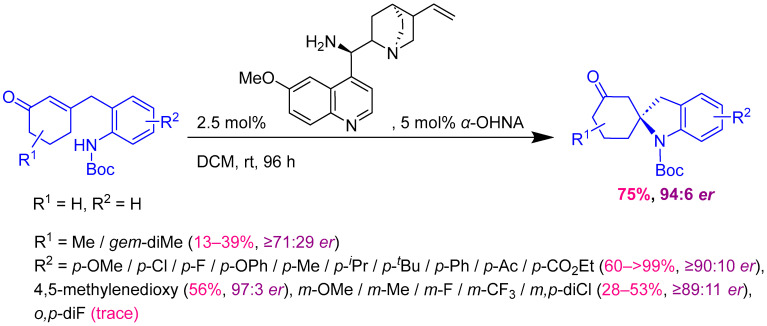
Organocatalytic aza-Michael addition reaction.

Sah et al. developed an organocatalytic intramolecular aza-Michael addition giving quaternary-stereocenter C2-spiroindolines, using a primary quinine-amine catalyst with *α*-hydroxynaphthoic acid (*α*-OHNA) as additive (Figure 13; Table S11). One-pot synthesis of the spiroindoline was unsuccessful, thus a *γ*-arylated enone precursor was first prepared from silyl dienol ether with a Boc-protected 2-bromo-*N*-phenylaniline, then cyclized (DCM, 25 °C) to the spiroindoline with good enantioselectivity. The additive and DCM were critical; other acids/solvents mostly eroded *ee*. *Para*- and *meta*-substituted aniline rings gave good conversion and *er* (>89:11), whereas enone substitution lowered yield/*ee*. A pyridyl variant, instead of aryl, abolished enantiocontrol (45%, 46:54 *er*) possibly due to interference from the additional coordinating nitrogen. The reaction was not successful using a verbenone-derived substrate, as it resulted in a complex mixture. Partial reduction in the spiroindolines’ ketone was achieved and subsequent *N*-Boc deprotection required FeCl_3_ to avoid racemization. Mechanism involved iminium activation stabilized by *α*-OHNA, with facially selective 1,4-aza-Michael addition. Additional stabilization by *α*-OHNA through hydrogen bonding and π-stacking interactions is proposed to contribute to the observed enantioselectivity [30].

**Figure 14:**
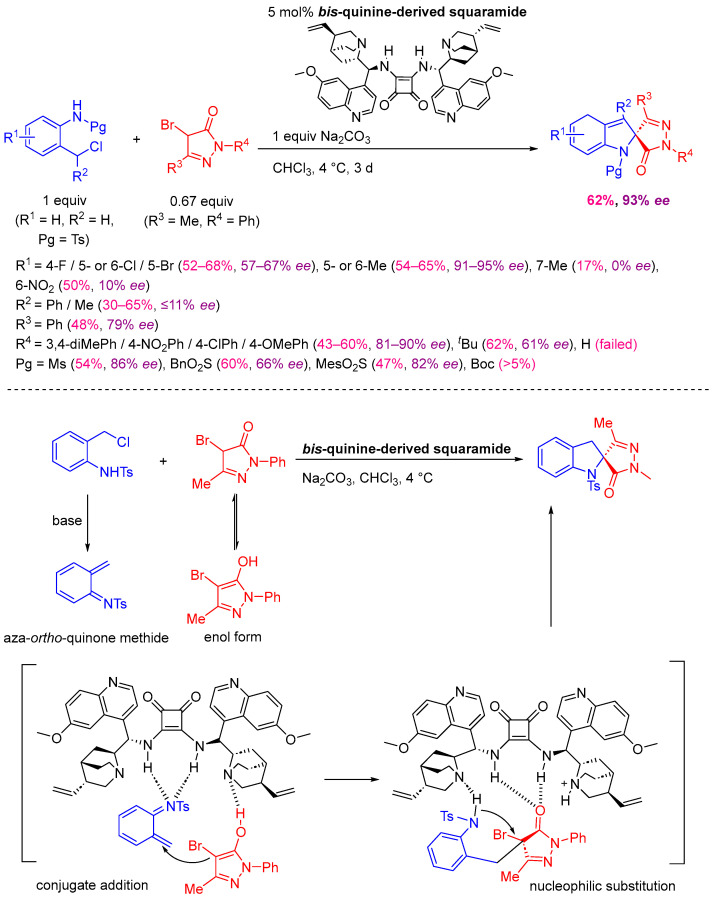
Synthesis of spiroindoline–pyrazolone derivatives.

Carceller-Ferrer et al. reported an organocatalytic formal [4 + 1] cycloaddition between 4-bromopyrazolones and in situ aza-*ortho*-quinone methides (QM), giving chiral C2-spiroindoline-pyrazolones (Figure 14; Table S12). A bis-quinine squaramide with Na_2_CO_3_ in CHCl_3_ at 4 °C gave 62% and 93% *ee*. *N*-Mesyl protection on the aza-*o*-QM precursor was tolerated; halogen substituents on the aryl gave good yields but moderate *ee*, while methyl substitution gave >90% *ee*. *N*-aryl bromopyrazolones (NO_2_/Cl/Me/OMe) gave 43–60%, 81–90% *ee*, whereas N-H variants failed. Mechanistically, the base generates the aza-*o*-QM while the squaramide H-bond activates the enolized pyrazolone for conjugate addition and intramolecular nucleophilic substitution of bromide. A control experiment using benzyl chloride as electrophile resulted in no reaction, supporting the involvement of aza-*o*-QM as the key electrophilic intermediate [31].

**Figure 15:**
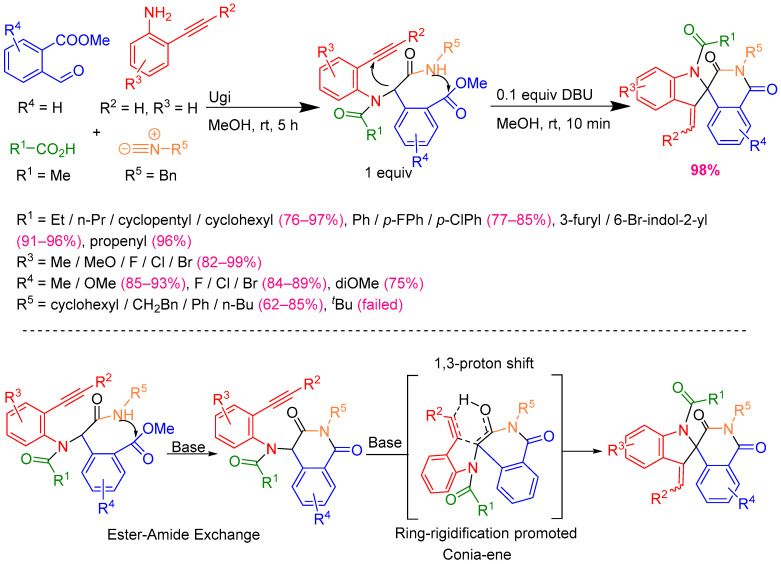
The synthesis of bioactive benzo-fused spiroindolines using DBU catalysis.

Zhao et al. developed a DBU-catalyzed post-Ugi amide–ester exchange/Conia-ene double cyclization giving benzo-fused C2-spiroindolines under mild, metal-free conditions (Figure 15; Table S13). Identifying a cyclic imide intermediate that rigidifies the system allowed Conia-ene cyclization at room temperature, avoiding the usual harsh thermal or Au/Ag conditions; the model reached 98% in MeOH in 10 min. All four Ugi components tolerated broad variation (generally 62–99%); *tert*-butyl isocyanide failed likely due to steric hindrance, and cyclohexyl isocyanide required 1.1 equiv DBU. Green credentials were demonstrated by performing the reaction in aqueous sodium bicarbonate and refluxing water (52–53%, 84% under microwave). Mechanism includes DBU-promoted amide–ester exchange to a cyclic imide, enolization, then 5-exo-dig cyclization [32].

**Figure 16:**
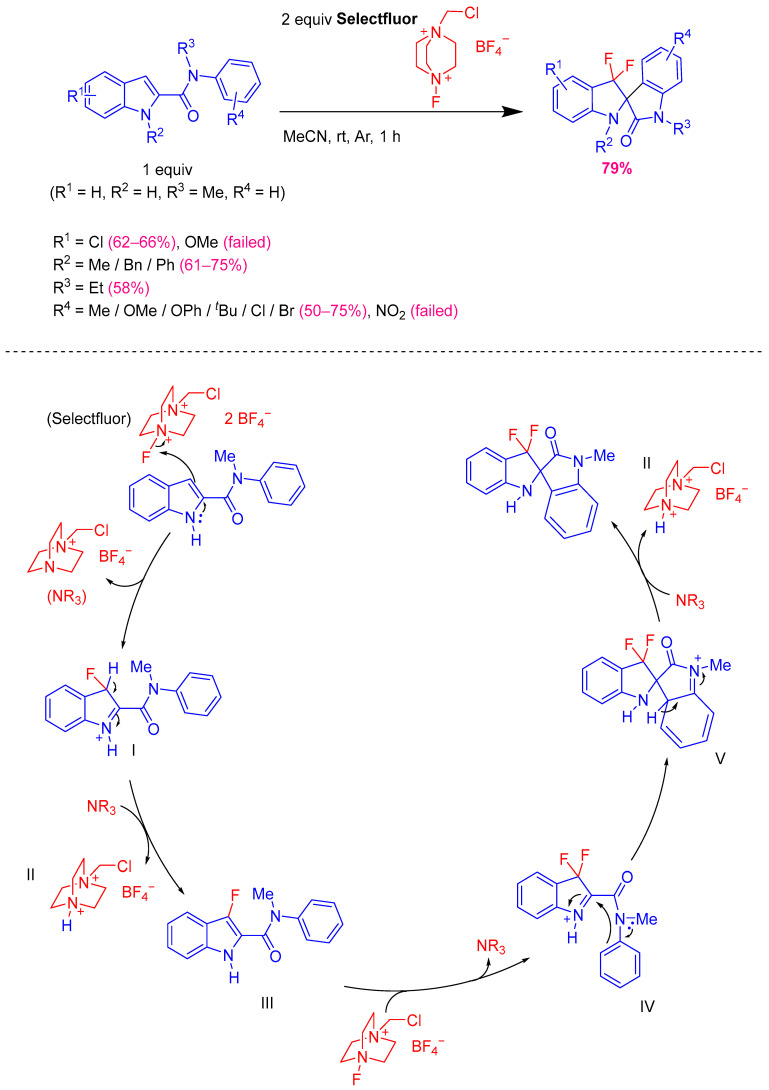
A catalyst-free *gem*-difluorination.

Dong et al. reported a catalyst-free *gem*-difluorination/spirocyclization of indole-2-carboxamides giving *gem*-difluorinated C2-spiroindolines (Figure 16; Table S14). Selectfluor acts dually as the electrophilic F source and, via its tertiary-amine byproduct, as an in situ base, removing the need for added catalyst/base; 2 equiv in dry MeCN (rt, 1 h, Ar) gave 79% (73% in air). Polar aprotic solvents were essential. EWGs on the indole were tolerated (62–66%) while a strong EDG gave a messy reaction. *N*-H at R^3^ failed, suggesting that the weak electron-donating nature of the *N*-alkyl group is necessary and strong *para*-NO_2_ at R^4^ shut down spirocyclization due to reduced electron density. Upon utilizing tetrahydroquinoline derivatives and *N*-methylnaphthalenamine, the desired products were afforded in 42–66% yields. Promotion of BF_3_ enabled the hydrolysis of C2-spiroindoline adducts to C2-spirocyclic oxindoles in good to excellent yields. Mechanism comprises indole attack on Selectfluor, amine-mediated aromatization to a fluoroindole, further fluorination and intramolecular nucleophilic addition/aromatization [33].

An overview of the catalytic approaches developed for C2-spiroindoline synthesis is provided in Table 1, including the key reaction conditions, substrate combinations, maximum reported yields, and selectivity outcomes.

## 3. Spirocyclization via the 3-Position of the Indole Skeleton

**Figure 17:**
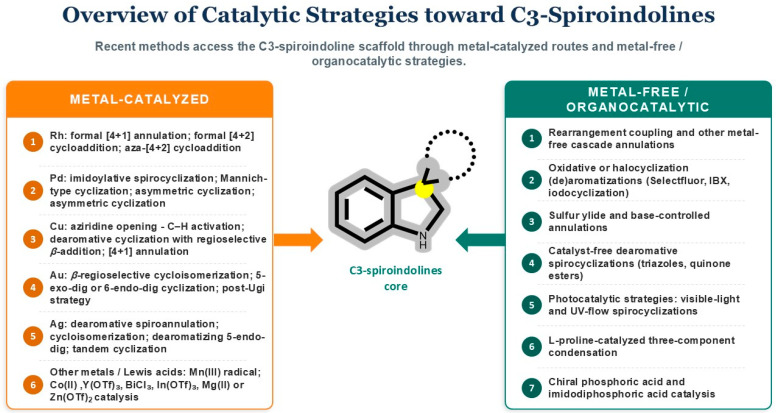
Overview of catalytic strategies toward C3-spiroindoline scaffolds. The methods are grouped into metal-catalyzed routes and metal-free/organocatalytic approaches.

Diverse C3-spiroindolines were developed (Figure 17). Metal-catalyzed methods involving rhodium, palladium, copper, gold, silver and mixed metals are hereby discussed as well as metal-free and organocatalytic methods.

### 3.1. Metal-Catalyzed Methods

#### 3.1.1. Rhodium-Catalyzed Methods

**Figure 18:**
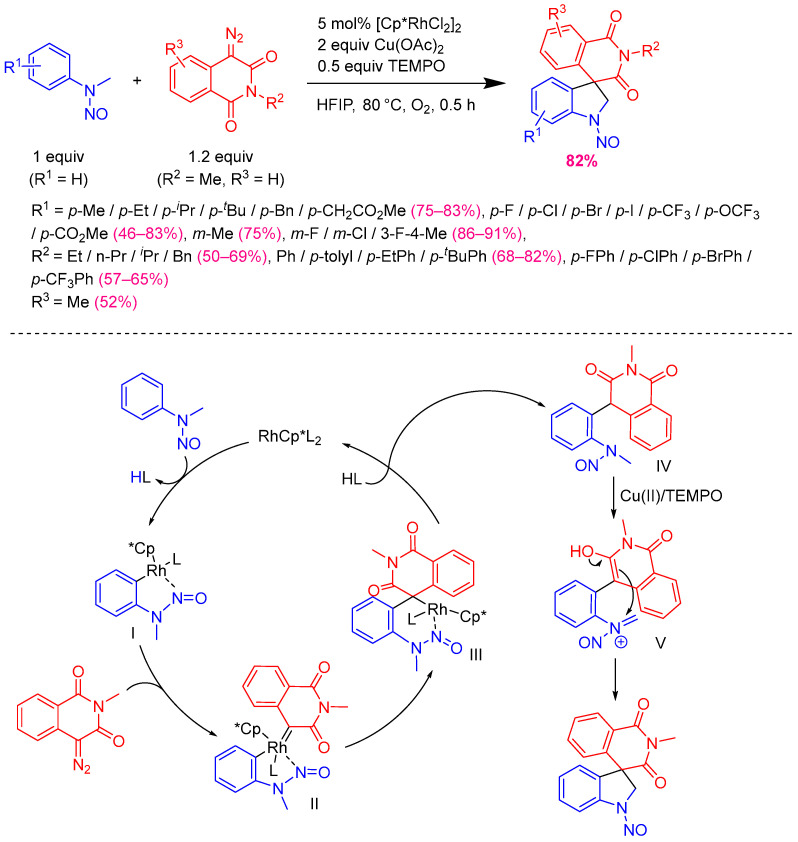
Rh(III)-catalyzed C(sp^2^)–H/C(sp^3^)–H bond functionalization of *N*-methyl-*N*-nitrosoanilines.

Wang et al. disclosed a Rh(III)-catalyzed C(sp^2^)-H/C(sp^3^)-H cascade of *N*-methyl-*N*-nitrosoanilines with diazo homophthalimides in which the *N*-methyl unit is engaged as part of a C_3_N_1_ synthon for formal [4 + 1] spiroannulation (Figure 18; Table S15). With [Cp*RhCl_2_]_2_/Cu(OAc)_2_/TEMPO in hexafluoroisopropanol (HFIP) under O_2_, the model reached 82%. Electron-rich *para*-anilines outperformed electron-poor ones; critically, only *N*-methyl anilines reacted. A *m*-methyl group led to functionalization at the less hindered site (75%), contrary to *m*-halo which opted for the more congested *ortho*-position, attributed to an auxiliary directing effect of the electronegative halogens. Naphthalene- and dibenzo[*b*,*d*]thiophene-derived nitrosoanilines are competent partners (42–78%), and thymol- and menthol-based frameworks gave hybrid spiroindolines (51–68%). *N*-alkyl/aryl diazo homophthalimides gave 50–82%, while diazopyrazolones only depicted 27–42% yield and classical diazo carbonyls failed. Denitrosation of the model spiroindoline with SnCl_2_·2H_2_O (75%) was performed without reduction in the embedded carbonyl group. Kinetic isotope effect (KIE), H/D-exchange and a competent isolated rhodacycle support reversible *ortho*-metalation, Rh-carbene migratory insertion, then Cu/TEMPO benzylic oxidation to an iminium-trapped intramolecularly [34].

**Figure 19:**
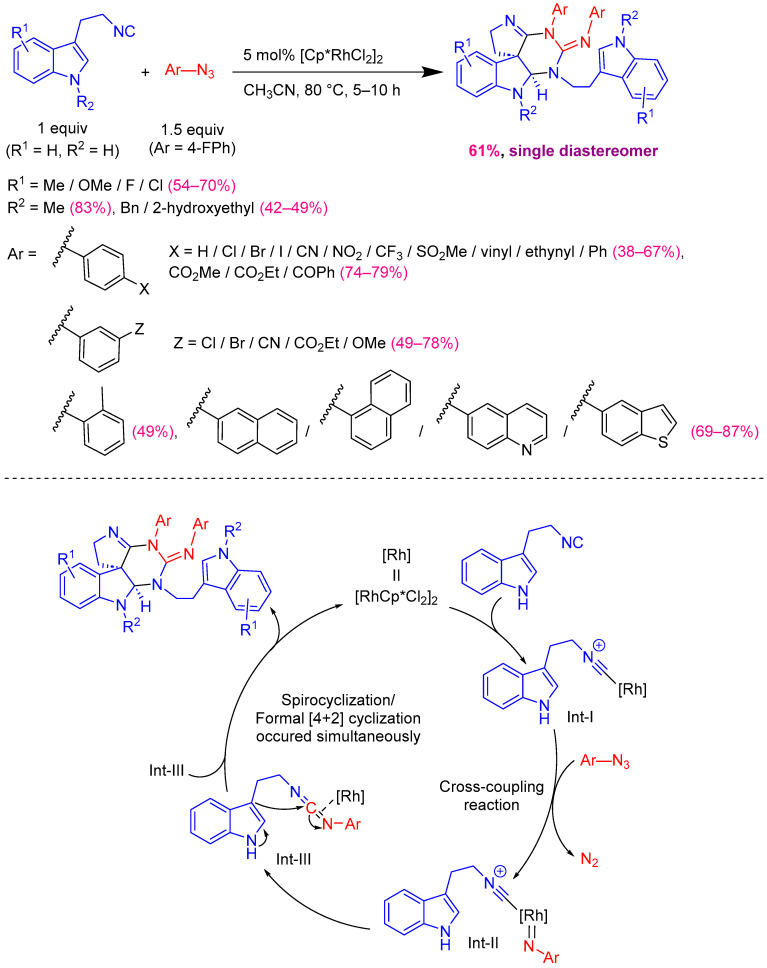
A novel rhodium-catalyzed spirocyclization and formal [4 + 2] cycloaddition.

Gu et al. developed a Rh-catalyzed cascade merging cross-coupling, spirocyclization and formal [4 + 2] cycloaddition that builds polycyclic spiroindolines bearing a pentasubstituted guanidine from 3-(2-isocyanoethyl)indoles and aryl azides (Figure 19; Table S16). With [Cp*RhCl_2_]_2_ in MeCN at 80 °C, the model gave 61% as a single diastereomer. Aromatic azides with diverse EDG/EWG and even alkenyl/alkynyl groups worked (38–87%), while alkyl azides failed, underscoring the need for an aryl group. C2-substituted indoles were inhibited by sterics; varied *N*-groups gave 42–83% and using a benzo[*g*]-indole derivative achieved 42%. Resulting spiroindolines undergo efficient aza-Michael addition at the indole nitrogen, exclusively producing *E*-isomers. Mechanism involves Rh–isocyanide coordination, nitrene formation from the azide, migratory insertion to a carbodiimide, and then concerted spirocyclization/[4 + 2] [35].

**Figure 20:**
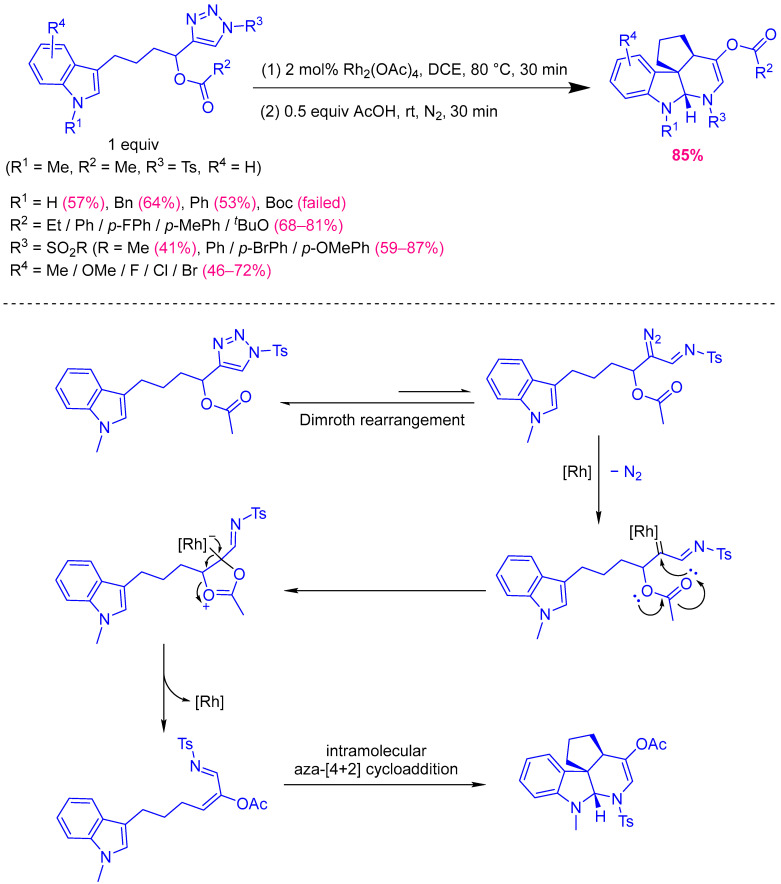
A novel rhodium-catalyzed cascade strategy that combines 1,2-acyloxy migration with intramolecular aza-[4 + 2] cycloaddition.

Chen et al. reported a Rh_2_(OAc)_4_-catalyzed cascade combining 1,2-acyloxy migration with intramolecular aza-[4 + 2] cycloaddition of ester-tethered 1,2,3-triazoles, assembling [6,5,5,6] and [6,5,6,6] polycyclic spiroindolines under mild conditions (Figure 20; Table S17). Acidic additive (AcOH) was crucial. *N*-EDG indoles were superior to *N*-EWGs (low-yielding or unreactive); various acyloxy migrating groups, including Boc, gave 68–81%, and arylsulfonyl outperformed alkylsulfonyl substituents. Mechanism comprises Dimroth rearrangement and N_2_ loss, which give a Rh carbenoid that undergoes a 1,2-acyloxy shift; then the derived aza-diene engages the indole in an intramolecular aza-[4 + 2] [36].

#### 3.1.2. Palladium-Catalyzed Methods

**Figure 21:**
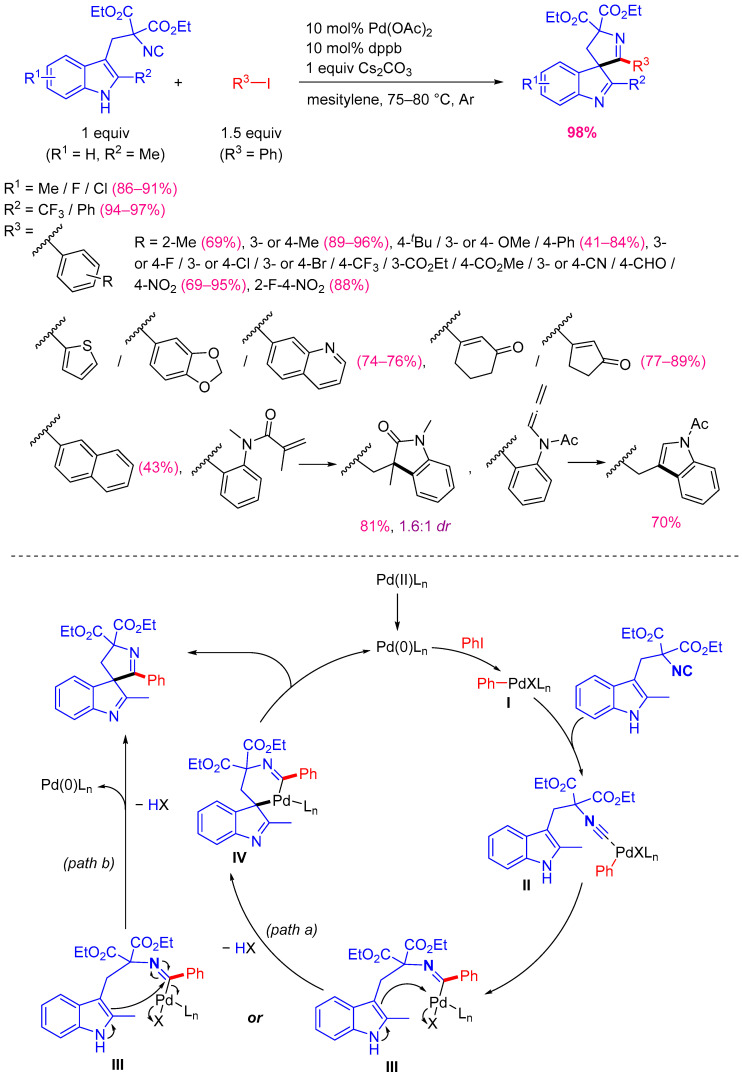
Palladium-catalyzed synthesis of 2′-aryl and vinyl substituted spiroindoline derivatives.

Tang et al. reported a Pd-catalyzed imidoylative spirocyclization of 3-(2-isocyanoethyl)indoles with aryl/vinyl halides giving 2′-aryl/vinyl C3-spiroindolines (Figure 21; Table S18). With Pd(OAc)_2_/dppb/Cs_2_CO_3_ in mesitylene (dropwise addition of indoles essential, within 1.5 h), the model reached 98%. Aryl iodides bearing EDGs/EWGs and heteroaryls were tolerated, EWGs favored, and diverse isocyanides performed well. Alkenyl iodides and menthol- (78%) and estrone-derived (75%) aryl iodides also generated good yields. 2-Methyltryptophan-derived isocyanide gave 97% and 1:1 *dr*; tryptamine-derived isocyanide failed. Dearomative spirocyclization was also successful with aryl isocyanides (93%). The platform also delivers oxindole-spiroindoline, an indole-spiroindoline derivative by allene-insertion, and double-dearomatization polyindoline scaffolds. SPINOL-phosphoramidite ligand enabled an asymmetric variant (86%, 85% *ee*), whereas F and OMe substitution on the indole also produced acceptable yields with high *ee*. Mechanism includes oxidative addition, isocyanide insertion to an imidoyl-Pd, then dearomative indole attack/reductive elimination [37].

**Figure 22:**
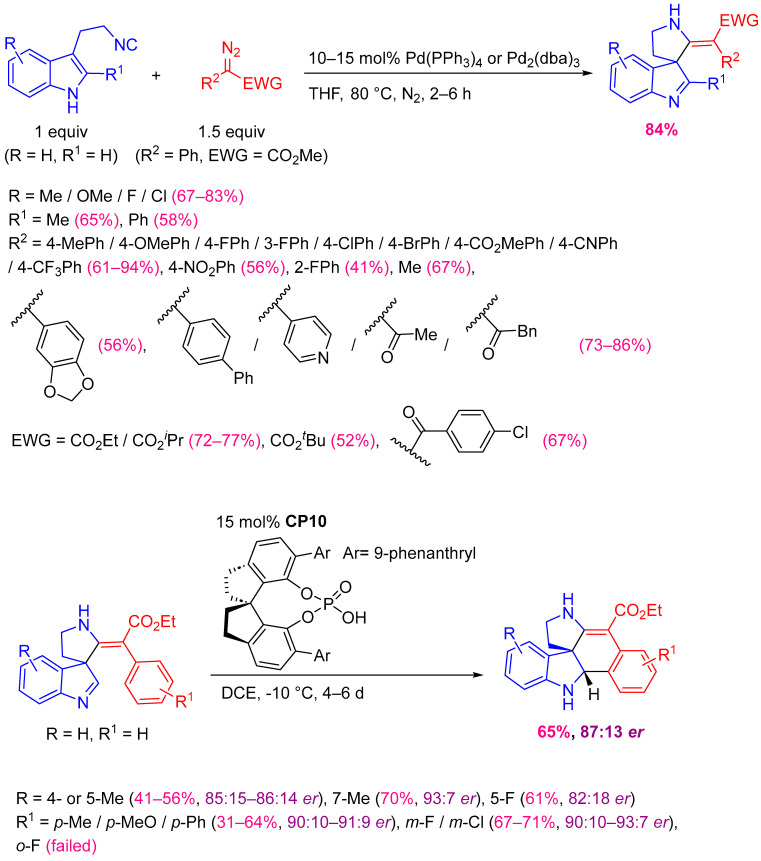

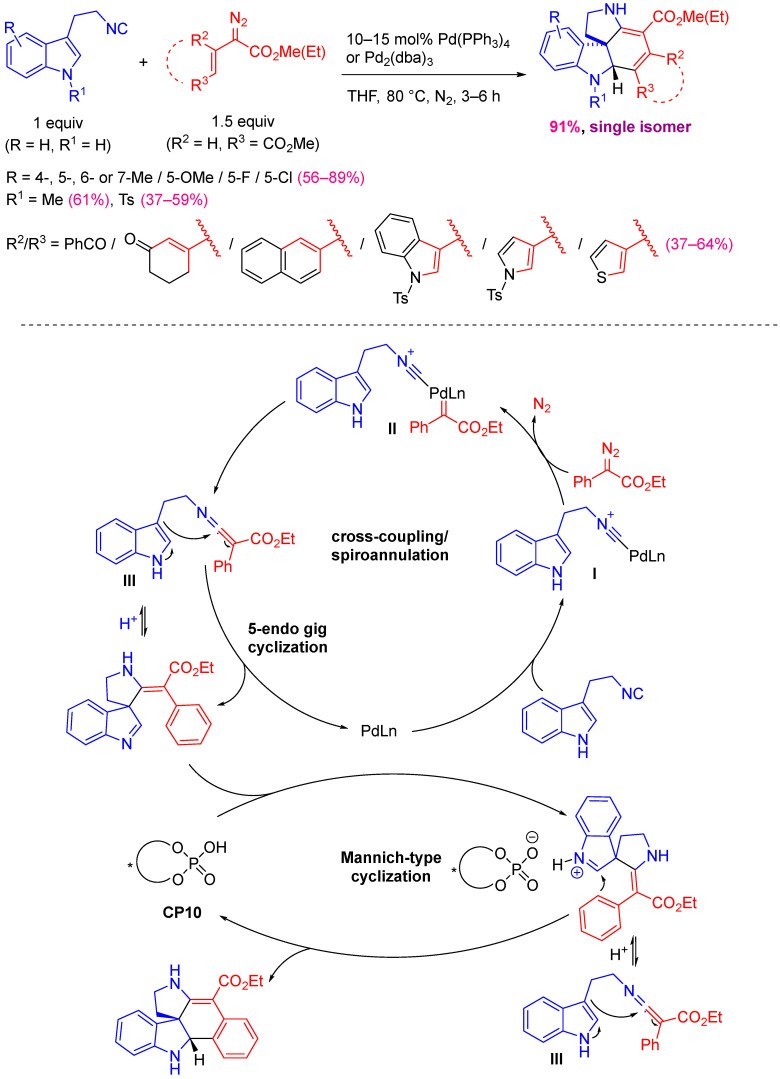
Chiral-phosphoric-acid Mannich-type spirocyclization to pentacyclic spiroindolines (**upper**) and one-pot synthesis of tetracyclic spiroindolines (**lower**). The asterisk (*) indicates the omitted remainder of the CPA catalyst (**CP10**) depicted in the reaction scheme.

Chen et al. combined Pd-catalyzed spiroindolenine formation with a chiral-phosphoric-acid Mannich-type spirocyclization to access pentacyclic spiroindolines (Figure 22; Table S19). 3-(2-Isocyanoethyl)indoles and 1.5 equiv *α*-diazo esters (Pd(PPh_3_)_4_ or Pd_2_(dba)_3_, THF, 80 °C) give spiroindolenines, which a CPA (*S*) in 1,2-DCE (−10 or 0 °C, 4–6 d) converts to pentacyclic products. EWGs and EDGs on indole and aryls had minor influence (31–71%, 82:18–93:7 *er*), except *o*-F aryl failed. One-pot synthesis of tetracyclic spiroindolines under the standard conditions was successful, whereas isocyanides tolerated various substrates regardless of electronics and position. Additionally, *N*-TFA, *N*-Bn and *N*^2^-Boc protected spiroindoline derivatives were developed with good yields. Mechanistically, Pd carbene/isocyanide couples to a ketenimine, indole attacks the spiroindolenine, then acid-mediated reversible ring-opening/Mannich cyclization occurs [38].

**Figure 23:**
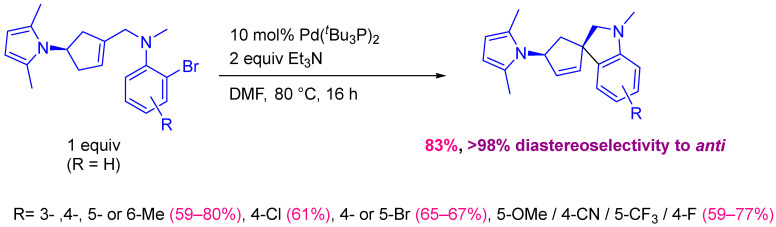
Palladium-catalyzed synthesis of spiroindolines.

Lindman et al. reported a diastereoselective intramolecular Mizoroki-Heck annulation building *N*-methylspiroindolines with a quaternary spirocenter from (+)-Vince-lactam-derived cyclopentenyl 2-bromo-*N*-methylanilines (Figure 23; Table S20). With Pd(*^t^*Bu_3_P)_2_/Et_3_N in DMF, the model gave 83% with >98% *anti*-selectivity. *N*-methylation of aniline was needed for substrate stability. *N*-methylspiroindolines were synthesized (59–81%, >98% *anti*-insertion); electron-rich and -poor anilines and ring-methyl/halo substituents were tolerated (C-Cl retained), and an azaindoline was synthesized from an aminopyridine-derived substrate (81%). The method also allowed the preparation of the enantiomeric series starting from (-)-Vince lactam with similar stereoselectivity. Products were elaborated to protected unnatural amino acids (64%) through benzyloxycarbonylation. DFT attributes the *anti*-selectivity to a 2.9 kcal/mol-lower migratory-insertion barrier under Curtin–Hammett conditions [39].

**Figure 24:**
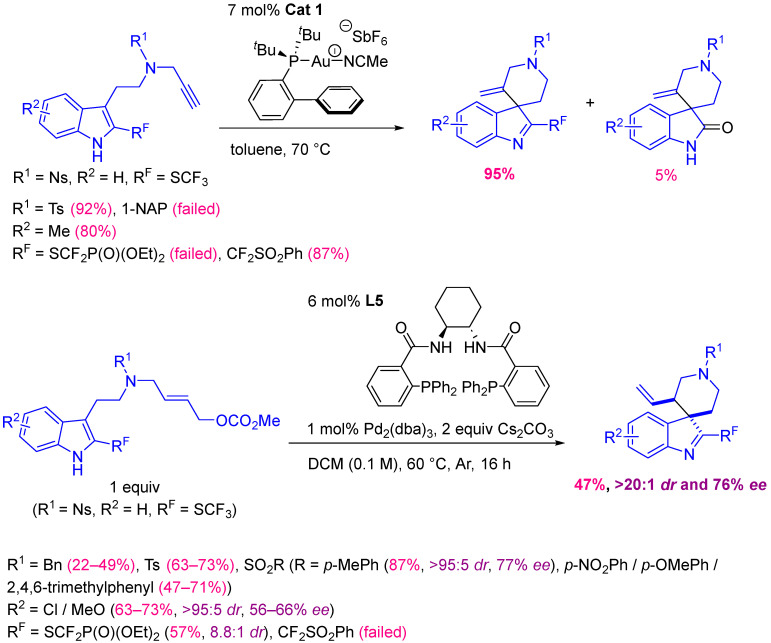
Au(I)-catalyzed cycloisomerization and Pd(0)-catalyzed asymmetric cyclization.

Buttard et al. accessed fluorinated spiroindolenines by utilizing Au(I)-catalyzed cycloisomerization and Pd(0)-catalyzed asymmetric cyclization of tryptamine derivatives (Figure 24; Table S21). Au catalysis (Cat 1, toluene, 70 °C) converted an SCF_3_-tryptamine to the spiroindolenine in 95% (vs. a spiroindolinone byproduct 5%); Ns/Ts gave high yields, while 1-NAP failed. CO_2_Et substitution on the ethyl spacer gave a 71% yield with 100:0 *dr*. The Pd route (Pd_2_(dba)_3_, L5, Cs_2_CO_3_, 0.1 M DCM, 60 °C) gave spiroindolenines up to 87%, >95:5 *dr* and 77% *ee*; a higher yield of 61% was achieved with 0.05 M DCM, however, with a reduction in the stereoselectivity (19:1 *dr* and 68% *ee*). The two manifolds showed complementary tolerance of fluorinated groups (e.g., CF_2_SO_2_Ph excelled in Au but failed in Pd; SCF_2_P(O)(OEt)_2_ the reverse) [40].

#### 3.1.3. Copper-Catalyzed Methods

**Figure 25:**
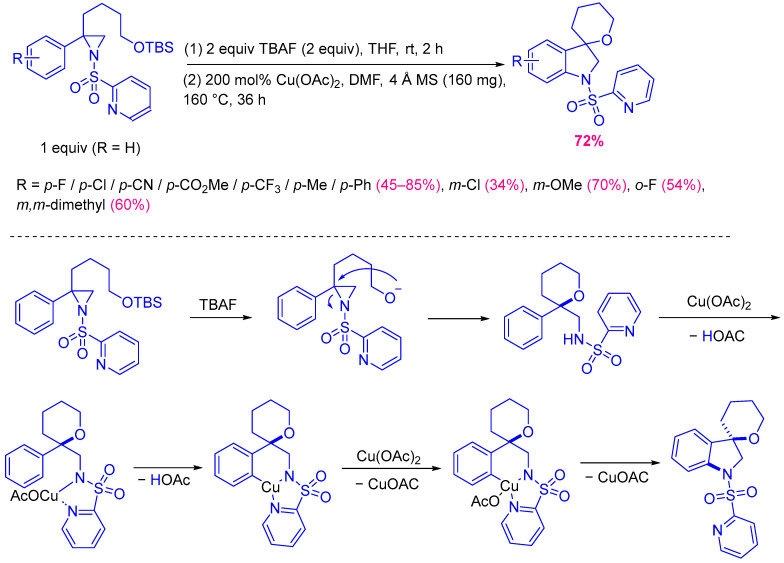
Sequential aziridine ring-opening and Cu-mediated C–H activation/cyclization.

Xing et al. reported a stepwise route to spiroindoline pyrans and furans via aziridine ring-opening followed by Cu-mediated C-H activation/cyclization, building two new rings and C-O/C-N bonds from *N*-sulfonylaziridines (Figure 25; Table S22). Phenylboronic acid is first reacted with an alkyne to form an alkene (70%) in the presence of Pd(PPh_3_)_4_ and AcOH in 1,4-dioxane at 80 °C. The alkene is then reacted with sulfonamide using NBS, MnSO_4_ and K_2_CO_3_ in DCM at room temperature to provide the aziridine ring (35%). After TBAF-triggered ring-opening to a pyran intermediate, Cu(OAc)_2_ (200 mol%)/4 Å MS in DMF at 160 °C gave the spiroindoline in 72%. *Para*-substituted aryls gave 45–85% (*m*-Cl low at 34%). The one-carbon-shorter alkyne substrate furnished spiroindoline furans (50%), where *p*-Cl (48%) and *p*-Ph (43%) substituents on the phenyl ring gave similar yields, while Me substitution on the pyridine moiety increased the yield to 63%, unlike in spiroindoline pyrans where it had no significant effect. Varied tethers linking the aziridine to OTBS gave 50–75%. Desulfonylation/Boc protection (85%) and gram-scale (65%) were demonstrated. Mechanism involved pyran intermediate-Cu coordination, intramolecular C-H activation, Cu-promoted oxidation, then reductive elimination [41].

**Figure 26:**
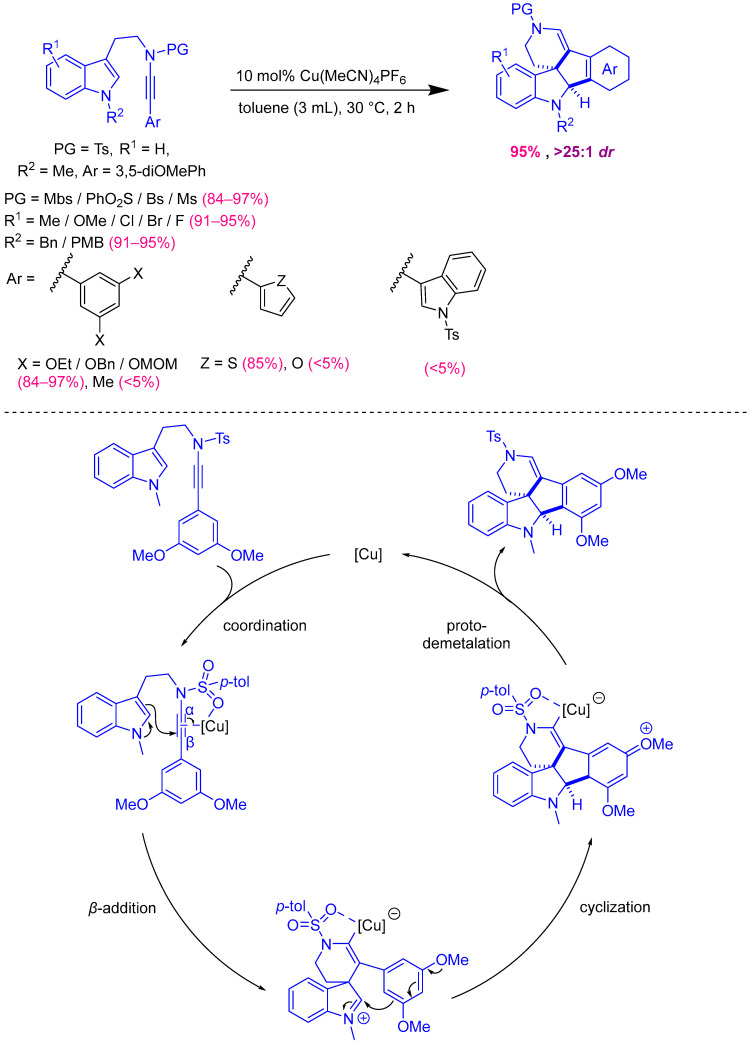
A copper-catalyzed dearomative cyclization.

Xu et al. reported a Cu-catalyzed dearomative cyclization of indolyl ynamides with regioselective *β*-addition, giving pentacyclic spiroindolines without noble metals or stoichiometric oxidants (Figure 26; Table S23). With Cu(MeCN)_4_PF_6_ in toluene at 30 °C, an electron-rich (3,5-dimethoxyphenyl) substrate reached 95%. Various *N*-sulfonyl groups and indole-ring EDGs/EWGs gave 84–97%; electron-rich aryl ynamides were required (thienyl 85%; furan/indole failed). Aniline-containing indolyl ynamide was not explored due to failed synthesis, steric hindrance prevented coupling of the sulfonamide with alkyne/alkynyl bromide. All products showed >25:1 *dr*; chiral Cu gave preliminary 75–80% yield, 48–60% *ee*. Mechanistically, Cu coordinates the ynamide/sulfonyl to enforce *β*-cyclization to a vinyl–copper, intramolecular arene attack, then protodemetalation [42].

**Figure 27:**
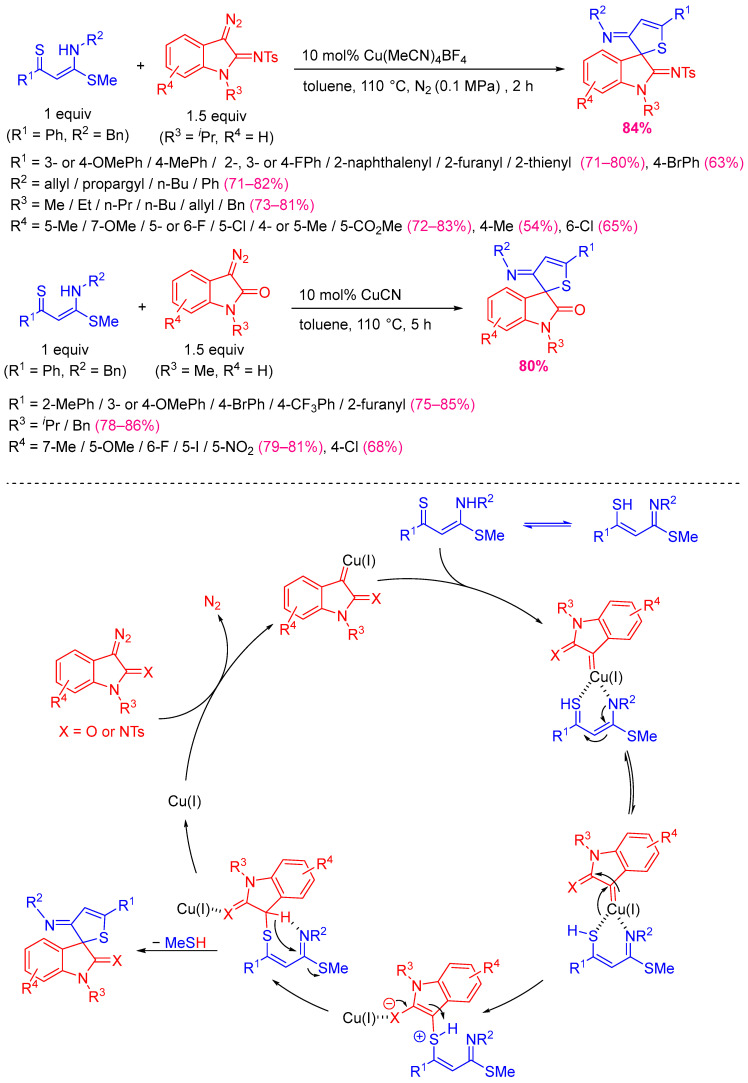
A copper(I)-catalyzed [4 + 1] annulation strategy.

Huang et al. developed a Cu(I)-catalyzed [4 + 1] annulation of enaminothiones with indoline-based diazo compounds, giving spiroindoline-fused *S*-heterocycles (Figure 27; Table S24). With Cu(MeCN)_4_BF_4_ in refluxing toluene, the model gave 84%; other metals (Rh/Pd/Ag/Ir) failed. An *N*-EDG on the indole was important (*N*-H/*N*-Boc failed); most ring substituents gave 72–83%, while the sterically hindered 4-Me required prolonged time with lower yield (54%). The reaction extends to 3-diazooxindoles with CuCN catalysis, using similar conditions but different duration. It is worth noting that 3-diazoindolin-2-imines exhibited higher reactivity compared to their corresponding 3-diazooxindoles. Produced spiroindolines can undergo hydrolysis and copper-catalyzed azide–alkyne cycloaddition (CuAAC) to generate triazole derivatives. Mechanism includes Cu(I) carbene formation, enaminothione coordination/tautomerization, transmetalation and S-H insertion, then intramolecular annulation releasing methanethiol [43].

#### 3.1.4. Gold-Catalyzed Methods

**Figure 28:**
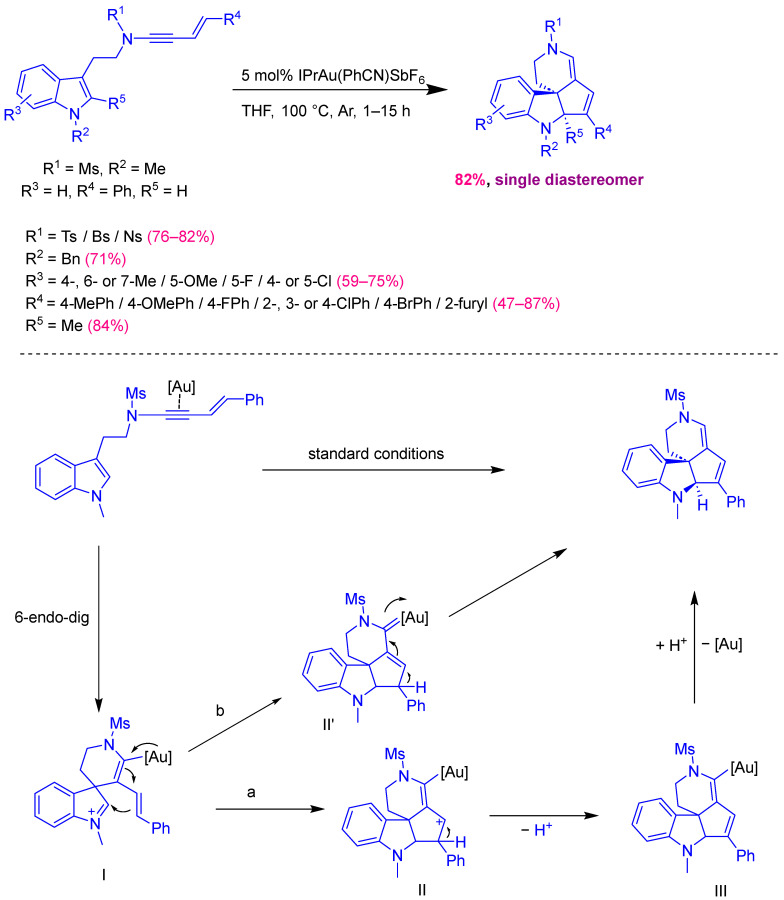
A gold-catalyzed *β*-regioselective cycloisomerization.

Zhu et al. reported a gold-catalyzed *β*-regioselective cycloisomerization of tryptamine-derived enynamides, giving tetracyclic [6,5,5,6] spiroindolines (Figure 28; Table S25). Replacing a propargyl with a conjugated enyne switches regioselectivity completely; with IPrAuPhCN·SbF_6_ in THF at 100 °C, a single diastereomer formed in 82%. Silver salts and Brønsted acids were ineffective and solvent strongly mattered. Sulfonyl *N*-protecting groups on the amide moiety (76–82%), indole-ring 4–7 substitution (59–75%) and aryl substituents (47–87%) were tolerated, while alkyl in place of the aryl gave complex mixtures where desired product isolation failed. Subsequent spiroindolines hydrogenation/deprotection were demonstrated. Mechanism involved Au-promoted *β*-selective 6-endo-dig cyclization to an iminium intermediate, intramolecular cyclization, then protodeauration; a vinylgold intermediate was isolated supporting this pathway, while a possible gold carbene route cannot be ruled out [44].

**Figure 29:**
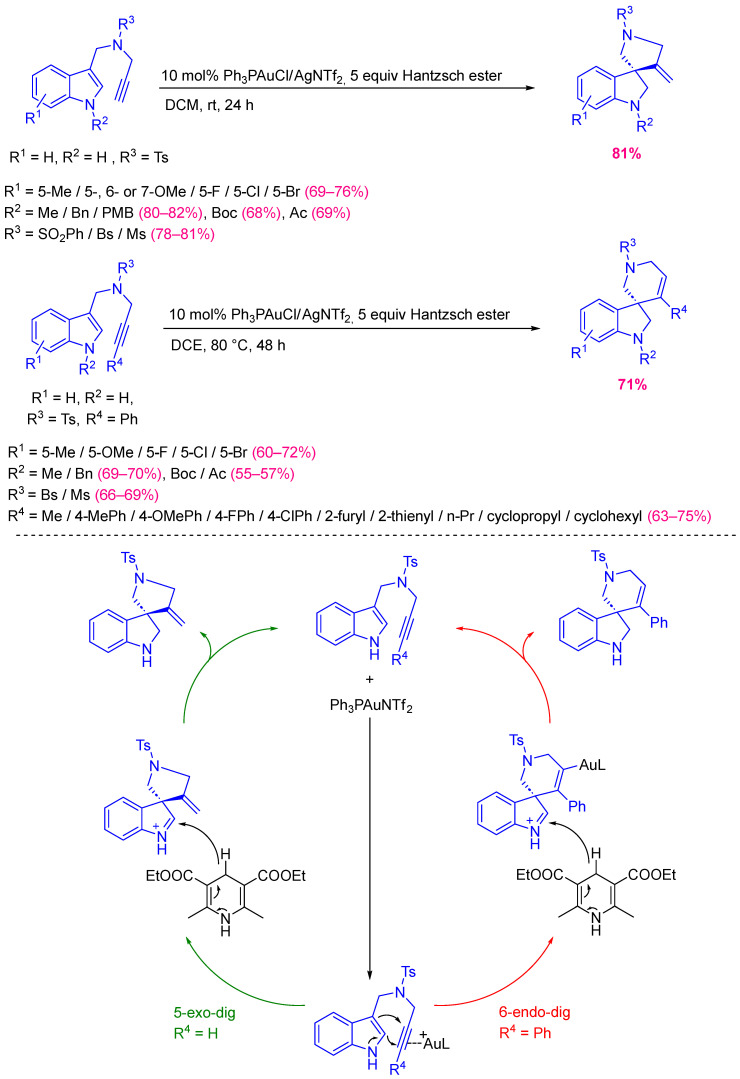
A gold(I)-catalyzed substitution-controlled 5-exo-dig cyclization (**upper**) and 6-endo-dig (**lower**) cyclization.

Zhu et al. developed Au(I)-catalyzed substitution-controlled cycloisomerization/Hantzsch ester in situ reduction that divergently yields spiro[indoline-3,3′-pyrrolidine] (5-exo-dig) or spiro[indoline-3,3′-piperidine] (6-endo-dig) frameworks, with selectivity set solely by the alkyne terminus (H vs. aryl/alkyl) (Figure 29; Table S26). With Ph_3_PAuCl/AgNTf_2_ and Hantzsch ester, the 5-exo and 6-endo series gave 81% and 71%; insufficient Hantzsch ester triggered competing Wagner-Meerwein rearrangement. 6-Endo series required elevated temperature (31% at room temperature) and DCE instead of DCM. In the 5-exo-dig pathway, indole’s *N*-EDG afforded better yields (80–82%) than *N*-EWG (68–69%), even after requiring higher temperatures (80 °C). In the 6-endo-dig pathway, *N*-substituents followed a similar trend (EDG 69–70% vs. EWG 55–57%). The authors noted that the 6-endo series’ lower yields were typically due to unreacted starting material. Moreover, DFT rationalizes the switch (5-exo favored for terminal alkynes by 3.3 kcal/mol; 6-endo for aryl by 4.2 kcal/mol), selectivity attributed to steric hindrance from the phenyl and electronic effects (NPA charge distribution) on the alkyne carbons. The mechanism involves gold-catalyzed alkyne activation followed by either a 5-exo-dig cyclization (R^4^ = H) or a 6-endo-dig cyclization (R^4^ = aryl/alkyl) to form different spiroindoleninium intermediates, which undergo reduction via Hantzsch ester to give spiroindolines [45].

**Figure 30:**
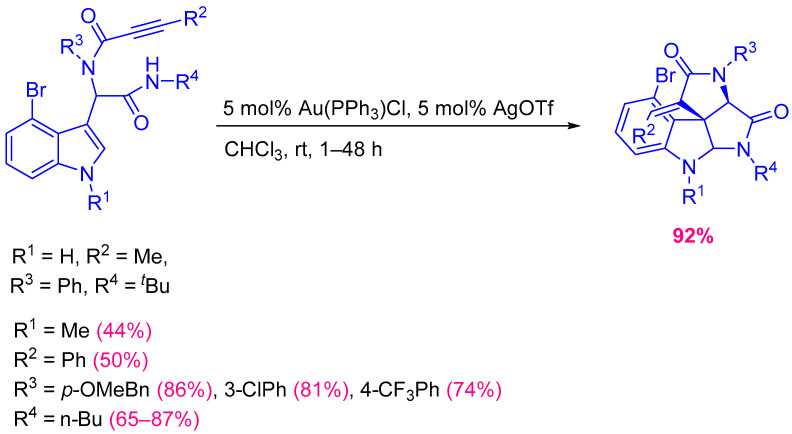
Cationic gold-catalyzed cascade cyclization.

Zaman et al. reported a divergent post-Ugi strategy in which 4-bromo-1*H*-indole-3-carbaldehyde-derived Ugi propargylamides give tetracyclic spiroindolines under cationic Au(I) catalysis (or azepino[3,4,5-*cd*]indoles via Pd-catalyzed reductive Heck) (Figure 30). The required Ugi adducts are obtained by a four-component coupling of 4-bromo-1*H*-indole-3-carbaldehydes, propiolic acids, amines, and isocyanides in MeOH at room temperature for 24 h, delivering propargylamides in 65–98% yield. With Au(PPh_3_)Cl/AgOTf in CHCl_3_ at room temperature, the spiroindoline formed in 87% on 0.3 mmol scale (92% on 1 mmol). Alternative catalysts or solvents were not examined for this gold manifold. *Para*-methoxybenzyl/3-chlorophenyl amines gave high yields, the *p*-CF_3_ substrate that failed in the Pd route gave 74%, while *N*-methyl indole was sluggish (44%). The intact aryl bromide enabled late-stage Suzuki coupling to install a variety of aryl and heteroaryl substituents in high yields, and by Sonogashira coupling of a representative spiroindoline with phenylacetylene to introduce an alkynyl pendant. A possible mechanism involves cationic Au activating the alkyne for indole Friedel–Crafts-type *ipso*-attack and an *anti*-intramolecular nucleophilic capture of the resulting iminium species by the amide nitrogen, which is consistent with the (*E*)-configuration observed in the spiroindolines [46].

#### 3.1.5. Silver-Catalyzed Methods

**Figure 31:**
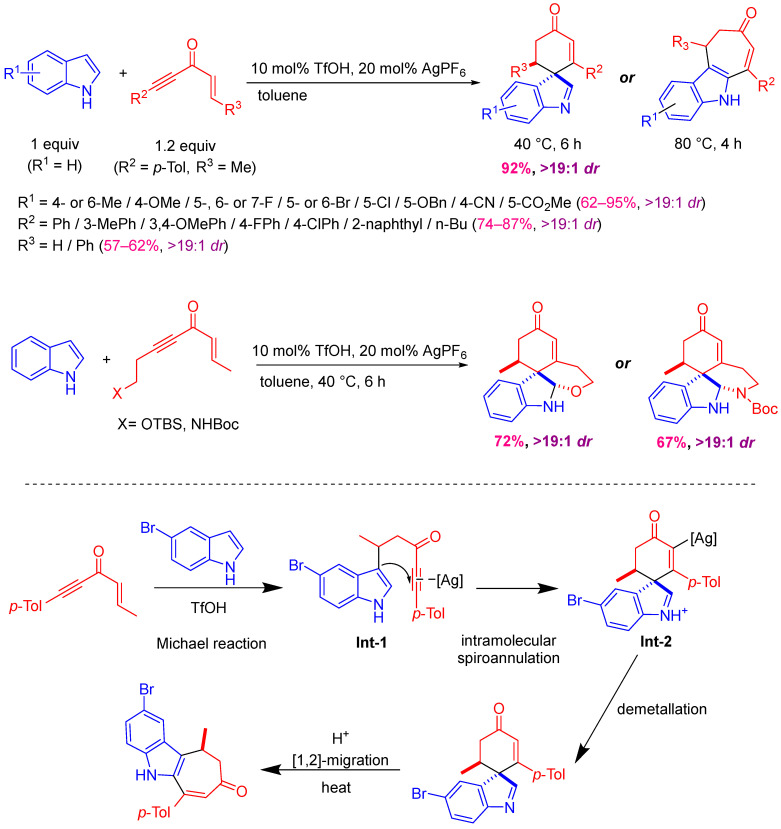
A temperature-controlled divergent strategy based on Brønsted acid and silver cooperative catalysis (**upper**) and cascade annulation to tetracyclic spiroindolines (**lower**).

Dong et al. reported a Brønsted-acid/silver cooperative, temperature-controlled divergence between spiroindolenines and indole-fused products from indoles and enynones (Figure 31; Table S27). With AgPF_6_/TfOH in toluene at 40 °C, spiroindolenines formed in 92% (>19:1 *dr*); raising the temperature to 60–80 °C switched to dihydrocyclohepta[*b*]indolones. Indole EDG/EWG gave 62–95%, though C2-substituted indoles failed; enynone variation gave 57–87%. Spiroindolenines reduction by NaBH_4_/NaBH_3_CN gave spiroindolines in good yields. Nucleophile-bearing enynones enabled [5 + 1]/Mannich cascades to tetracyclic spiroindolines (67–72%). Mechanism includes acid-catalyzed Michael addition, Ag-activated dearomative spiroannulation, and then demetalation/proton transfer [47].

**Figure 32:**
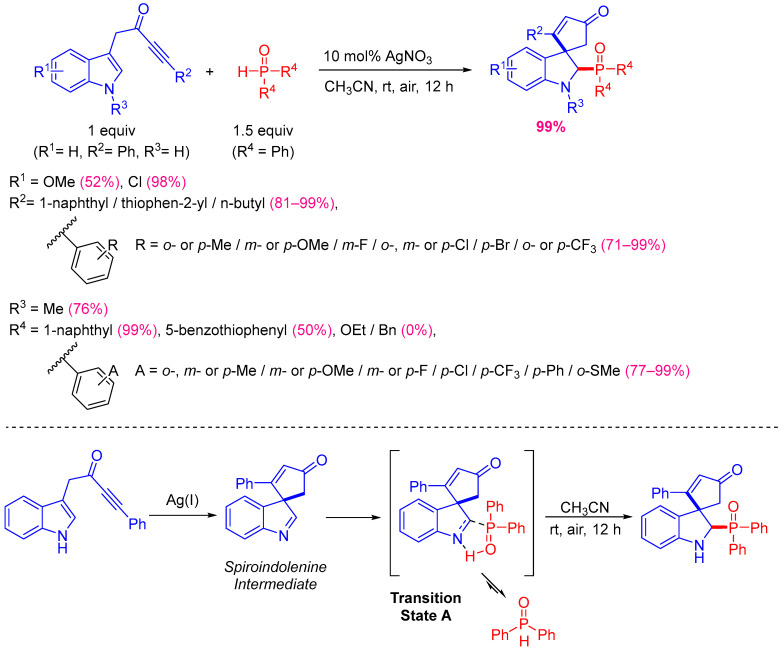
A tandem dearomatization–spirocyclization–nucleophilic addition strategy for constructing 2-phosphonylated C3-spirocyclic indolines.

Duan et al. reported a tandem dearomatization–spirocyclization-nucleophilic addition of indolyl ynones giving 2-phosphonylated C3-spiroindolines, using weakly nucleophilic phosphine oxides (Figure 32; Table S28). With AgNO_3_ in MeCN (rt, air), the model reached 99% (AgOTf/DCM equally effective). Diverse ynone aryl/alkyl termini and indole substituents gave 52–99%, and symmetrical diaryl phosphine oxides 50–99%, while dialkoxy/dibenzyl phosphine oxides failed. The reaction scaled to 1.4 g (78%) and the ketone was reducible (86%). Mechanism involves Ag-catalyzed spiroindolenine formation, then addition of the isomerized hydroxydiphenylphosphane through transition state A [48].

**Figure 33:**
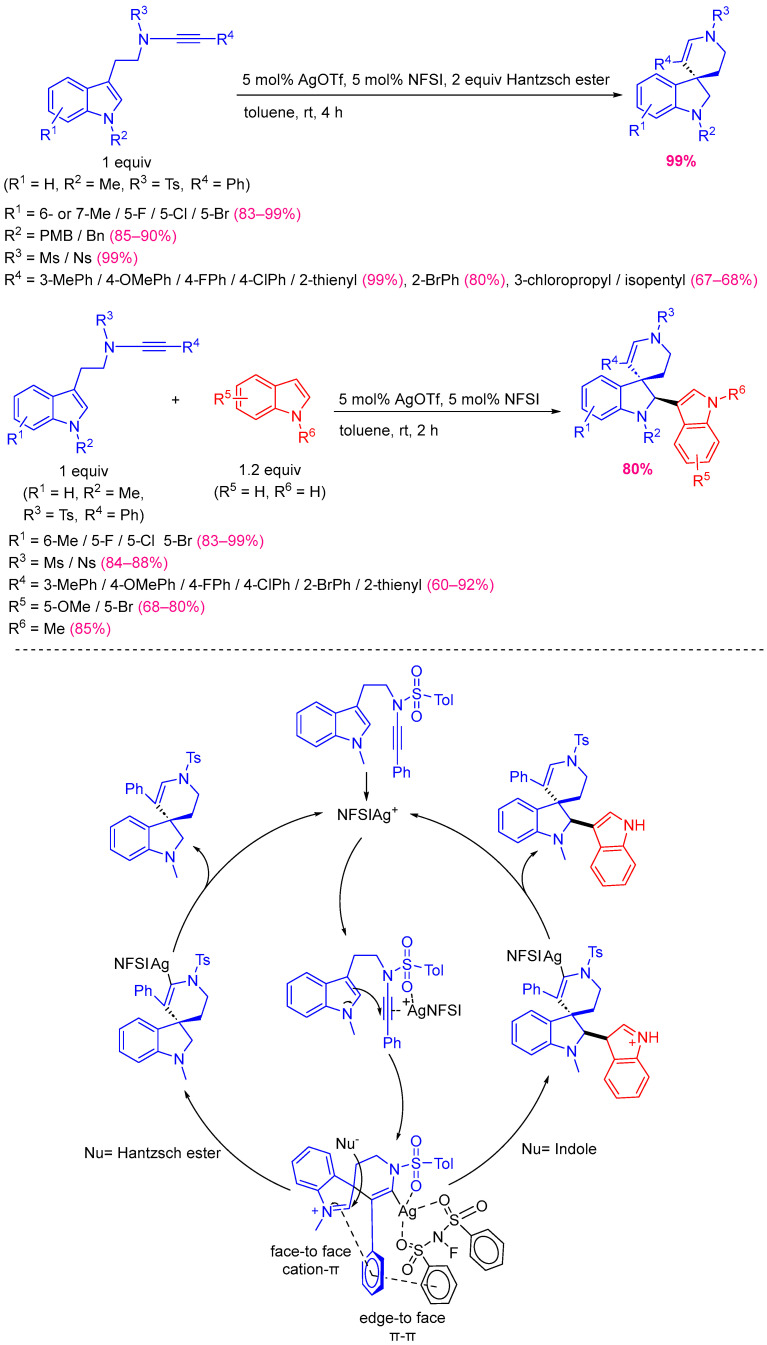
A novel silver triflate/*N*-fluorobenzenesulfonimide (AgOTf/NFSI) catalytic system using Hantzsch ester (**upper**) and indole derivatives as nucleophiles (**lower**).

Liang et al. developed an AgOTf/NFSI (silver triflate/*N*-fluorobenzenesulfonimide) system for spiro[indoline-3,4′-piperidine]s from tryptamine-ynamides, in which NFSI acts not as a fluorinating agent but as a ligand whose cation-π-π interaction stabilizes the spiroindoleninium and suppresses Wagner–Meerwein rearrangement (Figure 33; Table S29). With Hantzsch ester as hydride donor in toluene (rt), the model reached 99% in 4 h. In the scope of ynamides, aryl alkynes and indole-ring halides gave up to 99%; bulky *N*-protection needed higher loading and alkyl alkynes were less efficient (67–68%). Indole nucleophiles gave 60–99%. Mechanism contains chelation-controlled *β-*addition to a spiroindoleninium, NFSI-shielded face-selective nucleophile delivery, then protodemetalation [49].

**Figure 34:**
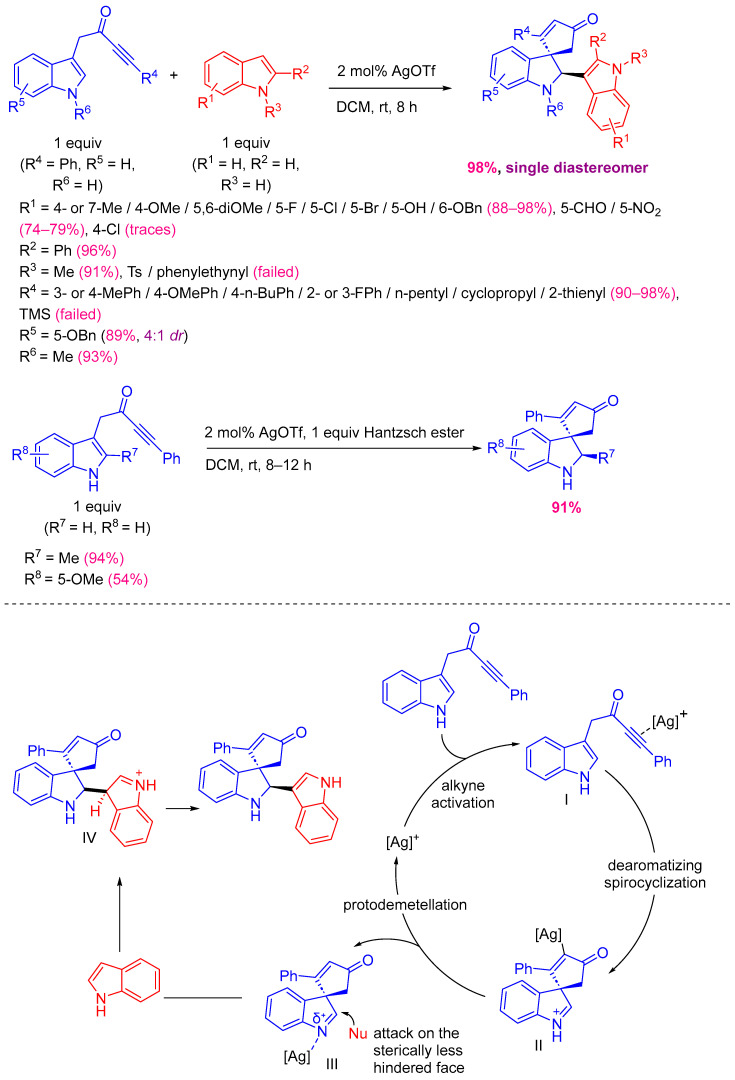
A silver(I)-catalyzed dearomatizing spirocyclization/nucleophile addition using indole nucleophiles (**upper**) or Hantzsch ester (**lower**).

Bag and Sawant reported a Ag(I)-catalyzed dearomatizing 5-endo-dig spirocyclization of indole-tethered ynones followed by *C*-nucleophiles or hydride trapping (Figure 34; Table S30). With 2 mol% AgOTf in DCM, the model gave 98% as a single diastereomer; Cu/Au catalysts were inferior or inactive. Indole nucleophiles and diverse ynone termini performed well, while 4-chloroindole and C2-substituted ynones failed. Pyrrole, *N*-phenylpyrrole, 2-methoxythiophene and arene azulene were successful as nucleophiles (77–91%), while 7-azaindole, indazole, benzotriazole, 2-methylfuran, 1-methylpyrazole and 1,3,5-trimethoxybenzene failed (0%). Hantzsch ester gave spiroindolines in up to 94%. A single diastereomer was observed in almost all cases. Mechanism includes π-acid-promoted spirocyclization to a spiroindolenine, then face-selective nucleophile addition/rearomatization [50].

**Figure 35:**
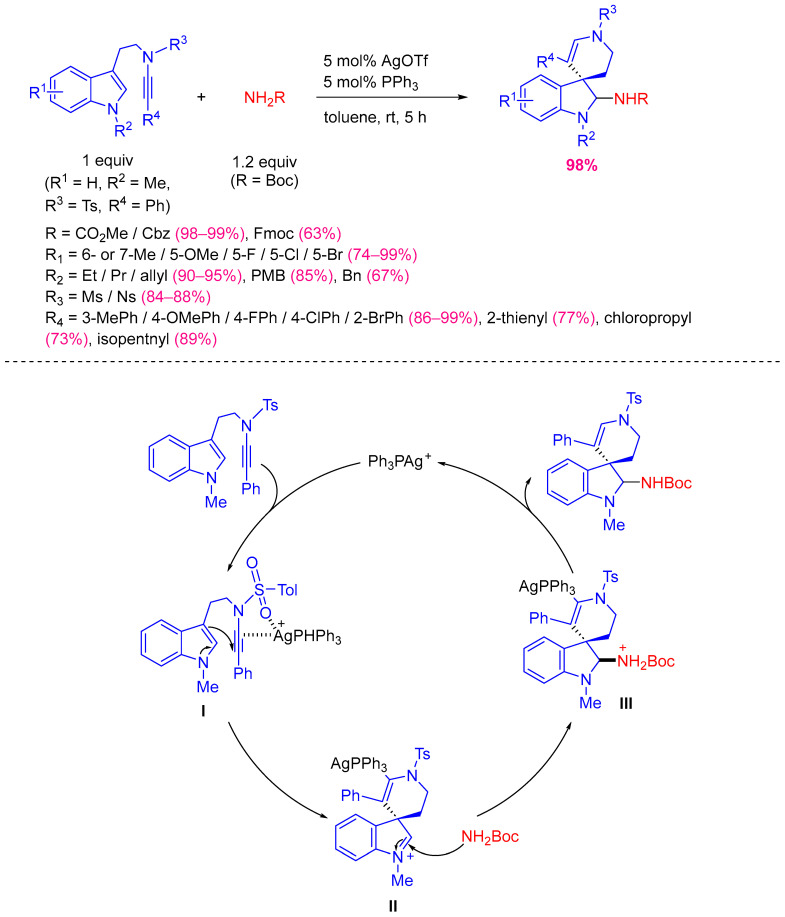
Spiro[indoline-3,4′-pyridin]-2-yl carbamates through an AgOTf/PPh_3_-catalyzed tandem cyclization.

Liang et al. reported a diastereoselective AgOTf/PPh_3_-catalyzed tandem cyclization of tryptamine-ynesulfonamides giving spiro[indoline-3,4′-pyridin]-2-yl carbamates, with carbamates as external nucleophiles trapping the spiroindoleninium and suppressing azepino[4,5-*b*]indole formation (Figure 35; Table S31). In toluene at room temperature, the model reached 98%; the PPh_3_ ligand was decisive. Indole-ring halides/EDGs (74–99%) and *N*-alkyl groups (90–95%) were tolerated. Meanwhile, EWGs on the indole’s phenyl such as ester and sulfonamide failed. Varied carbamates (H_2_NCO_2_Me, H_2_NCbz) gave near-quantitative yields, favoring less bulky substrates. Mechanism involves Ag/PPh_3_-activated cyclization to a spiroindoleninium, then face-selective carbamate trapping/protodemetalation [51].

#### 3.1.6. Other/Mixed Metal Catalysis

**Figure 36:**
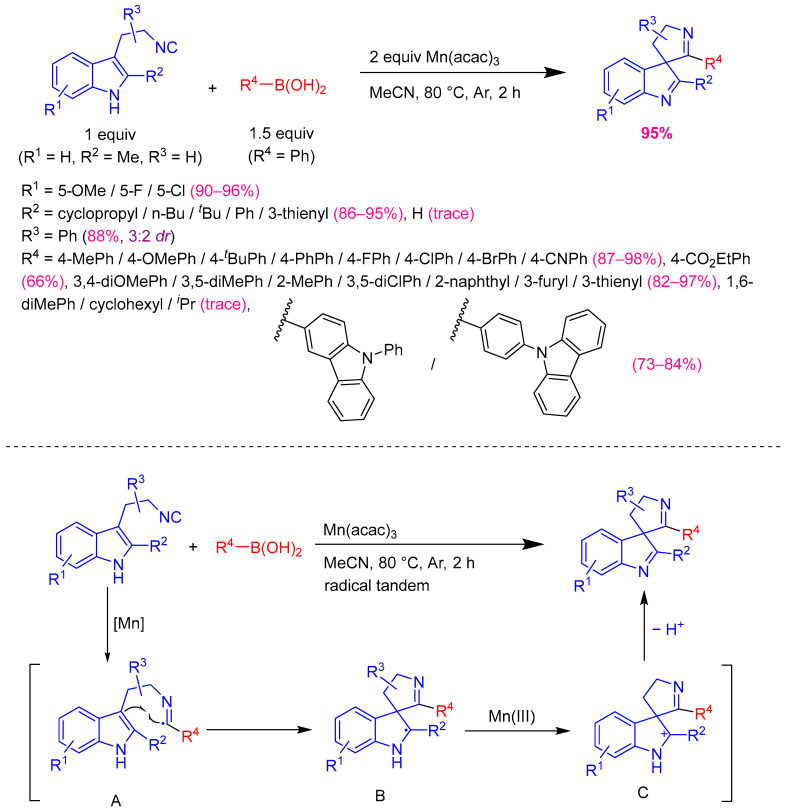
Mn(III)-mediated radical addition/spirocyclization cascade for the synthesis of spiroindoline derivatives.

Jiang et al. reported a Mn(III)-mediated radical addition/spirocyclization of tryptamine-derived isocyanides with arylboronic acids giving spiroindolines (Figure 36; Table S32). With Mn(acac)_3_ in MeCN at 80 °C (Ar), the model reached 95%. C2 substitution on the isocyanide was required (C2-H and *N*-methyl substrates failed); 5-substituted and 2-substituted indoles gave 86–96%, and aryl/heteroaryl boronic acids 66–98%, while alkyl/sterically hindered boronic acids failed. Reduction (with BH_3_·NH_3_) gave *cis*-spiroindoline (61%, >20:1 *dr*). Addition of 2 equiv of TEMPO suppressed product formation. Mechanistic control experiments support the proposed radical pathway: radical addition to the isocyano group then C3 spirocyclization [52].

**Figure 37:**
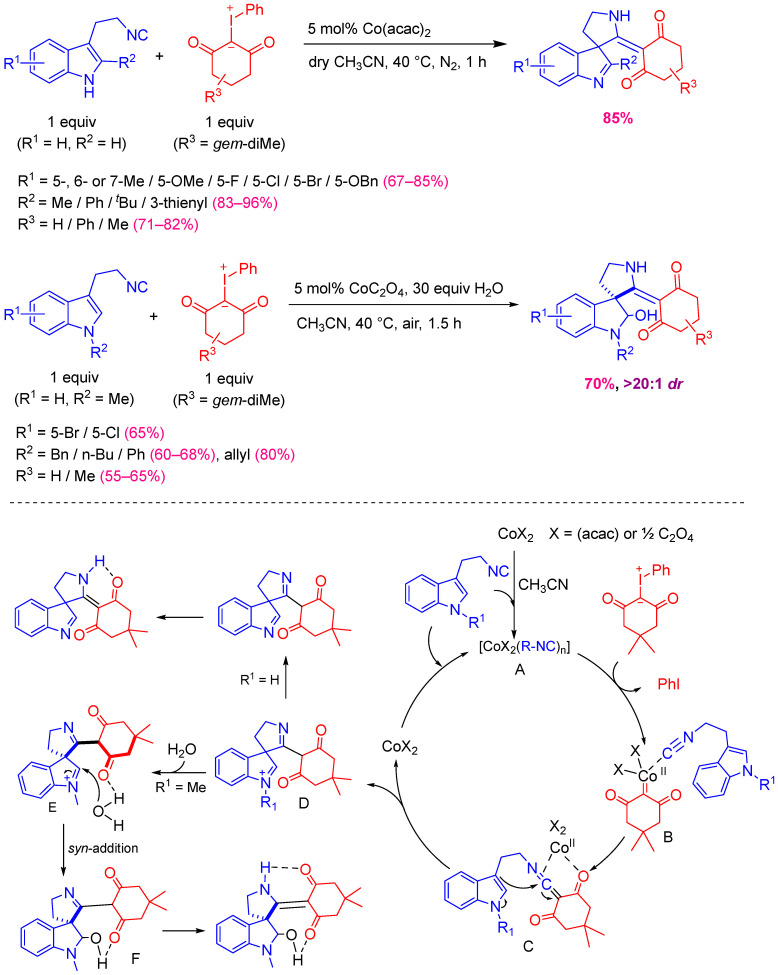
Cobalt(II)-catalyzed coupling-spirocyclization reactions.

Yuan et al. reported a Co(II)-catalyzed coupling-spirocyclization of tryptamine-derived isocyanides with iodonium ylides giving stable spiroindolines (Figure 37; Table S33). With Co(acac)_2_ in MeCN at 40 °C, the model gave 85%; C2-H and C2-substituted isocyanides, varied indole substituents, and diverse ylides gave 67–96%. Phenyl substitution at the isocyanide’s side chain had little effect on yield with low diastereoselectivity (69%, 2:1 *dr*). Additionally, a cyclopentane-derived ylide proceeded smoothly (86%) and ylides derived from cyclic ester/amide were also well tolerated (73–95%). Switching to *N*-substituted indoles with added water and CoC_2_O_4_ triggered a rare *syn* coupling/spirocyclization/hydration cascade (>20:1 *dr*). The produced spiroindolines could undergo despirocyclization with amines to enamines. Mechanism includes Co-carbene/isocyanide coupling to a ketenimine, C3 attack, then 1,3-H shift or H-bond-directed *syn* hydration [53].

**Figure 38:**
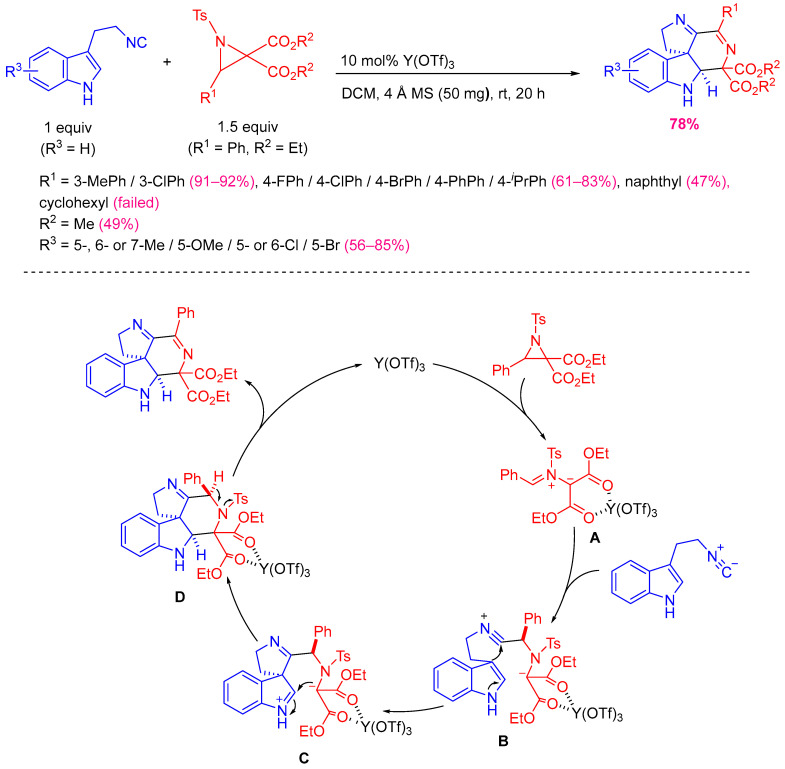
Yttrium(III)-catalyzed cascade reaction of 3-(2-isocyanoethyl)indoles with 2,2′-diester aziridines.

Li et al. reported yttrium(III)-catalyzed cascade of 3-(2-isocyanoethyl)indoles with 2,2′-diester aziridines giving tetrahydro-*β*-carboline-fused polycyclic spiroindolines via ring-opening/Friedel-Crafts/Mannich/desulfonylation (Figure 38; Table S34). With Y(OTf)_3_/4 Å MS in DCM, products formed in 47–92%; a Lewis acid was essential, and Y outperformed Sc/Zn/Yb/Mg. *Meta*-aryl aziridines gave up to 92% and *para*-substituted 61–83%, while alkyl aziridines failed; indole substituents gave 56–85%. Mechanism involves Lewis-acid-promoted aziridine C-C cleavage to a dipole, isocyanide addition to a nitrilium, Friedel-Crafts spirocyclization, *cis*-selective Mannich, and desulfonylation [54].

**Figure 39:**
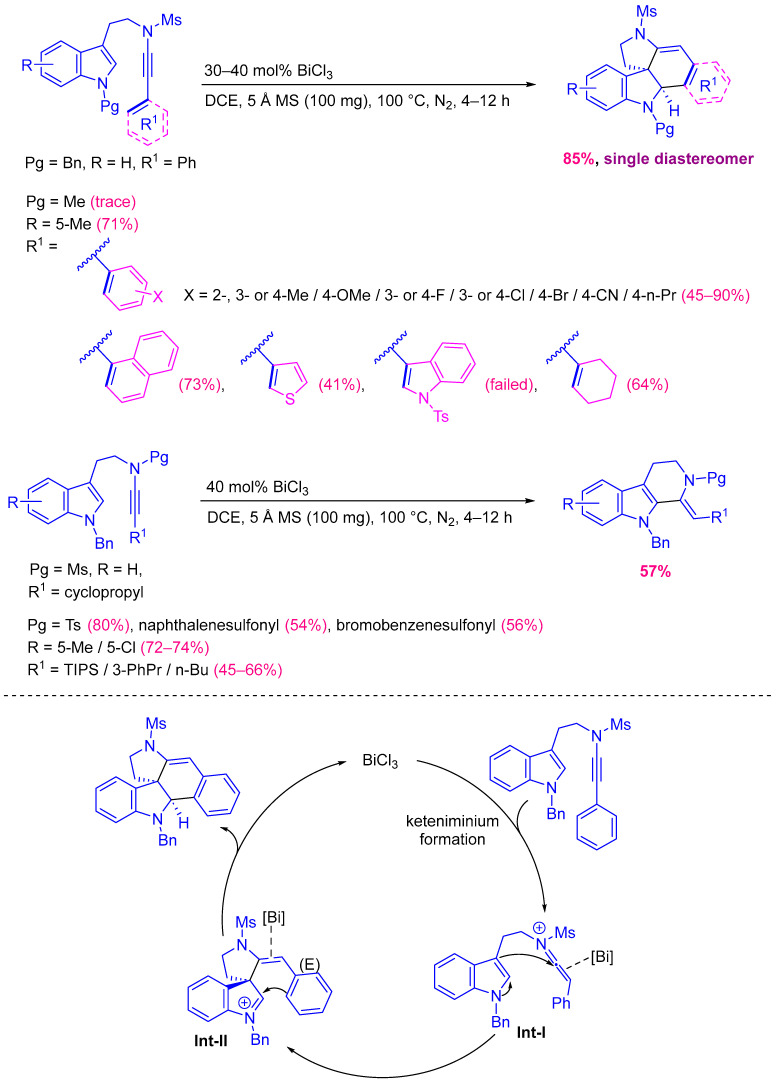
BiCl_3_-mediated tandem cyclization of tryptamine-derived ynamides.

Lin et al. reported a BiCl_3_-mediated tandem cyclization of tryptamine-derived ynamides giving pentacyclic spiroindolines (or tricyclic indoles, depending on the ynamide terminus) (Figure 39; Table S35). With BiCl_3_/5 Å MS in DCE at 100 °C, the *N*-benzyl substrate gave 85% as a single diastereomer; an *N*-benzyl group was essential (*N*-methyl gave traces). Ynamides with a phenyl terminus bearing EDG/EWG gave 45–90%, while alkyl ynamides diverted to tricyclic indoles (45–80%). Mechanistically, a BiCl_3_-generated keteniminium is trapped by the terminal aryl group to form a tricyclic iminium, which is trapped further by an adjacent aryl to form the pentacyclic spiroindolines [55].

#### 3.1.7. Lewis Acid/Non-Transition Metal Catalysis

**Figure 40:**
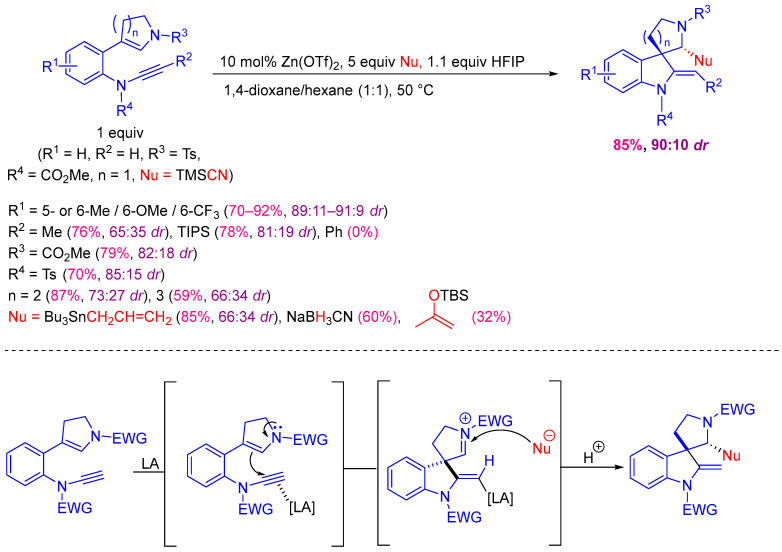
A Lewis acid–catalyzed diastereoselective domino reaction of enamide-ynamides with trimethylsilyl cyanide (TMSCN) or other nucleophiles to construct spiroindolines.

Yamaoka et al. reported a Lewis-acid-catalyzed domino reaction of enamide-ynamides with trimethylsilyl cyanide (TMSCN) giving spiroindolopyrrolidines, where cyanide, as a nucleophile, suppresses Wagner-Meerwein rearrangement (Figure 40; Table S36). With Zn(OTf)_2_/HFIP in dioxane/hexane (1:1 *w*/*w*) at 50 °C, the model gave 85% (90:10 *dr*); solvent strongly tuned *dr*. Aromatic rings with EDG/EWG gave 70–92% (up to 91:9 *dr*). Tosyl/CO_2_Me *N*-protecting groups and six-/seven-membered enamides were tolerated; a phenyl-terminated ynamide failed. Allylstannane (85%, 66:34 *dr*) and NaBH_3_CN (60%) served as alternative trapping reagents/nucleophiles. Mechanism includes Lewis-acid-activated enamide cyclization onto the ynamide to a spirocyclic iminium, then face-selective cyanide addition [56].

**Figure 41:**
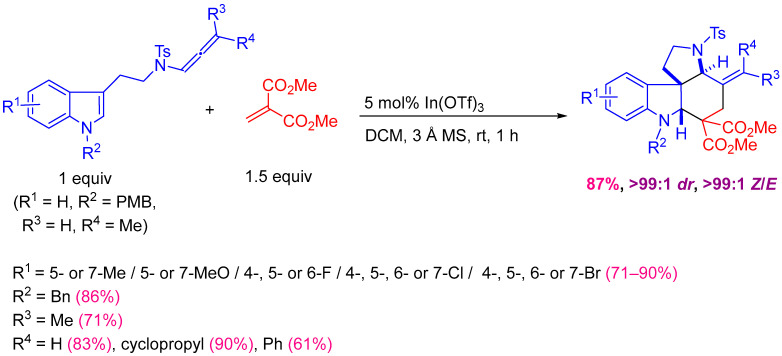
An allenamide-initiated cascade annulation.

Zhao et al. reported an In(OTf)_3_-catalyzed allenamide-initiated [2 + 2 + 2] cascade annulation of tryptamine-derived allenamides with dimethyl methylenemalonate, enabling divergent tetracyclic spiroindoline synthesis (Figure 41; Table S37). With In(OTf)_3_/3 Å MS in DCM, the model gave 87% with *dr* and *Z/E* both >99:1. Indole-ring halides/EDGs gave 71–90%; allenamide substitution (H, phenyl, dialkyl/cycloalkyl) gave 61–90%. Cycloalkyl allenamides yield showed some improvement upon increasing the size of the ring and inserting a heteroatom at the *para* position of the ring. In(III) Lewis acids improved diastereoselectivity and reduced byproducts compared to Cu/Fe/Ni. All reported examples showed excellent stereoselectivity (>99:1 *dr*, >99:1 *Z/E*) [57].

**Figure 42:**
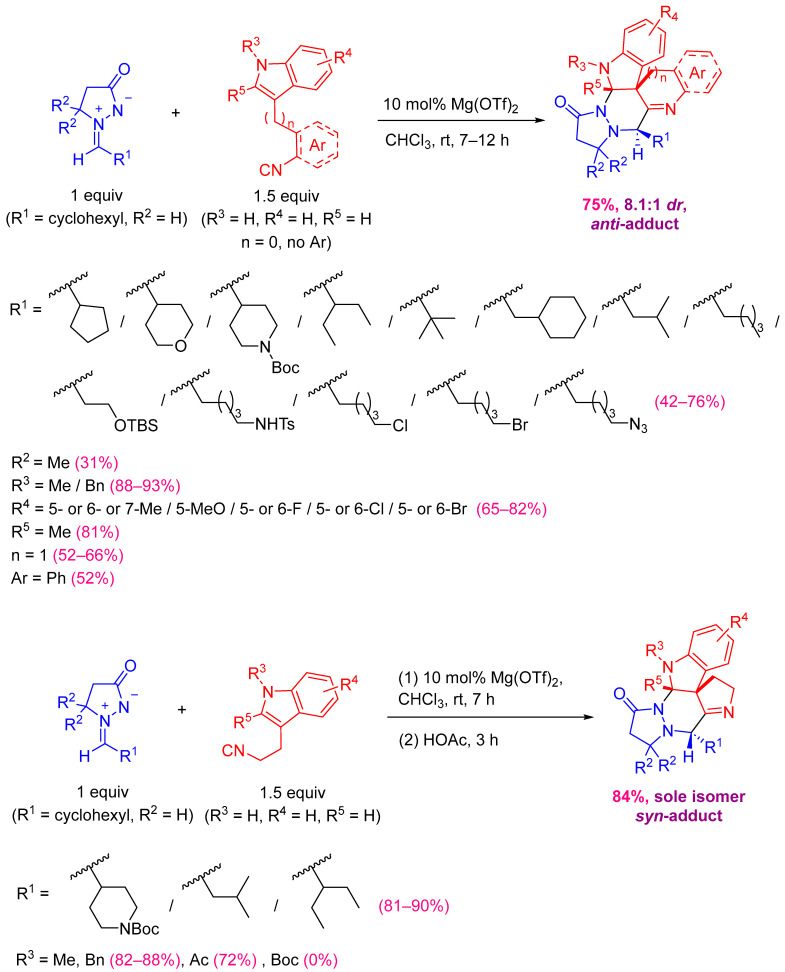
Magnesium(II)-catalyzed cascade reactions of *N*,*N’*-cyclic azomethine imines with indolyl-substituted isocyanides.

Zheng et al. reported a diastereodivergent Mg(II)-catalyzed cascade of *N*,*N’*-cyclic azomethine imines with tryptamine-derived indolyl isocyanides giving pentacyclic spiroindolines (Figure 42; Table S38). With Mg(OTf)_2_ in CHCl_3_, the model gave 75% (*anti:syn* 8.1:1); adding HOAc epimerized cleanly to the *syn* isomer (84%). Regarding *anti* synthesis, diverse azomethine imines were tolerated (31–76%), except for phenyl-substituted variant (failed), and various isocyanides with indole substitution achieved good yields (65–93%), while extending its alkyl spacer and/or incorporating a phenyl gave 52–66%. *Anti*-variants exhibited 2.1–8.4:1 *dr*. Terminally substituted imines (81–90%) were also tolerated with *syn* adducts along with *N*-substituted indolyl isocyanides (72–88%), except *N*-Boc (failed). Gram-scaling was conducted (*anti* 81%). Reduction using LiAlH_4_ (58%), *N*-acetylation (67%) and click triazole synthesis (62%) were successfully conducted on the produced spiroindolines [58].

The metal-catalyzed strategies reported for the synthesis of C3-spiroindolines are summarized in Table 2, highlighting metal catalysts and key conditions used, substrate combinations, reaction efficiencies and stereoselectivities achieved.

### 3.2. Metal-Free/Organocatalytic Methods

#### 3.2.1. Metal-Free Methods

**Figure 43:**
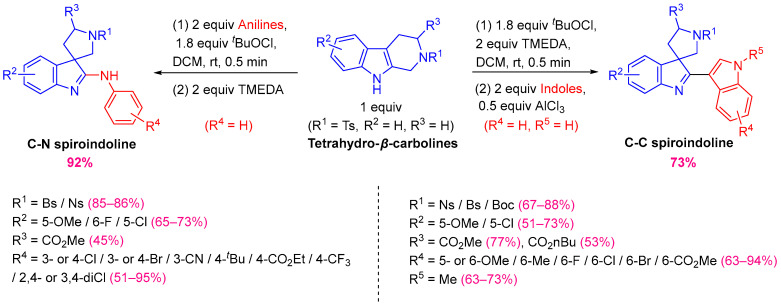
Rearrangement coupling reaction of tetrahydro-*β*-carbolines.

Liu et al. reported a rearrangement coupling of tetrahydro-*β*-carbolines (TH*β*C) giving C-N and C-C spiroindolines via in situ 3-chloroindolenine intermediates (Figure 43; Table S39). With *^t^*BuOCl then TMEDA in DCM, anilines gave C-N spiroindolines (92%, no Lewis acid needed), while indoles required AlCl_3_ for C-C spiroindolines (73%). Diverse TH*β*C protecting groups and ring substituents, anilines bearing EDG/EWG (51–95%), and varied indoles (incl. *N*-Boc) were tolerated. Mechanism involves TMEDA-stabilized 3-chloroindolenine formation, rearrangement, then C2 nucleophilic attack by aniline/indole and coupling to the spiroindoline [59].

**Figure 44:**
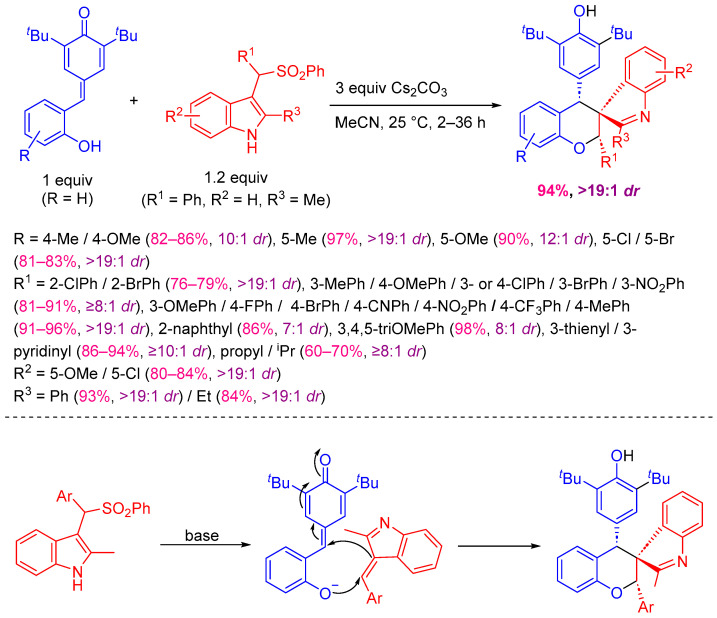
A diastereoselective cyclization strategy for the synthesis of spiroindolenine-bearing chroman scaffolds.

Qin et al. reported a diastereoselective cyclization of *o*-hydroxyphenyl *p*-QM with in situ *α*,*β*-unsaturated imines (from aryl sulfonyl indoles) producing spiroindolenine-chroman scaffolds (Figure 44; Table S40). With Cs_2_CO_3_ in MeCN, spiroindolenine was successfully formed (94%, >19:1 *dr*). C2/C5-substituted indoles gave 80–93%, >19:1 *dr*; *ortho*-substituted aryls improved *dr* over *meta*, and naphthyl/heteroaryl/aliphatic variants were tolerated, but with lower *dr*. The scope of *p*-QM was also studied, C5-substitution (methyl/halogens) achieved higher *dr* (>19:1 *dr*) than C4 (10:1 *dr*). Mechanistically, *p*-QM cyclize with *α*,*β*-unsaturated imines generated from aryl sulfonyl indoles through an oxa-Michael/1,6-conjugated addition in basic conditions [60].

**Figure 45:**
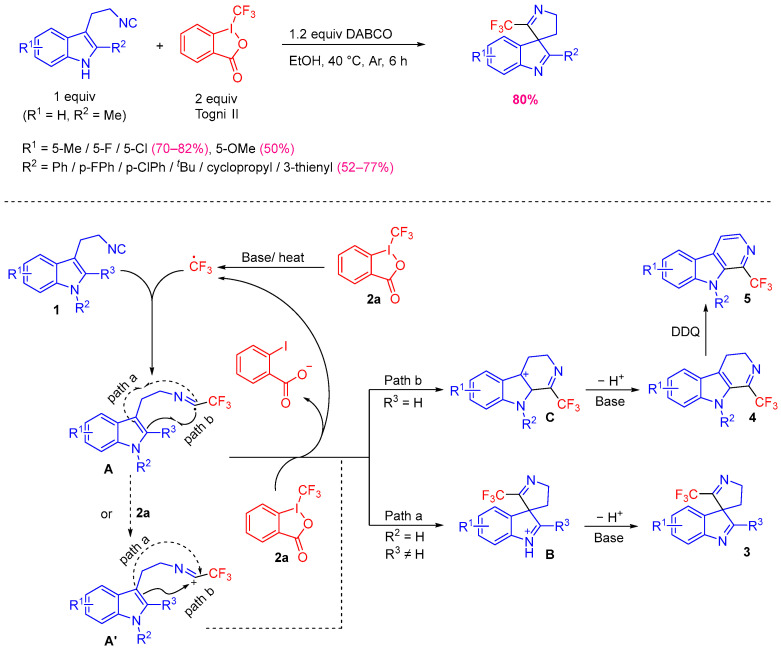
Selective synthesis of CF_3_-substituted *β*-aza-spiroindolenines.

Yuan et al. reported a metal-free radical dearomative annulation of C2-substituted tryptamine-derived isocyanides with Togni II giving CF_3_-substituted *β*-aza-spiroindolenines (Figure 45; Table S41). With DABCO in EtOH at 40 °C, a C2-methyl isocyanide gave 80%; the C2 substituent is essential, as the C2-H substrates divert to *β*-carbolines. C2 aryl/alkyl groups gave 52–77% and indole-ring halides/methyl 70–82% (OMe 50%). NaBH_4_ reduction gave the corresponding spiroindoline (70%). Mechanism includes CF_3_ radical (generated from Togni II) addition to the isocyanide forming an imidoyl radical, C3-selective annulation (favored by C2 sterics), then deprotonation [61].

**Figure 46:**
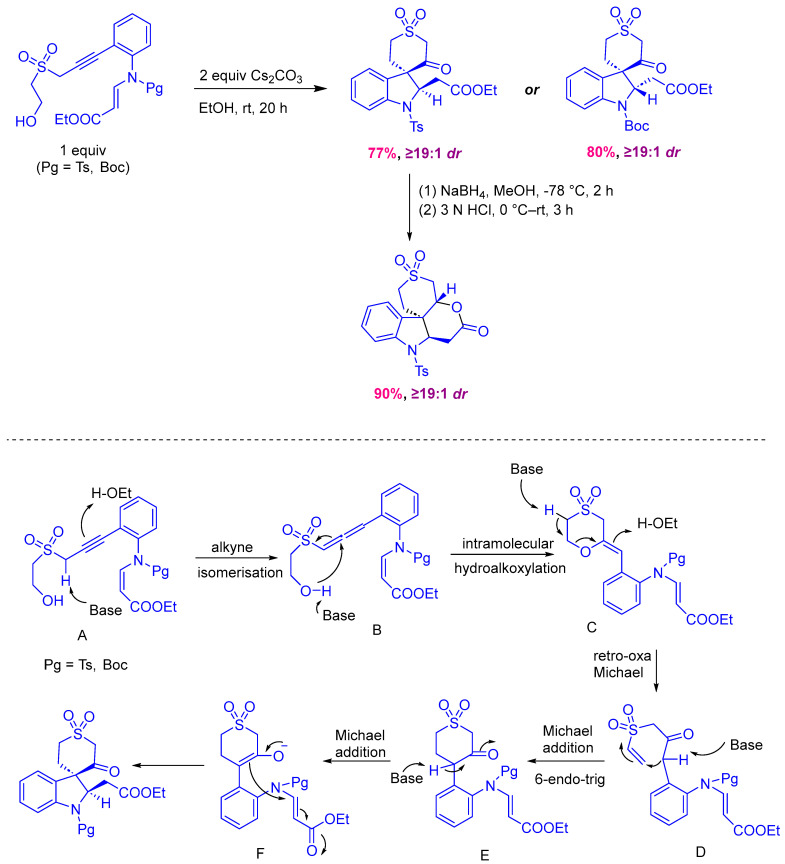
Cascade spirocyclization of propargyl sulfones.

Gharpure et al. reported a base-promoted cascade (propargyl-sulfone to allene isomerization, hydroalkoxylation, retro-oxa-Michael, 6-endo-trig Michael) that delivers tricyclic *β*-ketosulfone spiroindolines (Figure 46). With Cs_2_CO_3_ in EtOH, sulfone-tethered alkynyl vinylogous carbamates gave spiroindolines in 77–80% with ≥19:1 *dr* (*N*-Ts vs. *N*-Boc giving different major diastereomers). Subsequent reduction in the *N*-Ts adduct with NaBH_4_ followed by HCl addition produced the corresponding tetracyclic spiroindoline (90%, ≥19:1 *dr*) [62].

**Figure 47:**
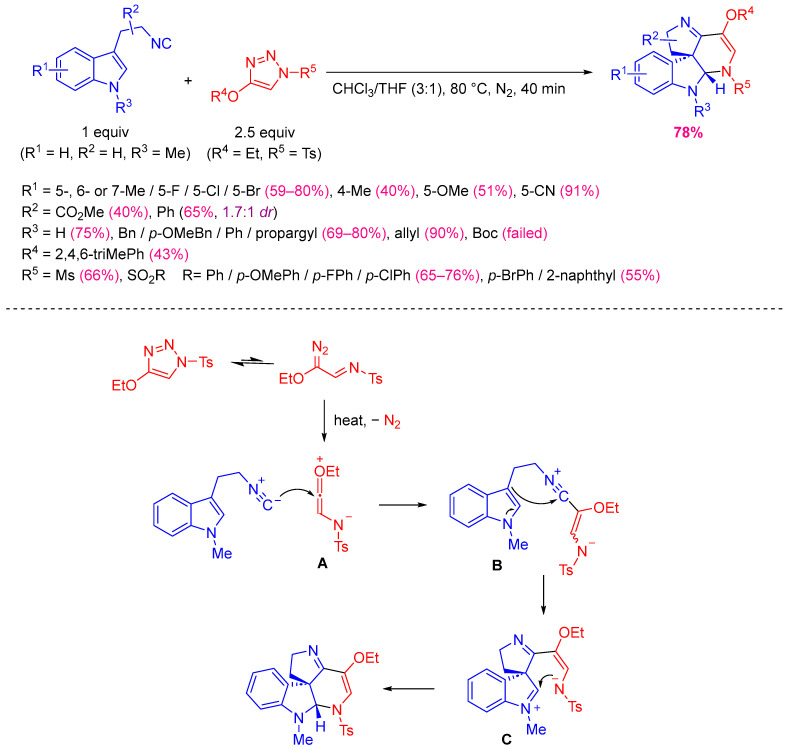
Catalyst-free spirocyclization with 1-sulfonyl-1,2,3-triazoles.

Chen et al. reported a catalyst-free spirocyclization of 3-(2-isocyanoethyl)indoles with 1-sulfonyl-1,2,3-triazoles giving *α*-carboline-containing [6,5,5,6] tetracyclic spiroindolines (Figure 47; Table S42). In CHCl_3_/THF (3:1) at 80 °C, a 4-ethoxy triazole gave 78%; rhodium completely failed to catalyze the reaction. *N*-H and *N*-substituted indoles were tolerated (69–90%), while *N*-Boc failed; indole ring C5-substitution depicted some electronic preference (5-CN 91% vs. 5-OMe 51%). Alkoxy/aryloxy group on the triazole was needed; a simple phenyl-triazole failed. Gram-scaling was satisfactory and products could undergo subsequent reduction and desulfonylation. Mechanism involves a thermal intermediate derived from triazole reacting with the isocyanide, intramolecular C3 attack, then N addition to the iminium [63].

**Figure 48:**
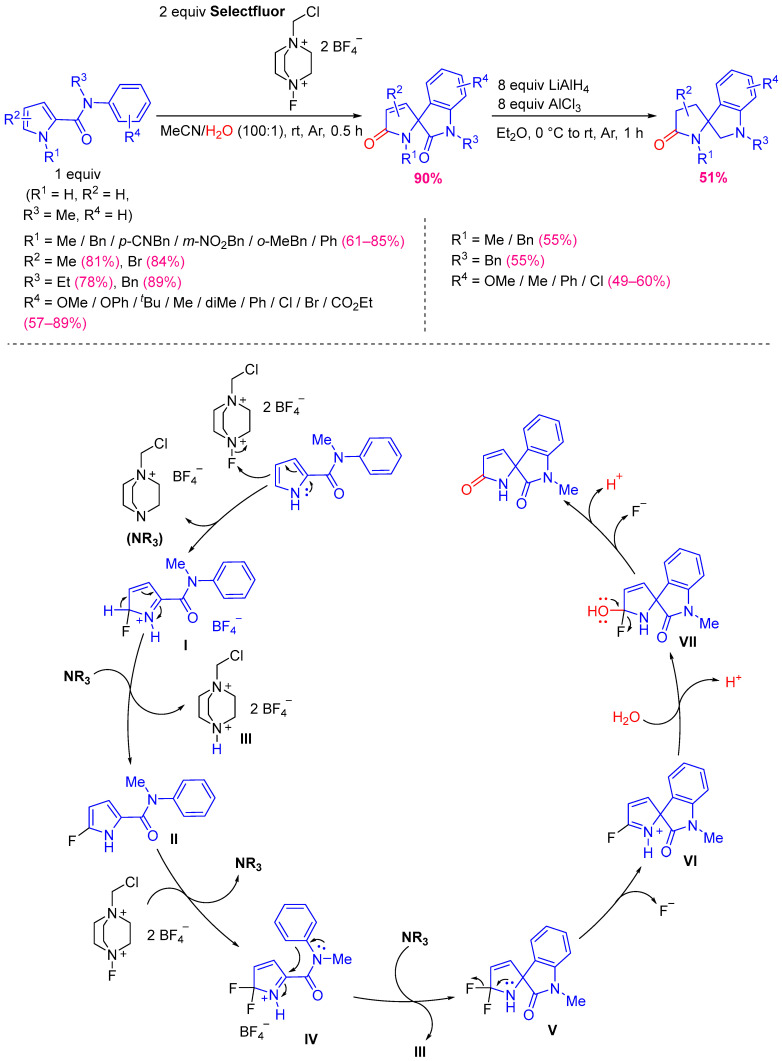
Metal-free oxidative spirocyclization with Selectfluor.

Zhao et al. reported a metal-free Selectfluor-mediated oxidative spirocyclization of pyrrole-2-carboxamides to 3,3′-pyrrolidinyl spirooxindoles, followed by reduction to spiroindolines (Figure 48; Table S43). With Selectfluor in MeCN/H_2_O (100:1), spirooxindoles formed in up to 90%; LiAlH_4_/AlCl_3_ reduction then gave spiroindolines (~50%), while Pd/C selectively reduced only the pyrrole’s C=C (87%). Gram-scaling achieved 70% spirooxindoles. Isotope labeling confirmed water as the O source and deuterium competition reactions excluded C-H cleavage from the rate-determining step. Mechanism involves pyrrole attack on Selectfluor, amine-base deprotonation, further fluorination, intramolecular addition, and then formal hydrolysis [64].

**Figure 49:**
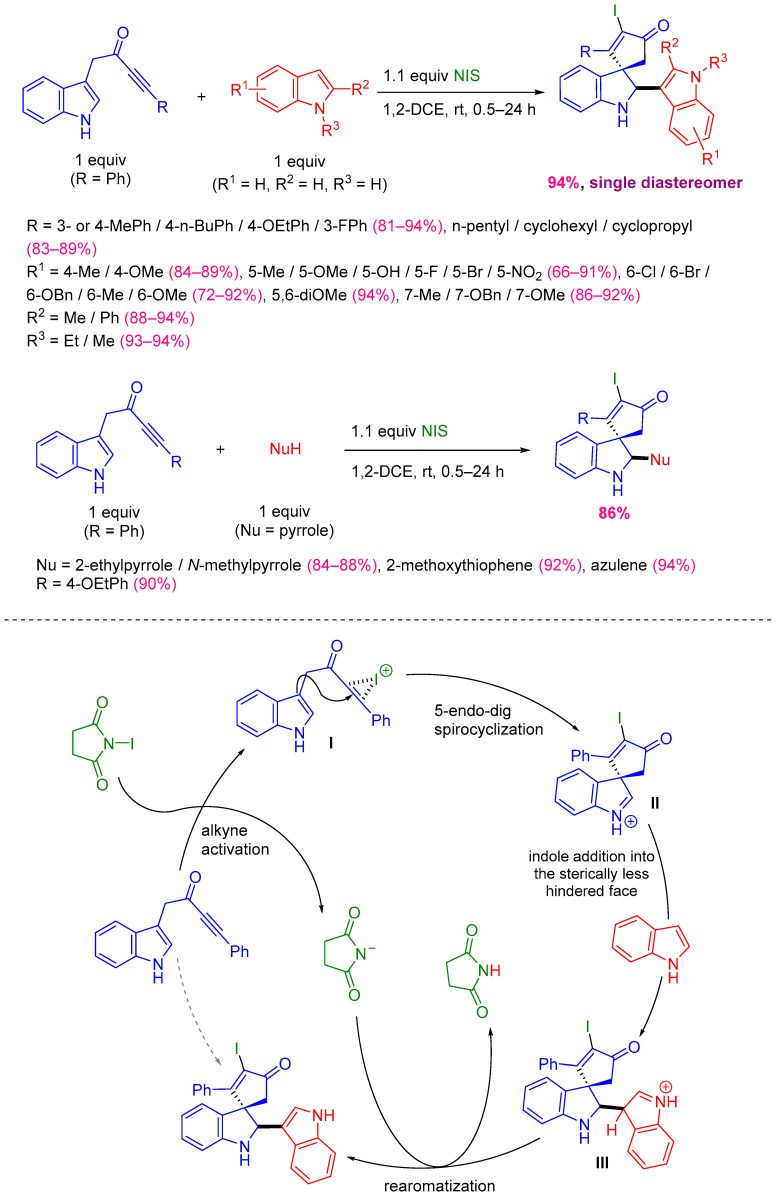
Iodocyclization/nucleophile addition cascade.

Bag et al. reported an NIS-mediated *ipso*-iodocyclization/nucleophile-addition cascade of indole-tethered ynones giving polyfunctionalized spiroindolines bearing two stereocenters, a new C-I bond and two new C-C bonds (Figure 49; Table S44). With NIS in 1,2-DCE, products formed in up to 94% as single diastereomers in all cases. Indole nucleophiles bearing 4–7 substituents gave 66–94%; *N*-tosyl and 3-substituted indoles failed, and varied ynone termini gave 81–94%. Pyrroles, methoxythiophene and azulene were also competent as nucleophiles (up to 94%). Mechanistically, NIS-generated iodonium triggers 5-endo-dig *ipso*-cyclization to a spiroindolenium, then face-selective nucleophile addition/rearomatization delivers the single diastereomer [65].

**Figure 50:**
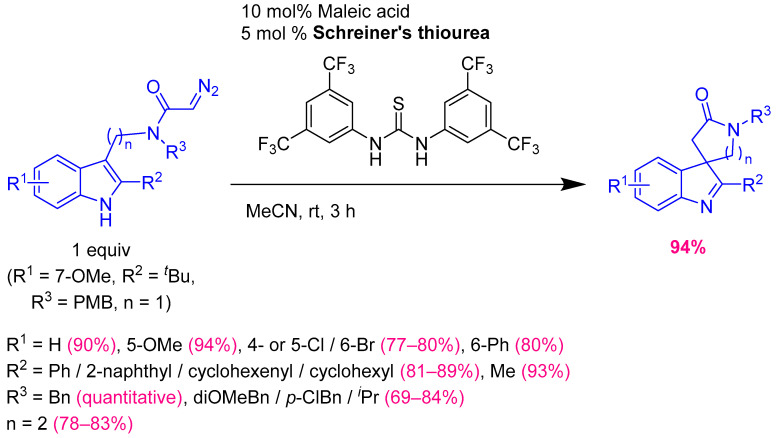
Dearomative *ipso*-Friedel–Crafts via cooperative acid/thiourea catalysis.

Ueda et al. reported a metal-free dearomative *ipso*-Friedel-Crafts of diazo-functionalized indoles under cooperative maleic acid/thiourea catalysis, giving spiroindolenines even with tertiary-alkyl C2 substitution otherwise hard to access (Figure 50; Table S45). In MeCN, yields reached 94%; cooperative catalysis outperformed either catalyst alone (Rh failed; Ag poor). C2-methyl substrate gave 93% under optimal conditions (vs. Rh catalysis: 0%; Ag catalysis: 60%). Indole-ring substituents (77–94%), varied C2 groups (81–93%), and amide *N*-substituents were tolerated. Both five- and six-membered spirocycles were formed. Chiral thioureas gave near-racemic products (0–2% *ee*) [66].

**Figure 51:**
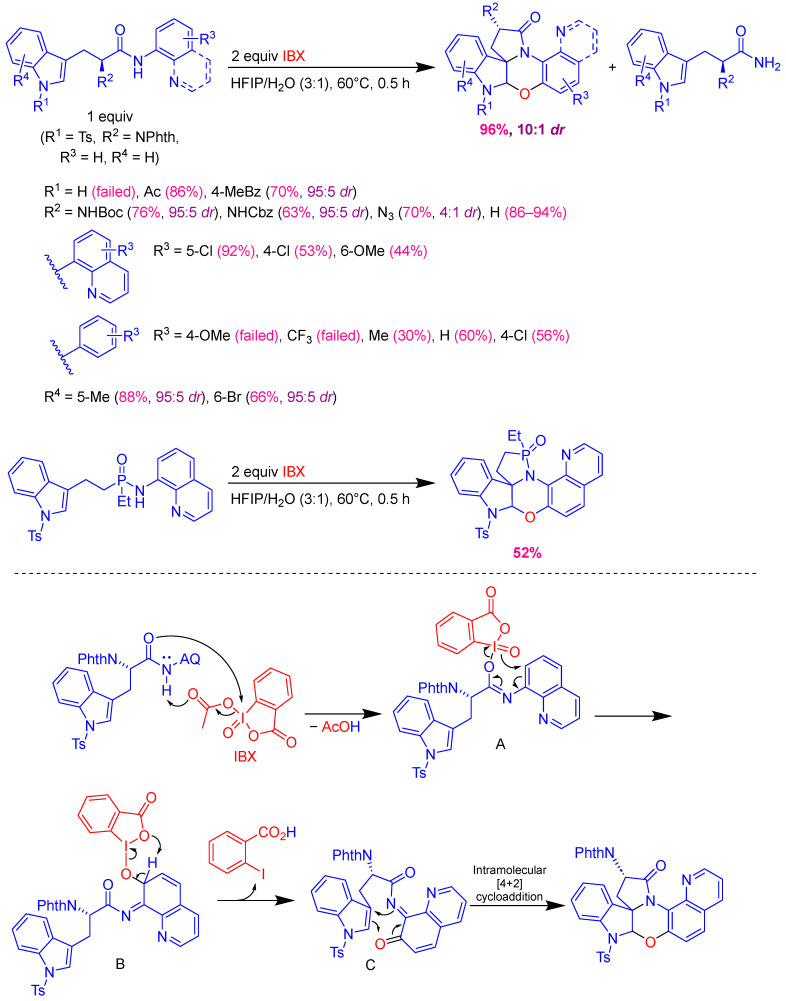
2-Iodoxybenzoic acid (IBX)-mediated intramolecular oxidative cyclization.

Zhang et al. reported a 2-iodoxybenzoic acid (IBX)-mediated intramolecular oxidative cyclization engaging both C2 and C3 of the indole, building polycyclic spiroindolines with multiple (including quaternary) stereocenters, and exceptionally reporting the first azaphospholidine-containing spiroindoline (Figure 51; Table S46). With IBX in HFIP/H_2_O (3:1) at 60 °C, yields reached 96%; combining both solvent components was crucial, reaction failed in either solvent alone. Indole *N*-protection was required (*N*-H failed). Mechanism involves IBX activating the amide oxygen to an iodoimidate, dearomatization to an *o*-iminoquinone, then inverse-electron-demand hetero-Diels-Alder with the alkene [67].

**Figure 52:**
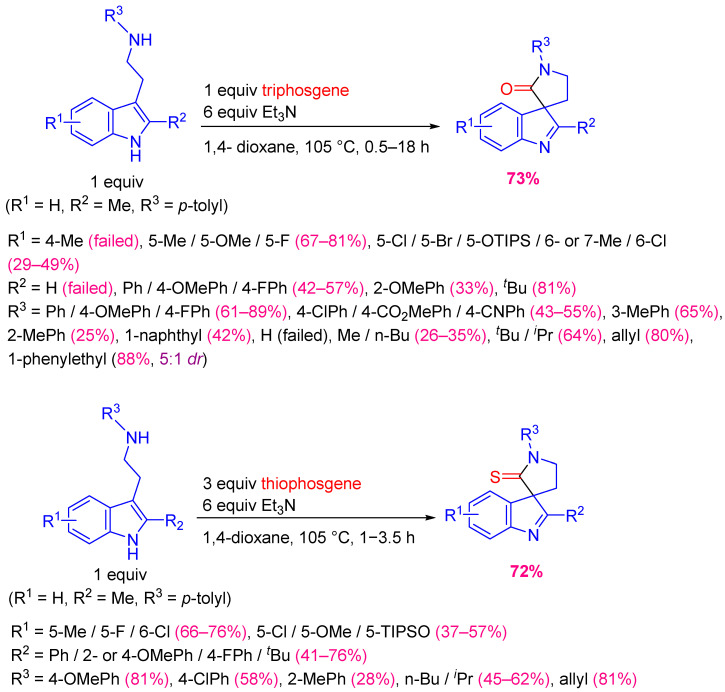
(Thio)chloroformylation/dearomative spirocyclization of tryptamines.

Yasui et al. reported a one-pot (thio)chloroformylation/dearomative spirocyclization of tryptamines giving spiro[indole-3,3′-pyrrolidine]-2′-ones and, for the first time, the 2′-thiones (Figure 52; Table S47). With triphosgene or thiophosgene/Et_3_N in refluxing dioxane, (thio)carbamoyl chloride intermediates cyclize to products in 25–89%; Et_3_N markedly improved efficiency. A chiral amine was tolerated (5:1 *dr*, X-ray confirmed), while C2-H substrates stalled at the carbamoyl chloride. Thiophosgene gave the more reactive thiocarbamoyl chlorides, delivering spirothiolactams up to 81% [68].

**Figure 53:**
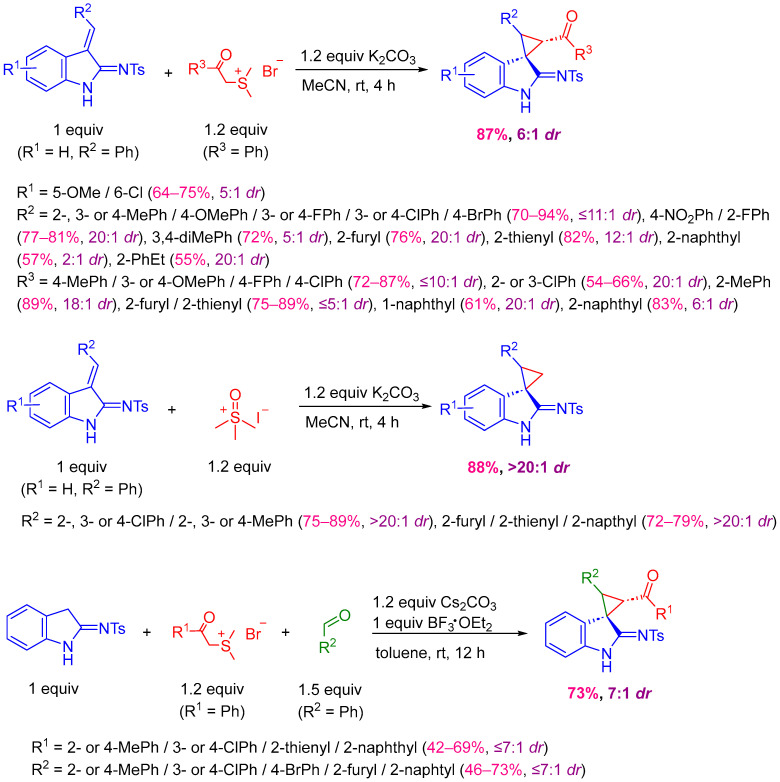
Sulfur ylide-mediated (2 + 1) annulation and a three-component (1 + 1 + 1) variant.

Dai et al. reported a sulfur ylide-mediated formal (2 + 1) annulation (and a three-component (1 + 1 + 1) variant) giving spiro-cyclopropyl iminoindolines (Figure 53; Table S48). Aza-dienes with sulfonium bromides or Corey-Chaykovsky reagent and K_2_CO_3_ in MeCN gave 54–94% (2:1–20:1 *dr*); aryl aza-dienes outperformed alkyl (20:1 *dr*). Sulfonium bromides with halogen/methyl/methoxy-substituted phenyl gave 54–89%. In the three-component cascade, aza-dienes were generated in situ by Knoevenagel condensation in the presence of a Lewis acid catalyst; giving 42–73% (≤7:1 *dr*). Products can undergo BF_3_·OEt_2_ ring-opening rearrangement followed by oxidation/rearrangement with 3,3′,5,5′-tetra-tert-butyldiphenoquinone to give pyrrolo[3,4-*c*]quinolin-1-ones (42–53%). Gram-scaling of the (2 + 1) annulation was successful (90%, 9:1 *dr*) and an organocatalytic tetrahydrothiophene trial was feasible but less selective (62%, 1:1 *dr*) [69].

**Figure 54:**
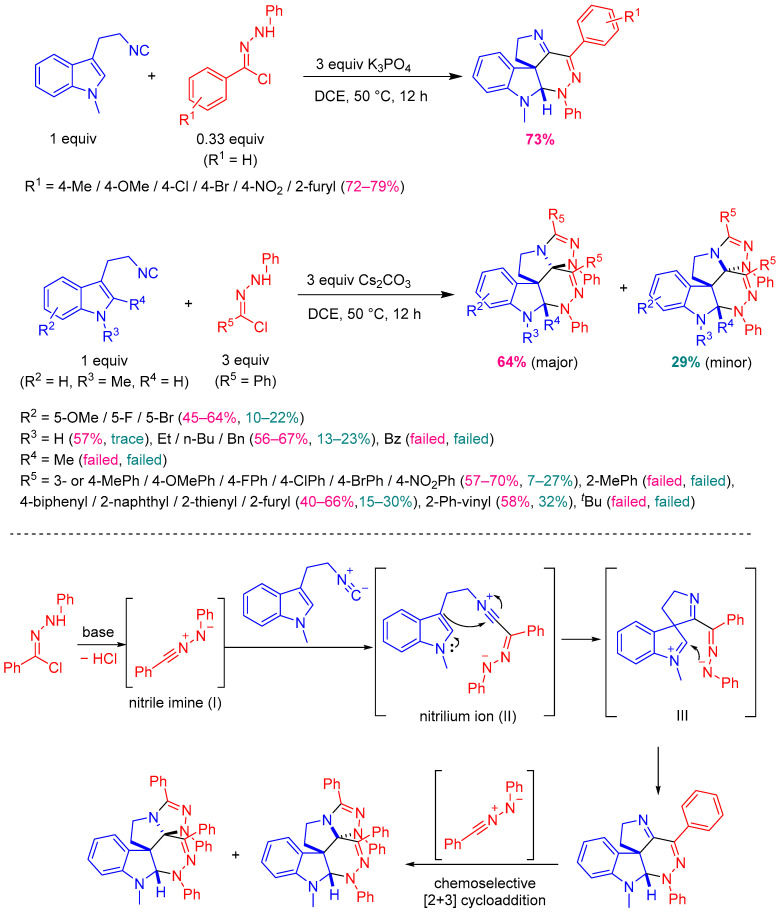
Base-controlled [1 + 2 + 3] and [1 + 2 + 3]/[2 + 3] annulations with hydrazonyl chlorides to tetracyclic spiroindolines (**upper**) and pentacyclic bispiroindolines (**lower**).

Wang et al. reported a base-controlled annulation of tryptamine-derived isocyanides with hydrazonyl chlorides, where different bases and stoichiometry dictate [1 + 2 + 3] tetracyclic spiroindolines versus continuation to [1 + 2 + 3]/[2 + 3] pentacyclic bispiroindolines (Figure 54; Table S49). K_3_PO_4_ with excess isocyanide (3:1) gave tetracycles in 72–79%, while Cs_2_CO_3_ (1:3) gave bispiroindolines as two diastereomers. Both manifolds tolerated broad hydrazonyl-chloride aryls/heteroaryls. With Cs_2_CO_3_, *N*-benzoyl and 2-methylindole as well as *ortho*-substituted aryl and *tert*-butyl hydrazonyl chlorides failed. The reaction scaled to gram quantity (bispiroindoline: major 61%, minor 20%). Mechanism includes in situ nitrile imine formation, isocyanide attack to a nitrilium, annulation to tetracycles, then chemoselective [2 + 3] cycloaddition to pentacycles [70].

**Figure 55:**
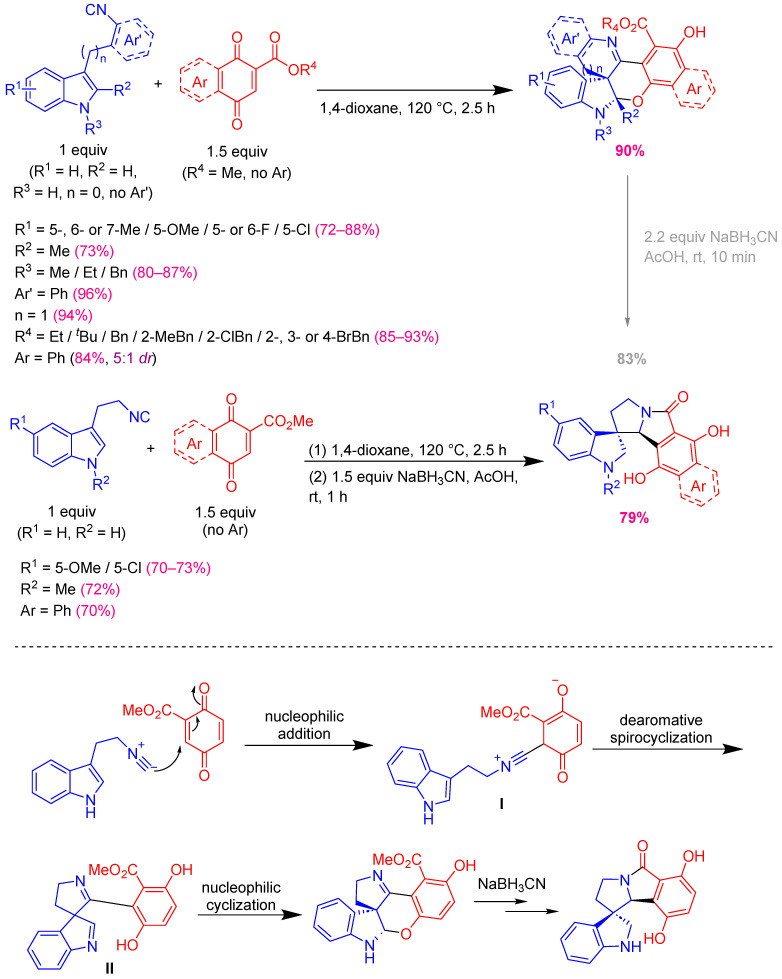
Catalyst-free dearomative spirocyclization with quinone esters to chromeno[2,3-*b*]indole followed by reduction to spiroindoline (**upper**) or through a one-pot strategy (**lower**).

Wang et al. reported a catalyst-free dearomative spirocyclization of tryptamine-derived isocyanides with quinone esters giving chromeno[2,3-*b*]indoles, reducible to polycyclic spiroindolines or directly through a one-pot synthesis (Figure 55; Table S50). In dioxane at 120 °C, the chromenoindole formed in 90%. Indole substituents (incl. 2-methyl, 73%), *N*-alkyl groups and alkyl spacer extension or phenyl incorporation gave 72–96%, while *N*-Boc/Ts failed; the quinone ester group was essential. A tryptophanate-derived isocyanide also gave chromenoindole (79%, 1:1 *dr*) and naphthoquinone derivatives were successful (73–84%). NaBH_3_CN/AcOH then triggers a cascade to the corresponding spiroindoline (83%). One-pot strategy was feasible for direct polycyclic spiroindolines synthesis (70–79%). Mechanism includes isocyanide attack on the quinone to a nitrilium, dearomative spirocyclization, intramolecular addition to the chromenoindole, and then reductive C=N/lactamization and dihydropyran opening [71].

**Figure 56:**
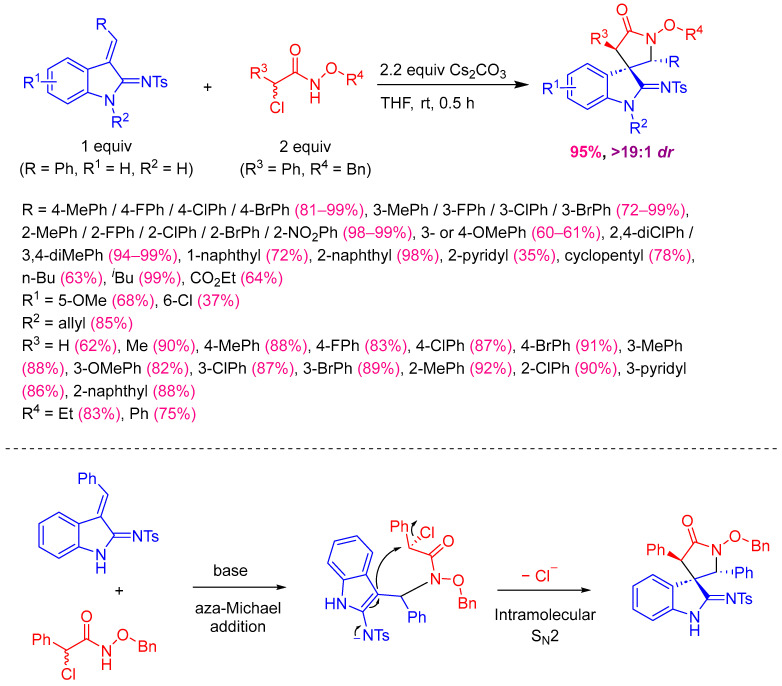
Base-promoted formal (3 + 2) cycloaddition with *α*-halohydroxamates.

Zhang et al. reported a base-promoted formal (3 + 2) cycloaddition of *α*-halohydroxamates with alkenyl-iminoindolines giving spiro-indolinepyrrolidinones, exploiting the alkene (not the imine) as the reactive site (Figure 56; Table S51). With Cs_2_CO_3_ in THF, the model gave 95% and >19:1 *dr* which was commonly present across synthesized derivatives. Aza-dienes with diverse aryl/heteroaryl/alkyl/ester groups gave 35–99%, and *α*-chlorohydroxamate variation gave 62–92%. Gram-scale (1.2 g; 73%, >19:1 *dr*) and many derivatizations were conducted including thiolactam formation with Lawesson’s reagent (52%, >19:1 *dr*), amido carbonyl removal with DIBAL-H in DCM at room temperature (42%, >19:1 *dr*) and over-reduction in the carbonyl and imino groups with DIBAL-H and PhCF_3_ at increased temperature (51%, >19:1 *dr*). A chiral substrate gave product with retained *ee*, supporting stereospecific S_N_2. Mechanism involves deprotonation, aza-Michael addition, and then intramolecular S_N_2 C-C bond formation [72].

#### 3.2.2. Photocatalytic/Visible-Light Methods

Photocatalytic strategies have emerged as powerful tools for spiroindoline synthesis, enabling mild conditions, selectivity, and access to radical intermediates that are difficult to generate thermally. The following methodologies employ visible-light photocatalysts, UV irradiation, or photoredox mechanisms.

**Figure 57:**
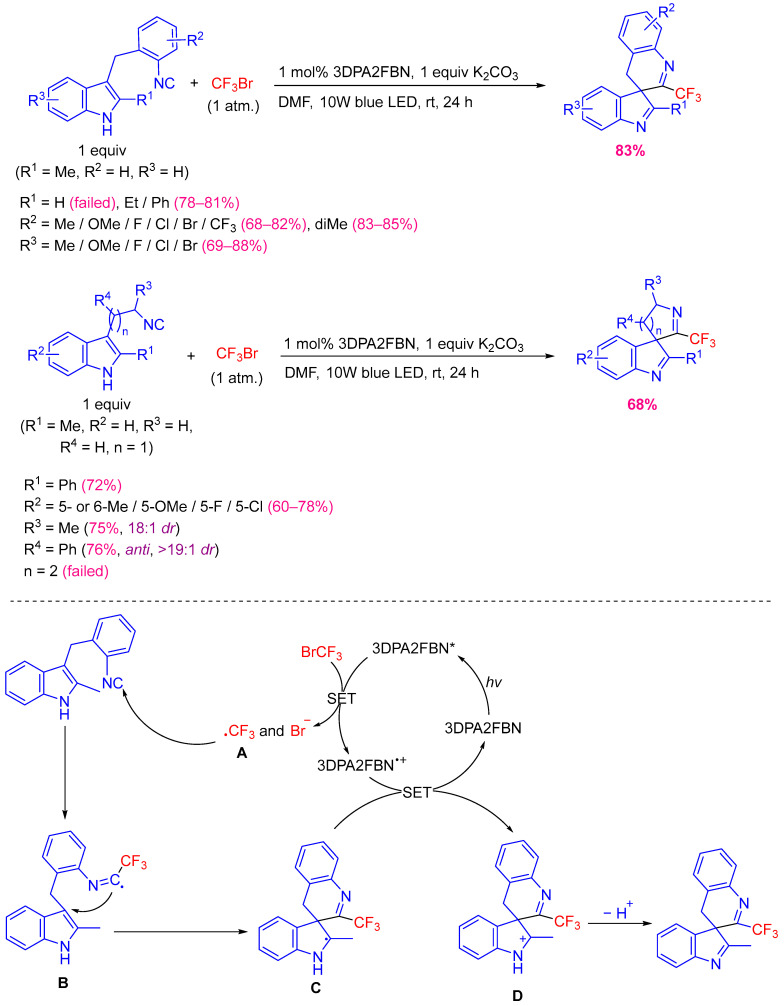
Visible-light radical trifluoromethylation/cyclization/dearomatization cascade using CF_3_Br.

Lv et al. reported a visible-light radical trifluoromethylation/cyclization/dearomatization of isocyanide-containing indoles using CF_3_Br, giving CF_3_-spiro[indole-3,3-quinoline] and -pyrrole derivatives (Figure 57; Table S52). With 2,4,6-tris(diphenylamino)-3,5-difluorobenzonitrile photocatalyst (3DPA2FBN)/K_2_CO_3_ in DMF under blue LED, yields reached 88%; photocatalyst, base and light were all essential. Indole C2-substitution was required, where C2-H diverted to 3-substituted indole (76%) instead of the desired spiroindoline. Both substrate classes tolerated broad substitution, giving high *dr* in some cases. Reduction in spiroindolines using NaBH_4_ selectively reduced the C=N bond attached to CF_3_ (84%) and using LiAlH_4_ reduced both C=N bonds (64%); selective hydrolysis/ring-opening (96%) and gram-scaling (79%) were also successful. Mechanistic studies (TEMPO trapping, on/off, quenching) confirmed a CF_3_ radical that adds to the indole, cyclizes, then oxidizes/deprotonates to the spiroindoline [73].

**Figure 58:**
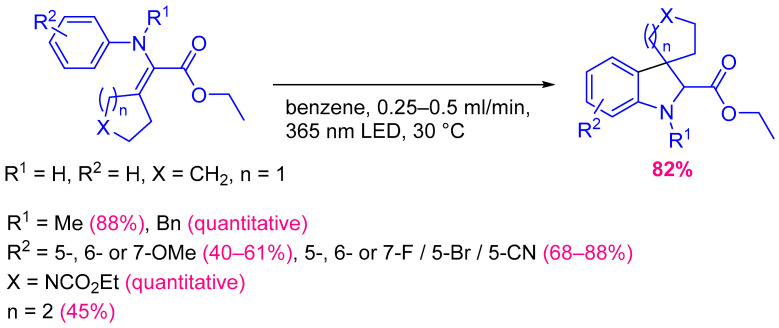
UV-light photocyclization in continuous flow.

Moustakim et al. translated a UV-photocyclization of aryl-enamine into continuous flow conditions; gaining heat control, consistent light penetration, controlled exposure time and inherent scalability (Figure 58; Table S53). In benzene under a 365 nm UV-A LED at 30 °C (0.25–0.5 mL/min), spiroindolines formed in up to quantitative yields with greatly reduced reaction times versus batch. EDG/EWG aryl-enamines gave 40–88% and heteroatom-containing spirocycle, which failed in batch, succeeded in flow with quantitative yield, expanding the scope. Theoretical output of this protocol under steady state conditions exceeded 18 g/day. The method enabled a shortened formal synthesis of (±)-horsfiline, including the first mild UV-A oxidative decarboxylation to a spirooxindole [74].

**Figure 59:**
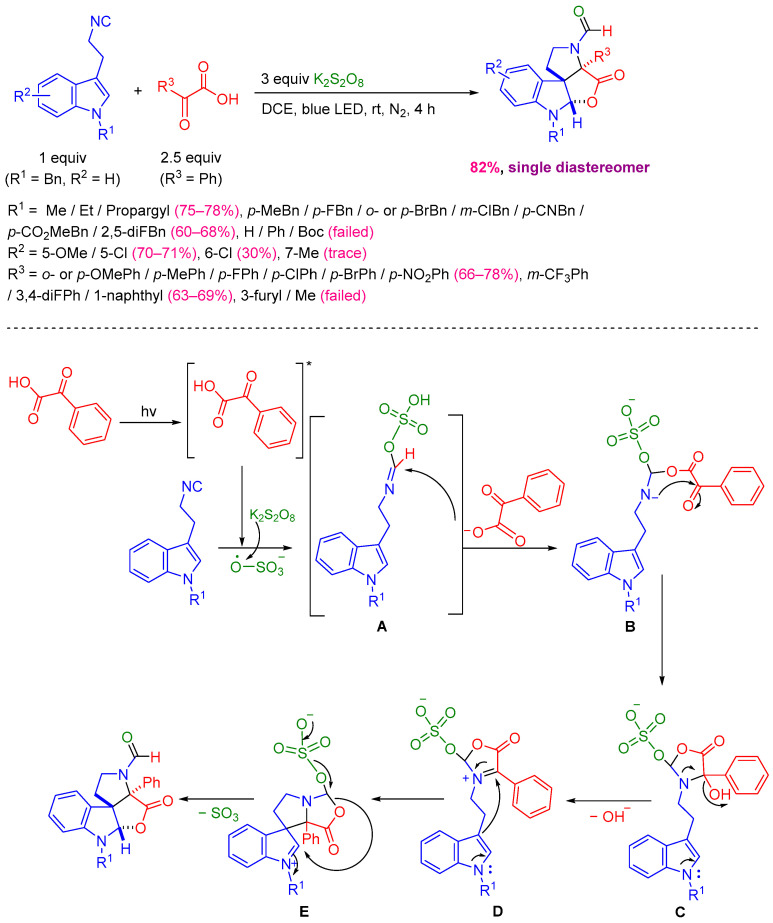
Visible-light/K_2_S_2_O_8_-mediated cascade cyclization with *α*-oxocarboxylic acids.

Ranga Rao et al. reported a visible-light/K_2_S_2_O_8_ cascade cyclization of 3-(2-isocyanoethyl)indoles with *α*-oxocarboxylic acids giving *N*-formyl [6,5,5,5] tetracyclic spiroindolines, with glyoxylate ions involvement without decarboxylation (Figure 59; Table S54). In DCE under blue LED (N_2_), products formed in up to 82%, mostly as single diastereomers. Aromatic *α*-oxocarboxylic acids gave 63–78% (methyl/furyl failed), and diverse indole/*N*-substituents gave 60–78% (6-Cl 30%), though *N*-H/*N*-Boc/*N*-phenyl and C7-substitution failed. A flow variant of the model reaction gave 82% in only 2.48 min residence time. Labeling shows the formyl H originates from the acid. Mechanism involves photochemical glyoxylate generation, indole engagement, dearomative cyclization to a nitrilium, then C2 attack/SO_3_ loss [75].

**Figure 60:**
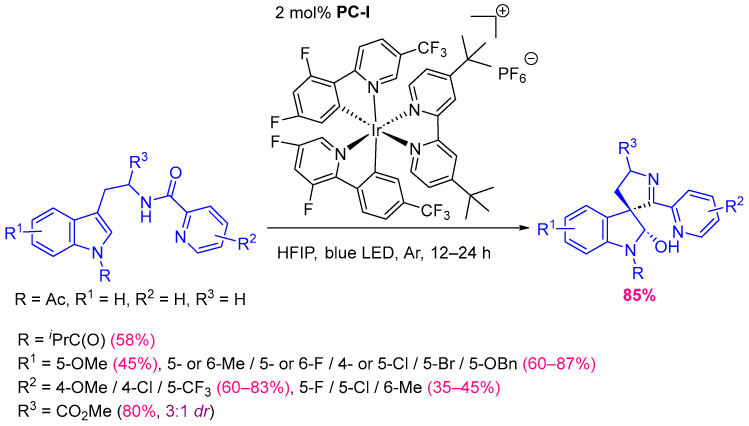
Visible-light energy transfer/triplet excited state intermolecular proton transfer (T-ESPT) dearomative spirocyclization.

Yang et al. reported a visible-light energy-transfer dearomative spirocyclization of amide- and pyridine-bearing indoles giving hydroxy-substituted spiroindolines (35–87%, >20:1 *dr*) (Figure 60; Table S55). With photocatalyst PC-I in HFIP under blue LED, the model gave 85%; the pyridine-HFIP interaction drives a triplet excited state intermolecular proton transfer (T-ESPT) that activates the otherwise unreactive amide. HFIP was uniquely effective where other solvents gave no product; photocatalyst and visible-light were also essential. Indole and pyridine substituents were broadly tolerated, while benzoic-acid-derived substrates failed. DFT/quenching studies support energy transfer (not a single electron transfer): triplet excitation, HFIP-assisted T-ESPT, then intramolecular cyclization [76].

#### 3.2.3. Organocatalytic Methods

**Figure 61:**
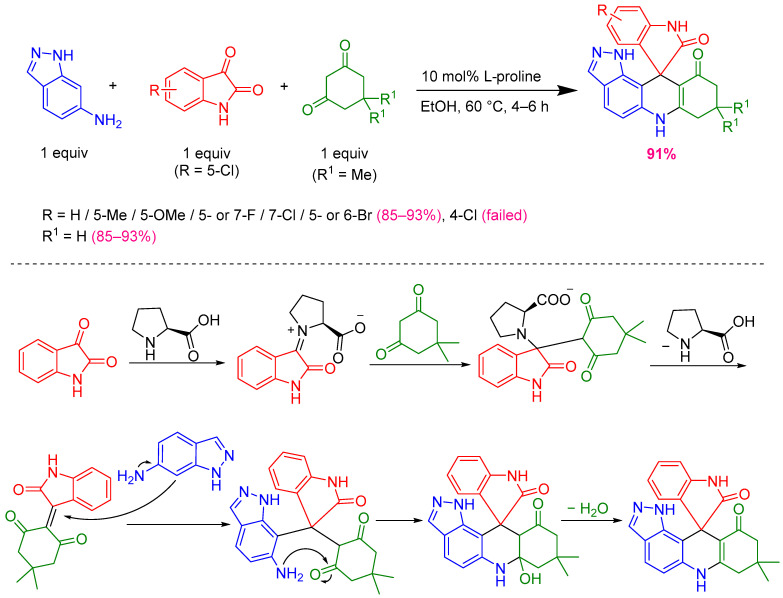
L-proline-catalyzed three-component condensation.

Wang et al. reported a green L-proline-catalyzed one-pot three-component condensation of 1*H*-indazol-6-amine, isatins and cyclohexane-1,3-diones giving spiro[indoline-3,11′-pyrazolo[3,4-*a*]acridine]-2,10′(1′*H*)-diones (Figure 61; Table S56). L-Proline in EtOH at 60 °C gave the model in 91%; no catalyst gave no reaction. Isatins bearing EWG/EDG and both dimethyl and unsubstituted diketones were tolerated (85–93%), while 4-chloroisatin failed due to sterics. Mechanism includes proline-mediated iminium formation with isatin, Knoevenagel with the 1,3-dione, Michael addition of the amine, then cyclization/dehydration [77].

**Figure 62:**
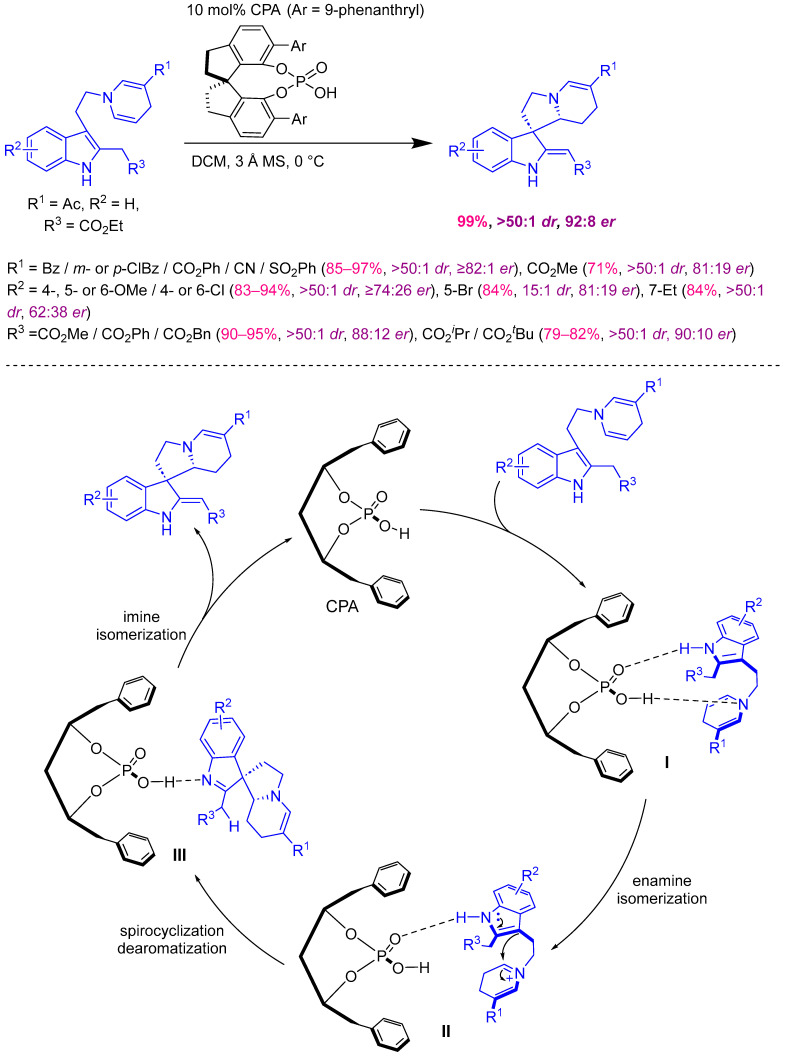
Chiral phosphoric acid (CPA)-catalyzed enamine isomerization/spirocyclization/dearomatization.

Pan et al. reported a SPINOL-CPA-catalyzed asymmetric enamine isomerization/spirocyclization/dearomatization of tryptamine-derived indolyl dihydropyridines giving 2,7-diazaspiro[4.4]nonane spiroindolines (Figure 62; Table S57). With CPA/3 Å MS in DCM at 0 °C, the model gave 99% with >50:1 *dr* and 92:8 *er*. Substituted dihydropyridines gave high yields with high diastereoselectivity and enantioselectivity. Indole substituents gave 83–94%; essential C2 esters gave 79–95%; bulky esters lower. Mechanism involves CPA H-bond activation to an enamine, CPA (as Brønsted-acid) isomerization to an iminium, enantioselective C3 spirocyclization, and then dearomatization [78].

**Figure 63:**
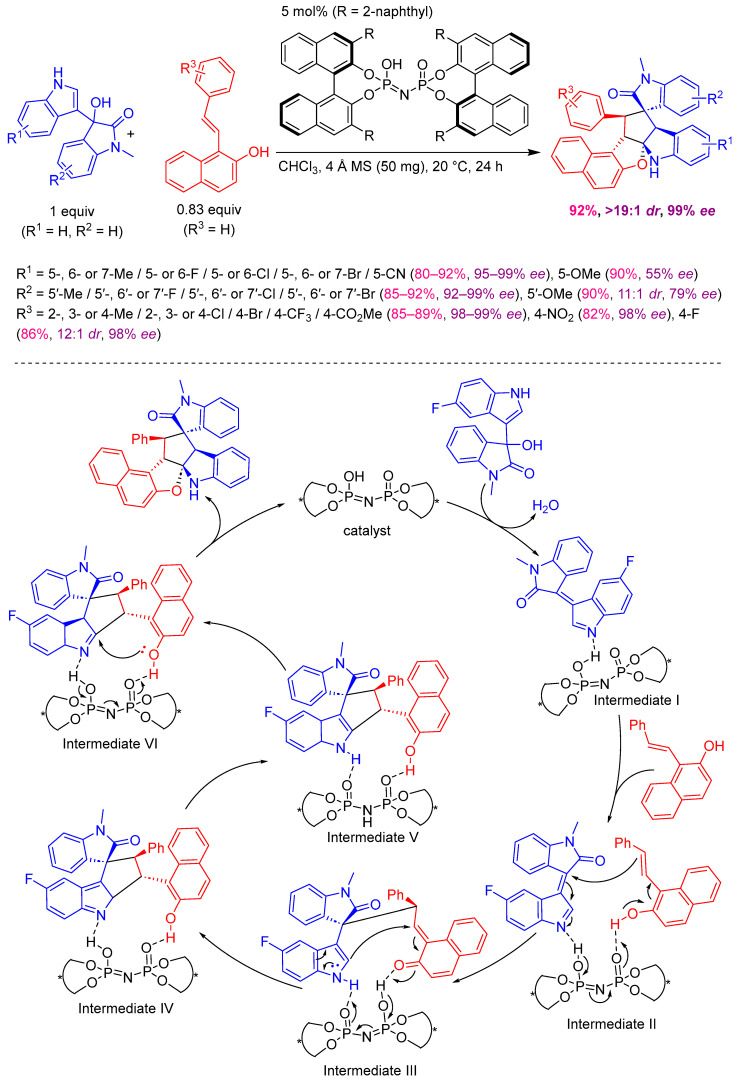
BINOL-type chiral imidodiphosphoric acid catalyzed [3 + 2]/spirocyclization. The asterisk (*) indicates the omitted remainder of the CPA catalyst depicted in the reaction scheme.

Zhang et al. reported a BINOL-derived chiral imidodiphosphoric-acid-catalyzed consecutive [3 + 2]/spirocyclization building spiro[benzofuro-cyclopenta[1,2-*b*]indole-indoline] scaffolds with five contiguous stereocenters and two spiroheterocycles (Figure 63; Table S58). With CPA/4 Å MS in CHCl_3_ at 20 °C, isatin-derived 3-indolylmethanol and (*E*)-1-styrylnaphthalen-2-ol model gave 92% with >19:1 *dr* and 99% *ee*. Both the indole *N*-H and the naphthol *O*-H were essential (*N*-Me low yield and racemic, *O*-Me unreactive). Scope was broad, with EDG as 5-OMe on indole eroding *ee*. Mechanism includes vinyl-iminium formation, *Si* face 1,4-addition generating an *o*-QM, [3 + 2] cycloaddition, then *Re* side intramolecular spirocyclization [79].

Metal-free, photocatalytic, and organocatalytic methods reported for the synthesis of C3-spiroindolines are summarized in Table 3, highlighting the diversity of activation modes, substrate classes, reaction efficiencies, and stereoselectivities achieved.

A recurring theme across the C3-spirocyclization literature is the central role of 3-(2-isocyanoethyl)indoles and closely related tryptamine-derived isocyanides as a unifying substrate platform. The diversity of products accessible from this single building block, depending on catalyst and coupling partner, is summarized in Table 4.

## 4. Conclusions and Future Prospects

Spiroindoline synthesis has advanced substantially since the landmark 2018 review by Bariwal, Voskressensky, and Van der Eycken emphasized that dearomative indole intermediates often tend to re-aromatize, thereby limiting the efficient formation of spiroindoline frameworks [17]. The methods surveyed in the present review show that this challenge has been substantially mitigated, although not eliminated. Rh(III)-catalyzed C-H activation/dearomative annulation, Pd-catalyzed dearomative annulation and cascade processes, copper-mediated cascade chemistry, and metal-free organocatalytic approaches now provide reliable pathways to intercept dearomative intermediates before rearomatization can occur. As a result, access to spiroindoline scaffolds has become broader, more stereochemically controlled, and more adaptable to structurally diverse substrates.

At the C2 position, developments between 2020 and 2025 are most notable for improved regioselectivity and stereochemical control in a position that has traditionally been less reactive than C3. Rh(III)- and Ru(II)-catalyzed C-H activation/annulation strategies have expanded direct access to C2-spiroindolines, while palladium catalysis has enabled asymmetric dearomatization and tandem rearrangement processes. Copper-based methods have further broadened the range of electrophilic and oxidative cascade partners. The strongest asymmetric outcomes in this section are observed in selected palladium-catalyzed and organocatalytic examples, especially chiral Brønsted acid platforms, which demonstrate that high enantioselectivity at C2 is achievable when substrate design and catalyst environment are carefully matched.

At C3, the field is broader and mechanistically more diverse. The variety of catalytic manifolds operating at this position, including rhodium, palladium, copper, gold, silver, manganese, yttrium, bismuth, Lewis acids, organocatalysts, and photoredox systems, reflects the higher intrinsic reactivity and geometric accessibility of C3. A recurring theme is the value of 3-(2-isocyanoethyl)indoles as versatile building blocks (Table 4). This substrate class has enabled several cascade spirocyclization strategies, including nitrilium/iminium-type, imidoylative, and ketenimine-based pathways, and continues to support new reaction designs for polycyclic spiroindoline synthesis [18].

Photocatalytic spirocyclization deserves a separate comment. The number of examples reported is modest, but the mechanistic logic is distinct enough from thermal catalysis to make even a small collection of reactions meaningful. Radical intermediates reach substrate sites that ionic pathways do not, and the mild conditions are genuinely attractive from a selectivity standpoint. A recent review by Das covering visible-light-induced dearomative annulation of indoles from 2019 to mid-2024 situates the spiro-specific chemistry within the wider landscape of photocatalytic indoline formation, including fused and angularly fused systems [80]. Alongside this work, these two accounts give a reasonably complete picture of where photocatalytic indole dearomatization currently stands. Whether this area fulfills its promise depends largely on whether enantioselective versions can be developed; so far, enantioselective photoredox spirocyclization of indoles remains comparatively underdeveloped.

Although most of the methods surveyed above are racemic or moderately diastereoselective, a growing subset of studies during the 2020–2025 period have achieved genuine enantioselective access to spiroindoline scaffolds. Several patterns are visible: CPA and bifunctional organocatalysis platforms dominate the highest enantioselectivity range, palladium-catalyzed dearomatizations now routinely deliver *er* values above 90:10 when ligand and substrate are carefully matched, and asymmetric induction at C2 is achievable but remains less frequently reported than at C3. Within the highest-performing organocatalytic regimes, the exceptional stereocontrol exhibited by CPAs are primarily driven by their ability to form highly organized, constrained chiral ion-pairs. By protonating transient indoleninium or iminium-type intermediates, CPAs create a rigid architectural pocket where the bulky substituents at the 3,3′-positions of the catalyst backbone effectively block one prochiral face, forcing the nucleophilic attack to proceed with exquisite facial selectivity. Concurrently, bifunctional platforms—particularly squaramide and thiourea-based catalysts—excel by managing dual activation pathways simultaneously. The parallel, highly acidic *N*–H protons of the squaramide core securely “clamp” the electrophilic component through precise, bidentate hydrogen bonding, while a tethered chiral tertiary amine pocket deprotonates and directs the incoming nucleophile. This cooperative, proximity-driven mechanism bypasses the need for precious transition metals, offering a highly predictable and robust alternative for setting the congested spiro-quaternary stereocenters of both C2- and C3-spiroindolines [81].

When contrasted with the mild, hydrogen-bonding manifolds of non-metal organocatalysis, the utilization of transition metal catalysis (Rh, Pd, Cu) for the construction of highly congested C2-quaternary spirocenters introduces several distinct organometallic complications. The primary challenge lies in managing competitive inner pathways; the formation of transient *σ*-alkyl-metal intermediates at the inherently crowded C2 position can trigger *β*-hydride elimination, diverting the reaction toward unwanted alkenes or premature rearomatization before spirocyclization can be completed. Forcing efficient reductive elimination or nucleophilic capture at this congested center often requires elevated temperatures, tailored ligands, or halide-scavenging silver additives to outcompete these pathways, frequently at some cost to chemo- and diastereoselectivity [82].

Moving forward, the next paradigm shift in spiroindoline construction must address the long-standing tension between structural complexity and experimental sustainability. While the synthetic toolkits developed between 2020 and 2025 have unlocked impressive structural diversity, many remain constrained by high catalyst loadings, precious transition metals, or tight substrate scope dependencies. A particularly promising frontier to solve these efficiency and selectivity challenges is the integration of concurrent or sequential enzyme-chemical catalysis (chemoenzymatic cascades). Combining the broad substrate tolerance of transition metal or organocatalyzed dearomatization with the exquisite, programmable stereoselectivity of evolved biocatalysts (such as imine reductases, monooxygenases, or halogenases) offers a dual advantage. This hybrid approach is uniquely positioned to solve two critical bottlenecks: first, the dynamic kinetic resolution of highly unstable, prone-to-rearomatize intermediates under ambient aqueous conditions, and second, the remote, late-stage functionalization of spiroindoline scaffolds that are otherwise sterically shielded from conventional chemical reagents [83].

The practical significance of these evolving strategies extends directly into industrial-scale medicinal chemistry and chemical biology. Spiroindoline frameworks represent privileged architectures in drug discovery, frequently surfacing in low-dose modulators of complex central nervous system targets and oncology pipelines. Transitioning from purely thermal or noble-metal-driven pathways to green, chemoenzymatic platforms reduces the heavy-metal contamination risks inherent to pharmaceutical manufacturing, substantially reduces the number of synthetic steps through one-pot cascade designs, and facilitates the rapid, sustainable generation of non-racemic spiroindoline libraries for high-throughput screening. Ultimately, bridging the gap between synthetic methodology and biocatalysis will transform spiroindolines from synthetically challenging targets into readily accessible, scalable building blocks for next-generation therapeutics.

The harder problem now is not selectivity but complexity. It is one thing to install a single quaternary spirocenter with high *ee*—recent methods do this convincingly. It is quite another to build several stereocenters in close proximity within a heavily substituted spiroindoline, and this remains a genuinely difficult task. Substrates that combine axial chirality with a spiro center, that place quaternary centers next to one another, or that approach the stereochemical density found in complex *Strychnos* and *Aspidosperma* alkaloids are still beyond what current catalytic methods reliably deliver. Progress here will almost certainly require new catalyst designs and more carefully engineered cascade sequences, not incremental refinement of existing platforms.

Data-driven reaction development is also largely absent from the spiroindoline literature surveyed here. Because spirocyclization outcomes are highly sensitive to substrate electronics, ligand structure, additives, solvent, and temperature, these reactions are well suited for high-throughput experimentation and machine-learning-assisted optimization. Such tools could help identify non-obvious conditions, improve stereoselectivity, and reduce reliance on extensive trial-and-error screening.

Overall, spiroindoline synthesis by the end of 2025 is methodologically rich, increasingly stereoselective, and clearly relevant to medicinal and natural product chemistry. The field now has many of the foundations needed for further progress, including established substrate classes, reliable catalytic platforms, stronger mechanistic understanding, and representative examples of downstream transformations. The next stage of development should focus on translation: from small-scale methodology to preparative and sustainable routes, from racemic or moderately selective reactions to broadly reliable asymmetric synthesis, and from isolated synthetic examples to libraries evaluated through meaningful biological and structure-activity studies. Progress over the 2020–2025 period provides a strong foundation for these goals.

## Figures and Tables

**Table 1:** Summary of catalytic methods for C2-spiroindoline synthesis.

Method (Lead Author, Figure)	Catalyst/Key Conditions	Substrates/Partners	Maximum Yield	Stereoselectivity
Yang (Figure 3)	Rh(III)/AgBF_4_, toluene, 60 °C, air	2-aryl-3*H*-indoles + cyclopropanols	up to 73%	-
Li (Figure 4)	[Cp*RhCl_2_]_2_, AgSbF_6_, AcOH, rt	2-aryl-3-nitrosoindoles + alkynes	up to 99%	>20:1 *rr*
Rupa & Anbarasan (Figure 5)	Rh_2_(OAc)_4_, PhMe, 120 °C	*o*-acylanilines + 3-diazoindoline-2-imines	up to 92%	-
Yu (Figure 6)	[Ru(*p*-cymene)Cl_2_]_2_, MeCN, 100 °C, Ar	alkenylanilines + diazopyrazolones	up to 91%	-
Zhang (Figure 7)	Pd(PPh_3_)_4_, LiO*^t^*Bu, LiCl, DMF, 60 °C	2-haloanilines + cyclic enones	up to 93%	high *dr*
Gao & Jiao (Figure 8)	Pd(dba)_2_, Carreira ligand, MeOLi, toluene, 90 °C	C2-arylindoles + internal alkynes	up to 99%	up to 95:5 *er*
Chang (Figure 9)	Pd_2_(dba)_3_, Xantphos, Et_3_B, K_2_CO_3_, DCE, 80 °C, N_2_	indol-2-yl-cyclobutanols + benzyl phosphates	up to 90%	>20:1 *dr*
Lei (Figure 10)	Cu(TFA)_2_, DCE, 40 °C, air	arylamines + styrylglyoxals	up to 94%	-
Panda & Ghorai (Figure 11)	TiPSY-CPA, MTBE, 4 Å MS, rt	aminochalcones + *α*-hydroxycyclobutanone	up to 78%	86–97% *ee*, >20:1 *dr*
Xie (Figure 12)	Et_3_N, CHCl_3_, rt, Ar	indolyl allylic alcohols + 2-tosyloxyketones	up to 89%	up to 13.0:1 *dr*
Sah (Figure 13)	quinine-amine, *α*-OHNA, DCM, rt	*γ*-arylated enone precursors	up to 99%	up to 97:3 *er*
Carceller-Ferrer (Figure 14)	bis-quinine squaramide, Na_2_CO_3_, CHCl_3_, 4 °C	4-Br-pyrazolones + aza-*o*-QMs	up to 68%	up to 95% *ee*
Zhao (Figure 15)	DBU, MeOH, rt	post-Ugi adducts	up to 99%	-
Dong (Figure 16)	Selectfluor (no catalyst), MeCN, rt, Ar	indole-2-carboxamides	up to 79%	-

**Table 2:** Summary of metal-catalyzed methods for C3-spiroindoline synthesis.

Method (Lead Author, Figure)	Catalyst/Key Conditions	Substrates/Partners	Maximum Yield	Stereoselectivity
Wang (Figure 18)	[Cp*RhCl_2_]_2_, Cu(OAc)_2_, TEMPO, HFIP, 80 °C, O_2_	*N*-Me-*N*-nitrosoanilines + diazo homophthalimides	up to 91%	-
Gu (Figure 19)	[Cp*RhCl_2_]_2_, MeCN, 80 °C	3-(2-isocyanoethyl) indoles + aryl azides	up to 87%	single diastereomer
Chen (Figure 20)	1-Rh_2_(OAc)_4_, DCE, 80 °C 2-AcOH, rt, N_2_	ester-tethered 1,2,3-triazoles	up to 87%	-
Tang (Figure 21)	Pd(OAc)_2_, dppb, Cs_2_CO_3_, mesitylene, 70–80 °C, Ar	3-(2-isocyanoethyl) indoles + aryl/vinyl halides	up to 98%	SPINOL-phosphoramidite ligand asymmetric variant 85% *ee*
Chen (Figure 22)	1-Pd(PPh_3_)_4_, THF, 80 °C, N_2_ 2-CPA, DCE, −10 °C	3-(2-isocyanoethyl) indoles + *α*-diazoesters	up to 71%	82:18–93:7 *er*
Lindman (Figure 23)	Pd(*^t^*Bu_3_P)_2_, Et_3_N, DMF, 80 °C	(+)-Vince-lactam-derived cyclopentenyl 2-bromo-*N*-methylanilines	up to 83%	>98% *dr* (*anti*)
Buttard (Figure 24)	Au(I): Au Cat 1, toluene, 70 °C Pd(0): Pd_2_(dba)_3_, L5, Cs_2_CO_3_, DCM, 60 °C, Ar	tryptamines	Au: up to 95% Pd: up to 87%	Pd: up to >20:1 *dr* and 77% *ee*
Xing (Figure 25)	1-TBAF, THF, rt 2-Cu(OAc)_2_, 4 Å MS, DMF, 160 °C	*N*-sulfonylaziridines	up to 85%	-
Xu (Figure 26)	Cu(MeCN)_4_PF_6_, toluene, 30 °C	indolyl ynamides	up to 97%	>25:1 *dr*
Huang (Figure 27)	Cu(MeCN)_4_BF_4_, toluene, 110 °C, N_2_	enaminothiones + diazoindolinimines	up to 84%	-
Zhu (Figure 28)	IPrAuPhCN·SbF_6_, THF, 100 °C, Ar	tryptamine enynamides	up to 87%	single diastereomer
Zhu (Figure 29)	exo-dig: Ph_3_PAuCl/AgNTf_2_, Hantzsch ester, DCM, rt endo-dig: Ph_3_PAuCl/AgNTf_2_, Hantzsch ester, DCE, 80 °C	*N*-propargyl indoles	exo-dig: up to 82% endo-dig: up to 75%	-
Zaman (Figure 30)	Au(PPh_3_)Cl, AgOTf, CHCl_3_, rt	4-bromo-1*H*-indole-3- carbaldehyde-derived Ugi propargylamides	up to 92%	-
Dong (Figure 31)	AgPF_6_, TfOH, toluene, 40 °C	indoles + enynones	up to 95%	>19:1 *dr*
Duan (Figure 32)	AgNO_3_, MeCN, rt, air	indolyl ynones + phosphine oxides	up to 99%	-
Liang (Figure 33)	AgOTf, NFSI + Hantzsch ester, toluene, rt	tryptamine-ynamides	up to 99%	-
Bag & Sawant (Figure 34)	AgOTf, DCM, rt	indole-tethered ynones + Nu	up to 98%	single diastereomer
Liang (Figure 35)	AgOTf, PPh_3_, toluene, rt	tryptamine-ynesulfonamides + carbamates Nu	up to 99%	-
Jiang (Figure 36)	Mn(acac)_3_, MeCN, 80 °C, Ar	tryptamine-derived isocyanides + arylboronic acids	up to 98%	>20:1 *dr*
Yuan (Figure 37)	Co(acac)_2_, MeCN, 40 °C, N_2_ or CoC_2_O_4_, H_2_O, MeCN, 40 °C, air	tryptamine-derived isocyanides + iodonium ylides	up to 96% hydration: up to 80%	hydration: up to >20:1 *dr*
Li (Figure 38)	Y(OTf)_3_, 4 Å MS, DCM, rt	3-(2-isocyanoethyl)indoles + diester aziridines	up to 92%	-
Lin (Figure 39)	BiCl_3_, 5 Å MS, DCE, 100 °C, N_2_	tryptamine-ynamides	up to 90%	single diastereomer
Yamaoka (Figure 40)	Zn(OTf)_2_, HFIP, dioxane/hexane, 50 °C	enamide-ynamides + trimethylsilyl cyanide (TMSCN)	up to 92%	up to 91:9 *dr*
Zhao (Figure 41)	In(OTf)_3_, 3 Å MS, DCM, rt	allenamides + methylenemalonate	up to 90%	>99:1 *Z/E*, >99:1 *dr*
Zheng (Figure 42)	*anti*: Mg(OTf)_2_, CHCl_3_, rt *syn*: Mg(OTf)_2_, CHCl_3_, rt, HOAc	azomethine imines + indolyl isocyanides	*anti*: up to 93% *syn*: up to 90%	*anti*: up to 8.4:1 *dr* *syn*: sole isomer

**Table 3:** Summary of metal-free, photocatalytic and organocatalytic methods for C3-spiroindoline synthesis.

Method (Lead Author, Figure)	Catalyst/Key Conditions	Substrates/Partners	Maximum Yield	Stereoselectivity
Liu (Figure 43)	C-N spiroindoline: *^t^*BuOCl, TMEDA, DCM, rt C-C spiroindoline: *^t^*BuOCl, TMEDA, AlCl_3_, DCM, rt	THβ-carbolines + anilines/indoles	C-N spiroindoline: up to 95% C-C spiroindoline: up to 94%	-
Qin (Figure 44)	Cs_2_CO_3_, MeCN, 25 °C	*p*-QMs + sulfonyl indoles	up to 98%	up to 19:1 *dr*
Yuan (Figure 45)	DABCO, EtOH, 40 °C, Ar	tryptamine-derived isocyanides + Togni II	up to 82%	-
Gharpure (Figure 46)	Cs_2_CO_3_, EtOH, rt	sulfone-tethered alkynyl carbamates	up to 80%	≥19:1 *dr*
Chen (Figure 47)	catalyst-free, CHCl_3_:THF (3:1), 80 °C, N_2_	3-(2-isocyanoethyl)indoles + sulfonyl triazoles	up to 91%	-
Zhao (Figure 48)	Selectfluor, MeCN:H_2_O (100:1), rt, Ar	pyrrole-2-carboxamides	up to 90%	-
Bag (Figure 49)	NIS, 1,2-DCE, rt	indole-tethered ynones + Nu	up to 94%	single diastereomer
Ueda (Figure 50)	maleic acid/thiourea, MeCN, rt	diazo-functionalized indoles	up to 94%	-
Zhang (Figure 51)	IBX, HFIP:H_2_O (3:1), 60 °C	tryptophan derivatives	up to 96%	up to 95:5 *dr*
Yasui (Figure 52)	triphosgene or thiophosgene, Et_3_N, dioxane, 105 °C	tryptamines	up to 89% thio: up to 81%	chiral amine gave 5:1 *dr*
Dai (Figure 53)	(2 + 1): K_2_CO_3_, MeCN, rt (1 + 1 + 1): Cs_2_CO_3_, BF_3_·OEt_2_, toluene, rt	(2 + 1): aza-dienes + sulfonium bromides or Corey-Chaykovsky reagent (1 + 1 + 1): aza-dienes + sulfonium bromides + aryl aldehydes	(2 + 1): up to 94% (1 + 1 + 1): up to 73%	(2 + 1): up to >20:1 *dr* (1 + 1 + 1): up to 7:1 *dr*
Wang (Figure 54)	K_3_PO_4_ or Cs_2_CO_3_, DCE, 50 °C	tryptamine-derived isocyanides + hydrazonyl chlorides	up to 79%	-
Wang (Figure 55)	catalyst-free, dioxane, 120 °C	tryptamine-derived isocyanides + quinone esters	up to 96%	-
Zhang (Figure 56)	Cs_2_CO_3_, THF, rt	alkenyl-iminoindolines + *α*-halohydroxamates	up to 99%	>19:1 *dr*
Lv (Figure 57)	3DPA2FBN, K_2_CO_3_, blue LED, DMF, rt	isocyanide-indoles + CF_3_Br	up to 88%	>18:1 *dr*
Moustakim (Figure 58)	UV-A flow, benzene, 30 °C	aryl-enamine	up to quantitative yield	-
Ranga Rao (Figure 59)	K_2_S_2_O_8_, blue LED, DCE, rt, N_2_	isocyanoethylindoles + *α*-oxocarboxylic acids	up to 82%	single diastereomer
Yang (Figure 60)	PC-I, HFIP, blue LED, Ar	amide/pyridine indoles	up to 87%	>20:1 *dr*
Wang (Figure 61)	L-proline, EtOH, 60 °C	indazolamine + isatin + 1,3-dione	up to 93%	-
Pan (Figure 62)	SPINOL-CPA, DCM, 3 Å MS, 0 °C	indolyl dihydropyridines	up to 99%	>50:1 *dr*, 92:8 *er*
Zhang (Figure 63)	BINOL-IDPA, CHCl_3_, 20 °C	indolylmethanols + styrylnaphthols	up to 92%	99% *ee*, >19:1 *dr*

**Table 4:** Catalytic versatility of 3-(2-isocyanoethyl)indole and related tryptamine-derived isocyanide platforms.

Catalyst Class	Coupling Partner	Product Framework	Maximum Yield	Stereoselectivity Outcome	Figure Number
Pd(OAc)_2_, dppb	aryl/vinyl halides	2′-aryl/vinyl spiroindolines	98%	up to 85% *ee*	Figure 21
Pd(PPh_3_)_4_, CPA	*α*-diazo esters	pentacyclic spiroindolines	71%	82:18–93:7 *er*	Figure 22
[Cp*RhCl_2_]_2_	aromatic azides	polycyclic spiroindolines with pentasubstituted guanidine	87%	single diastereomer	Figure 19
Y(OTf)_3_ (Lewis acid)	2,2′-diester aziridines	polycyclic spiroindolines with tetrahydro-*β*-carboline core	92%	-	Figure 38
Co(acac)_2_/CoC_2_O_4_	iodonium ylides (± H_2_O)	spiroindolines or hydroxy-spiroindolines (*syn*)	96%	>20:1 *dr*; switchable by *N*-substituent	Figure 37
Mn(acac)_3_	arylboronic acids	spiroindolenines reducible to *cis*-spiroindolines	98%	>20:1 *dr* after reduction	Figure 36
Mg(OTf)_2_	*N*,*N’*-cyclic azomethine imines	[6,5,5,6,5] pentacyclic spiroindolines	93%	diastereodivergent (*anti* or *syn*) via HOAc	Figure 42
Catalyst-free	sulfonyl triazoles	*α*-carboline-containing [6,5,5,6] tetracyclic spiroindolines	91%	-	Figure 47
Catalyst-free	quinone esters	chromenoindoles → polycyclic spiroindolines (after NaBH_3_CN)	96%	-	Figure 55
DBU (base)	Ugi-derived adducts (post-Ugi)	benzo-fused spiroindolines	99%	-	Figure 15
Visible-light (3DPA2FBN)	CF_3_Br (radical)	trifluoromethylated 3-spiroindolines (spiro[indole-3,3-quinoline]/spiro[indole-3,3-pyrrole])	88%	up to 18:1 *dr*	Figure 57
Visible-light (K_2_S_2_O_8_)	*α*-oxocarboxylic acids	*N*-formyl-containing [6,5,5,5] tetracycles	82%	single diastereomer	Figure 59
DABCO (base) + Togni II	CF_3_ radical (from Togni II)	C2-substituted: CF_3_-spiroindolines	82%	-	Figure 45
K_3_PO_4_ or Cs_2_CO_3_	hydrazonyl chlorides (nitrile imines, in situ)	[6,5,5,6] tetra- or [6,5,5,6,5] pentacyclic bispiroindoline	79%	-	Figure 54

## Data Availability

No new data were created in this study.

## Supplementary Materials

The following supporting information can be downloaded at https://www.mdpi.com/article/10.3390/molecules31142518/s1.
