# Supplementary material for: Recent Advances in the Synthesis of Spiroindolines: Catalytic Strategies, Stereoselectivity, and Synthetic Utility (2020–2025)
Source: Molecules. 2026 Jul 19;31(14):2518. doi: 10.3390/molecules31142518 (PMC13415220; doi:10.3390/molecules31142518)
Supplement: Supplementary file 1 [file molecules-31-02518-s001.zip › molecules-4362576-supplementary.pdf]

Supplementary Information

Recent Advances in the Synthesis of Spiroindolines: Catalytic Strategies, Stereoselectivity, and Synthetic Utility (2020–2025)

AUTHOR NAMES

Parthiena M. Keddis[a], *Ahmed Mamdouh Antar*[a], *Trevina M. Keddis*[a], *Youssef Aboushady*[a], *Ashraf H. Abadi*[a], *Grigoris Zoidis*[b], *Matthias Engel*[c]*, *Mohammad Abdel-Halim*[a]*, *Mennatallah Abdallah*[a]

AUTHOR ADDRESS

[a]Department of Pharmaceutical Chemistry, Faculty of Pharmacy and Biotechnology, German University in Cairo, Cairo 11835, Egypt

[b]Division of Pharmaceutical Chemistry, Department of Pharmacy, School of Health Sciences, National and Kapodistrian University of Athens, Panepistimiopolis-Zografou, 15771 Athens, Greece

[c]Pharmaceutical and Medicinal Chemistry, Saarland University, Campus C2.3, D-66123 Saarbrücken, Germany

*Corresponding author: Matthias Engel (ma.engel@mx.uni-saarland.de) and Mohammad Abdel-Halim (mohammad.abdel-halim@guc.edu.eg)

Table of Contents

Table S1. Optimization for Yang et al. - Rh(III) [3+2] spiroannulation (Fig. 3). 4

Table S2. Optimization for Li et al. - Rh(III) dearomative spirocyclization with alkynes (Fig. 4). 4

Table S3. Optimization for Rupa & Anbarasan - $Rh_2(OAc)_4$ [4+1] annulation (Fig. 5)................................ 4

Table S4. Optimization for Yu et al. - Ru(II) condition-controlled [4+1] (Fig. 6). 4

Table S5. Optimization for Zhang et al. - Pd vinylogous arylation/aza-Michael (Fig. 7). 4

Table S6. Optimization for Gao & Jiao - Pd asymmetric dearomatization (Fig. 8)...................................... 4

Table S7. Optimization for Chang et al. - Pd/Et_3B benzylation-semipinacol (Fig. 9). 5

Table S8. Optimization for Lei et al. - Cu aerobic oxidative cascade (Fig. 10).. 5

Table S9. Optimization for Panda & Ghorai - chiral phosphoric acid (Heyns) (Fig. 11). 5

Table S10. Optimization for Xie et al. - oxyallyl cation semipinacol (Fig. 12).. 5

Table S11. Optimization for Sah et al. - organocatalytic aza-Michael (Fig. 13). 5

Table S12. Optimization for Carceller-Ferrer et al. - squaramide formal [4+1] (Fig. 14). 6

Table S13. Optimization for Zhao et al. - DBU post-Ugi/Conia-ene (Fig. 15). 6

Table S14. Optimization for Dong et al. - catalyst-free *gem*-difluorination (Fig. 16). 6

Table S15. Optimization for Wang et al. - Rh(III) C(sp^2)-H/C(sp^3)-H [4+1] (Fig. 18). 6

Table S16. Optimization for Gu et al. - Rh cascade [4+2] (Fig. 19)... 6

Table S17. Optimization for Chen et al. - Rh 1,2-acyloxy/aza-[4+2] (Fig. 20).. 7

Table S18. Optimization for Tang et al. - Pd imidoylative spirocyclization (Fig. 21). 7

Table S19. Optimization for Chen et al. - Pd then CPA Mannich (Fig. 22). 7

Table S20. Optimization for Lindman et al. - intramolecular Mizoroki-Heck (Fig. 23). 7

Table S21. Optimization for Buttard et al. - Au(I) cycloisomerization and asymmetric Pd(0) (Fig. 24). 8

Table S22. Optimization for Xing et al. - Cu C-H activation/cyclization (Fig. 25)....................................... 8

Table S23. Optimization for Xu et al. - Cu dearomative *β*-additon (Fig. 26). 8

Table S24. Optimization for Huang et al. - Cu(I) [4+1] annulation (Fig. 27). 8

Table S25. Optimization for Zhu et al. - Au(I) *β*-cycloisomerization (Fig. 28).. 8

Table S26. Optimization for Zhu et al. - Au(I) substitution-controlled cyclization (Fig. 29)......................... 8

Table S27. Optimization for Dong et al. - Bronsted acid/Ag cooperative (T-divergent) (Fig. 31)................ 8

Table S28. Optimization for Duan et al. - Ag dearomative phosphonylation (Fig. 32)................................. 9

Table S29. Optimization for Liang et al. - AgOTf/NFSI (Hantzsch ester) (Fig. 33). 9

Table S30. Optimization for Bag & Sawant - Ag(I) spirocyclization/trapping (Fig. 34). 9

Table S31. Optimization for Liang et al. - AgOTf/PPh_3 carbamate trapping (Fig. 35). 9

Table S32. Optimization for Jiang et al. - Mn(III) radical addition/spirocyclization (Fig. 36). 9

Table S33. Optimization for Yuan et al. - Co(II) coupling-spirocyclization (Fig. 37). 10

Table S34. Optimization for Li et al. - Y(III) cascade with aziridines (Fig. 38). 10
Table S35. Optimization for Lin et al. - $BiCl_3$ tandem cyclization (Fig. 39). 10
Table S36. Optimization for Yamaoka et al. - Lewis acid domino with TMSCN (Fig. 40). 10
Table S37. Optimization for Zhao et al. - In(III) [2+2+2] allenamide cascade (Fig. 41). 11
Table S38. Optimization for Zheng et al. - Mg(II) diastereodivergent cascade (Fig. 42). 11
Table S39. Optimization for Liu et al. - rearrangement coupling of tetrahydro-β-carbolines (Fig. 43). 11
Table S40. Optimization for Qin et al. - diastereoselective chroman/spiroindolenine (Fig. 44). 11
Table S41. Optimization for Yuan et al. - metal-free radical CF_3 annulation (Fig. 45). 11
Table S42. Optimization for Chen et al. - catalyst-free with sulfonyl triazoles (Fig. 47). 11
Table S43. Optimization for Zhao et al. - Selectfluor oxidative spirocyclization (Fig. 48). 12
Table S44. Optimization for Bag et al. - NIS ipso-iodocyclization/addition (Fig. 49). 12
Table S45. Optimization for Ueda et al. - cooperative acid/thiourea ipso-FC (Fig. 50). 12
Table S46. Optimization for Zhang et al. - IBX oxidative C2/C3 cyclization (Fig. 51). 12
Table S47. Optimization for Yasui et al. - (thio)chloroformylation/spirocyclization (Fig. 52). 12
Table S48. Optimization for Dai et al. - sulfur ylide (2+1)/(1+1+1) (Fig. 53). 13
Table S49. Optimization for Wang et al. - base-controlled hydrazonyl annulation (Fig. 54). 13
Table S50. Optimization for Wang et al. - catalyst-free with quinone esters (Fig. 55). 13
Table S51. Optimization for Zhang et al. - base-promoted formal [3+2] (Fig. 56). 13
Table S52. Optimization for Lv et al. - visible-light radical CF_3/cyclization (Fig. 57). 14
Table S53. Optimization for Moustakim et al. - UV-A continuous-flow photocyclization (Fig. 58). 14
Table S54. Optimization for Ranga Rao et al. - visible-light/$K_2S_2O_8$ cascade (Fig. 59). 14
Table S55. Optimization for Yang et al. - visible-light energy-transfer (T-ESPT) (Fig. 60). 14
Table S56. Optimization for Wang et al. - L-proline three-component condensation (Fig. 61). 14
Table S57. Optimization for Pan et al. - SPINOL-CPA asymmetric cascade (Fig. 62). 14
Table S58. Optimization for Zhang et al. - BINOL-IDPA [3+2]/spirocyclization (Fig. 63). 15

Each table lists the optimal (standard) conditions and the principal deviations screened, transcribed from the corresponding primary report.

Table S1. Optimization for Yang et al. - Rh(III) [3+2] spiroannulation (Fig. 3).

Entry	Deviation from standard conditions	Yield / outcome
**1**	none (standard conditions)	73%
**2**	lower catalyst loading	lower yield
**3**	$[Cp^*RhCl_2]_2$	lower yield
**4**	solvents other than toluene	lower yield
**5**	K_2CO_3 added (base)	reaction suppressed
**6**	decreased or increased TMB	lower yield
**7**	other acids than TMB	lower yield
**8**	no Rh catalyst	no product
**9**	no air	no product

Table S2. Optimization for Li et al. - Rh(III) dearomative spirocyclization with alkynes (Fig. 4).

Entry	Deviation from standard conditions	Yield / outcome
**1**	none (standard conditions)	85%
**2**	Ru- or Co-based catalyst	ineffective
**3**	Ir-based catalyst	significantly lower yield

Table S3. Optimization for Rupa & Anbarasan - $Rh_2(OAc)_4$ [4+1] annulation (Fig. 5).

Entry	Deviation from standard conditions	Yield / outcome
**1**	none (standard conditions)	90%
**2**	$Rh_2(Oct)_4$, $Rh_2(Piv)_4$ or $Rh_2(esp)_4$	59–67%
**3**	1,2-DCE, PhCl or DCM	47–75%
**4**	lower temperature	decreased yield
**5**	fewer equiv of diazo imines	decreased yield
**6**	more equiv of aminoacetophenone	no improvement

Table S4. Optimization for Yu et al. - Ru(II) condition-controlled [4+1] (Fig. 6).

Entry	Deviation from standard conditions	Yield / outcome
**1**	none (standard conditions)	80%
**2**	aerobic (O_2) conditions	diverts to spirodihydroquinoline ([5+1])
**3**	alternative solvents	diminished yield
**4**	alternative catalysts	diminished yield

Table S5. Optimization for Zhang et al. - Pd vinylogous arylation/aza-Michael (Fig. 7).

Entry	Deviation from standard conditions	Yield / outcome
**1**	none (standard conditions)	81%
**2**	DMF, NMP or DMSO	moderate yield
**3**	THF, toluene, DME or 1,4-dioxane	no product
**4**	no Pd catalyst	no reaction
**5**	no base	no reaction

Table S6. Optimization for Gao & Jiao - Pd asymmetric dearomatization (Fig. 8).

Entry	Deviation from standard conditions	Yield / outcome
**1**	none (standard conditions)	88%, 95:5 *er*
**2**	fluoro-substituted ligand (L8)	89%, 92:8 *er*
**3**	NHC, BINAP, spiro-phosphoramidite, Feringa, sterically hindered or phosphoramidite ligands	significantly reduced yield
**4**	higher temperature	reduced conversion

**5**	^{t}BuONa, Cs_2CO_3, ^{t}BuOLi or ^{i}PrOLi	reduced/no conversion
**6**	Ts, Ac or Me protecting group	reduced/no conversion
**7**	$Pd(OAc)_2$ or $[Pd(C_3H_5)Cl]_2$	reduced/no conversion

Table S7. Optimization for Chang et al. - Pd/Et_3B benzylation-semipinacol (Fig. 9).

Entry	Deviation from standard conditions	Yield / outcome
**1**	none (standard conditions)	~88%, >20:1 *dr*
**2**	no Et_3B or $Ti(OEt)_4$ / $Fe(OTf)_2$ instead	no product
**3**	leaving groups OH, OAc, OBoc, OCO_2Me	poor/no conversion
**4**	ligands BINAP, DPPF, DPPB, DPPP, XPhos	poor/no conversion
**5**	DPEphos ligand	41%, >20:1 *dr*
**6**	toluene or ^{i}PrOH	lower yield
**7**	MeCN	traces
**8**	$Pd(OAc)_2$, $(ClPdC_3H_5)_2$, $PdCl_2$, $Ni(COD)_2$	traces
**9**	$Pd(dba)_2$	slightly less/comparable to optimum

Table S8. Optimization for Lei et al. - Cu aerobic oxidative cascade (Fig. 10).

Entry	Deviation from standard conditions	Yield / outcome
**1**	none (standard conditions)	86%
**2**	alternative Cu sources	diminished yield
**3**	toluene	48%
**4**	DMF, NMP, dioxane, THF	<10%
**5**	higher temperature	deteriorated yield
**6**	N_2 instead of air	deteriorated yield

Table S9. Optimization for Panda & Ghorai - chiral phosphoric acid (Heyns) (Fig. 11).

Entry	Deviation from standard conditions	Yield / outcome
**1**	none (standard conditions)	75%, >20:1 *dr*, 96% *ee*
**2**	Bronsted base, or amino-thiourea/squaramide catalysts	Unsatisfactory yields
**3**	polar solvents (THF, MeCN)	poor conversion and *ee*
**4**	non-polar ethereal solvents	65–89% *ee*
**5**	20 mol% catalyst	no further improvement
**6**	desiccant other than 4 Å MS	inferior

Table S10. Optimization for Xie et al. - oxyallyl cation semipinacol (Fig. 12).

Entry	Deviation from standard conditions	Yield / outcome
**1**	none (standard conditions)	89%, 10.8:1 *dr*
**2**	DEA	67%, 5.5:1 *dr*
**3**	DMAP	49%, 3.5:1 *dr*
**4**	Na_2CO_3	52%, 5.2:1 *dr*
**5**	K_2CO_3	58%, 5.8:1 *dr*
**6**	DCM / toluene	73–78%, lower *dr*
**7**	THF / MeCN / TFE	36–55%, lower *dr*

Table S11. Optimization for Sah et al. - organocatalytic aza-Michael (Fig. 13).

Entry	Deviation from standard conditions	Yield / outcome
**1**	none (standard conditions)	75%, 94:6 *er*
**2**	TFA additive	lower *er*
**3**	*N*-Boc-phenylglycine	moderate yield, good *er*
**4**	*N*-Boc-L-proline	*er* abolished
**5**	*N*-Boc-L-phenylalanine	yield dropped to 10%

**6**	benzoic acid derivatives with other organocatalysts	Failed yield and *er*
**7**	solvents other than DCM	ineffective
**8**	alternative protecting groups	no improvement

Table S12. Optimization for Carceller-Ferrer et al. - squaramide formal [4+1] (Fig. 14).

Entry	Deviation from standard conditions	Yield / outcome
**1**	none (standard conditions)	62%, 93% *ee*
**2**	quinine-derived thiourea	39%, 0% *ee*
**3**	quinine-derived squaramide	35%, 30% *ee*
**4**	Rawal's squaramide	40% *ee*
**5**	diaminocyclohexane squaramides	slightly higher yield, lower *ee*
**6**	1.5 equiv aza-*o*-QM and base	59%, 81% *ee*
**7**	other solvents than $CHCl_3$	inferior
**8**	other bases than Na_2CO_3	inferior
**9**	higher reaction concentration	91% *ee*
**10**	10 mol% catalyst	no additional benefit
**11**	reaction under N_2	no significant change
**12**	40 °C or -20 °C	reduced yield and *ee*

Table S13. Optimization for Zhao et al. - DBU post-Ugi/Conia-ene (Fig. 15).

Entry	Deviation from standard conditions	Yield / outcome
**1**	none (standard conditions)	98%
**2**	DMAP, DABCO, PPh_3, DPPE, DPPB, DIPEA, TEA	completely ineffective
**3**	NaOEt / K_2CO_3 / NaOH	82% / 87% / 39%
**4**	MeCN instead of MeOH	88%
**5**	10% baking-soda water	52%
**6**	refluxing water	53% (84% with microwave)

Table S14. Optimization for Dong et al. - catalyst-free *gem*-difluorination (Fig. 16).

Entry	Deviation from standard conditions	Yield / outcome
**1**	none (standard conditions)	79%
**2**	air instead of Ar	73%
**3**	DMF / DMA	53% / 66% (24 h)
**4**	toluene or DCM	no reaction after 24 h, poor solubility
**5**	NFSI as F source	21%
**6**	CsF or TBAF	entirely ineffective
**7**	50 °C	56%
**8**	2.5 equiv Selectfluor	49%
**9**	added water	69%
**10**	acidic conditions	decreased efficiency/reaction failure

Table S15. Optimization for Wang et al. - Rh(III) C(sp^2)-H/C(sp^3)-H [4+1] (Fig. 18).

Entry	Deviation from standard conditions	Yield / outcome
**1**	none (standard conditions)	82%
**2**	AgOAc in HFIP under air	40%
**3**	TFE, MeOH, toluene, 1,4-dioxane	ineffective
**4**	CsOAc, NaOAc, $Zn(OAc)_2$, $AgSbF_6$	inferior
**5**	PivOH, HOAc, TBHP	inferior
**6**	$CoCp(CO)I_2$, $[IrCpCl_2]_2$, [Ru(*p*-cymene)$Cl_2]_2$	product suppressed

Table S16. Optimization for Gu et al. - Rh cascade [4+2] (Fig. 19).

Entry	Deviation from standard conditions	Yield / outcome
**1**	none (standard conditions)	61%, single diastereomer

**2**	various Pd and Rh salts	32–52%
**3**	THF, toluene, DCE	decreased yield
**4**	1,4-dioxane	reaction suppressed completely
**5**	higher or lower temperature	reduced yield

Table S17. Optimization for Chen et al. - Rh 1,2-acyloxy/aza-[4+2] (Fig. 20).

Entry	Deviation from standard conditions	Yield / outcome
**1**	none (standard conditions)	85%
**2**	non-acidic conditions	ineffective
**3**	alternative catalyst loading / temperature	no improvement

Table S18. Optimization for Tang et al. - Pd imidoylative spirocyclization (Fig. 21).

Entry	Deviation from standard conditions	Yield / outcome
**1**	none (standard conditions)	98%
**2**	toluene, THF, MeCN	25–78%
**3**	$Pd(TFA)_2$ / $PdCl_2$	49% / 78%
**4**	PPh_3, PCy_3, BINAP	25–81%
**5**	Na_2CO_3, K_2CO_3, DBU, Et_3N	lower yield
**6**	lower temperature or 5 mol% catalyst	lower yield

Table S19. Optimization for Chen et al. - Pd then CPA Mannich (Fig. 22).

Entry	Deviation from standard conditions	Yield / outcome
**1**	none (standard conditions)	Pd: 84%; CPA: 65%, 87:13 *er*
**2**	$PdCl_2$, $Pd(PPh_3)_2Cl_2$, $Pd(OAc)_2$, $Pd(OCOCF_3)_2$	Pd: ≤13%
**3**	CH_3CN	Pd: 56–59%; CPA: 56%, 52:48 *er*
**4**	DCM	Pd: 19%; CPA: 67%, 72:28 *er*
**5**	DCE	Pd: 40%
**6**	toluene	Pd: 41%; CPA: 61%, 77:23 *er*
**7**	EtOAc	Pd: 70%; CPA: 20%, 69:31 *er*
**8**	1,4-dioxane	Pd: 69%
**9**	THF	CPA: 23%, 54:46 *er*
**10**	5% or 20% catalyst loading	Pd: lower yield or no improvement
**11**	lower or higher temperature	Pd: slightly lower yield; CPA: lower yield or lower *er*
**12**	longer reaction time	Pd: lower yield or no improvement; CPA: slightly lower yield
**13**	other CPA catalysts	CPA: 10–71% yield but lower *er*

Table S20. Optimization for Lindman et al. - intramolecular Mizoroki-Heck (Fig. 23).

Entry	Deviation from standard conditions	Yield / outcome
**1**	none (standard conditions)	83%, >98% diastereoselectivity to *anti*
**2**	100 or 120 °C	no improvement in conversion
**3**	5 mol% dppf or $Pd(dppf)Cl_2$	significantly lower conversion
**4**	PPh_3 ligand	less effective (electron-rich aryl bromide; sluggish oxidative addition)
**5**	no Pd	<1% conversion
**6**	no base	3% conversion
**7**	secondary *N*-allylaniline (vs *N*-Me)	unstable, byproducts and low yield

Table S21. Optimization for Buttard et al. - Au(I) cycloisomerization and asymmetric Pd(0) (Fig. 24).

Entry	Deviation from standard conditions	Yield / outcome
**1**	none (standard conditions)	Au 95%; Pd 47%, >20:1 *dr*, 76/64 *ee*
**2**	other Au catalysts or 5 mol% loading	incomplete conversion
**3**	DCE or 50 °C (Au step)	favors spiroindolinone byproduct
**4**	DCM at 0.05 M (Pd step)	61%, 19:1 *dr*, 68/70 *ee*
**5**	other ligands/loadings (Pd step)	lower yield and stereoselectivity

Table S22. Optimization for Xing et al. - Cu C-H activation/cyclization (Fig. 25).

Entry	Deviation from standard conditions	Yield / outcome
**1**	none (standard conditions)	72%
**2**	5 mol% $Cu(OAc)_2$	lower yield
**3**	120 °C	lower yield
**4**	added K_2CO_3 or $Pd(OAc)_2$	lower yield
**5**	DMA instead of DMF	lower yield

Table S23. Optimization for Xu et al. - Cu dearomative *β*-additon (Fig. 26).

Entry	Deviation from standard conditions	Yield / outcome
**1**	none (standard conditions)	95%, >25:1 *dr*
**2**	$Cu(OTf)_2$	low yield
**3**	Au or Ag catalysts	low yield
**4**	Bronsted acid catalysts	substrate decomposition, no product

Table S24. Optimization for Huang et al. - Cu(I) [4+1] annulation (Fig. 27).

Entry	Deviation from standard conditions	Yield / outcome
**1**	none (standard conditions)	84% (imines)
**2**	other Cu(I) catalysts	lower yield
**3**	Rh, Pd, Ag, Ir catalysts	ineffective
**4**	solvents/temperatures other than toluene/110 °C	inferior
**5**	3-diazooxindoles (vs imines)	use CuCN, 5 h

Table S25. Optimization for Zhu et al. - Au(I) *β*-cycloisomerization (Fig. 28).

Entry	Deviation from standard conditions	Yield / outcome
**1**	none (standard conditions)	82%, single diastereomer
**2**	Ag salts or Bronsted acid catalysts	ineffective / much less efficient
**3**	anhydrous DCE	47%

Table S26. Optimization for Zhu et al. - Au(I) substitution-controlled cyclization (Fig. 29).

Entry	Deviation from standard conditions	Yield / outcome
**1**	none (standard conditions)	5-exo 81%; 6-endo 71%
**2**	2 equiv Hantzsch ester	5-exo: 31% (+47% Wagner-Meerwein byproduct)
**3**	4 equiv Hantzsch ester	5-exo: 66% (+24% byproduct)
**4**	5 mol% catalyst	5-exo: 43%
**5**	$AgSbF_6$ / $AgBF_4$	5-exo: 60% / 54%
**6**	room temperature	6-endo: 31%
**7**	toluene at 80 °C	6-endo: 62%

Table S27. Optimization for Dong et al. - Bronsted acid/Ag cooperative (T-divergent) (Fig. 31).

Entry	Deviation from standard conditions	Yield / outcome
**1**	none (standard conditions)	92% (spiroindolenine, >19:1 *dr*)
**2**	$AgBF_4$, AgOTf, $AgNO_3$	significantly reduced yield
**3**	salicylic acid, TFA, TsOH	decreased yield

**4**	non-anhydrous solvents	lower yield
**5**	25 °C	slightly lower yield
**6**	60 °C	[1,2]-migration -> dihydrocyclohepta[*b*]indolone
**7**	80 °C	higher yield of indole-fused product

Table S28. Optimization for Duan et al. - Ag dearomative phosphonylation (Fig. 32).

Entry	Deviation from standard conditions	Yield / outcome
**1**	none (standard conditions)	99%
**2**	AgOTf in DCM	99%
**3**	Cu(OTf)$_2$ or TFA	92–94%
**4**	DMF, dioxane, toluene	87–91%
**5**	5 mol% catalyst	92%
**6**	no catalyst	no reaction

Table S29. Optimization for Liang et al. - AgOTf/NFSI (Hantzsch ester) (Fig. 33).

Entry	Deviation from standard conditions	Yield / outcome
**1**	none (standard conditions)	99%
**2**	without NFSI	8 h to reach 90%
**3**	$NaBH_4$, $NaBH_3CN$, NaBH(OAc)$_3$, $LiBHEt_3$, DIBAL-H	0%
**4**	Et_3SiH	7% (+22% rearranged)
**5**	DCM / THF / DCE	73% / 75% / 45%
**6**	MeCN	no reaction
**7**	0 °C	6%
**8**	3 mol% catalyst	5%
**9**	10 mol% NFSI	15% (catalyst over-coordination)

Table S30. Optimization for Bag & Sawant - Ag(I) spirocyclization/trapping (Fig. 34).

Entry	Deviation from standard conditions	Yield / outcome
**1**	none (standard conditions)	98% (single diastereomer)
**2**	PPh_3AuCl or HAuCl4.3H_2O	ineffective
**3**	$AgNO_3$ / $AgBF_4$ / $AgSbF_6$ / CF_3COOAg / Ag(II) picolinate	42 / 61 / 58 / 68 / 40%
**4**	Cu(OTf)$_2$ / Cu(MeCN)$_4BF_4$	54% / 57%
**5**	THF, 1,4-dioxane, DMF, toluene	decreased yield
**6**	1 mol% or 5 mol% AgOTf	94–97%

Table S31. Optimization for Liang et al. - AgOTf/PPh_3 carbamate trapping (Fig. 35).

Entry	Deviation from standard conditions	Yield / outcome
**1**	none (standard conditions)	98%
**2**	water, MeOH, H_2NAc, H_2NSO_2Ph, HN(Boc)$_2$, H_2NPh	fail; rearranged product major
**3**	H_2NPh	fail
**4**	H_2NCO_2Me	43% spiro + 22% rearranged
**5**	DCM / THF	inferior selectivity
**6**	MeCN	no product (catalyst deactivation)
**7**	0 °C	50% spiro + 21% rearranged (48 h)
**8**	50 °C	only rearranged product (51%)
**9**	added NFSI	detrimental (only rearranged)
**10**	*N*- or thiourea-based ligands	0–42%
**11**	phenyl-phosphine ligands	35–90%

Table S32. Optimization for Jiang et al. - Mn(III) radical addition/spirocyclization (Fig. 36).

Entry	Deviation from standard conditions	Yield / outcome
**1**	none (standard conditions)	95%

**2**	$Mn(OAc)_3.2H_2O$	significantly less effective
**3**	toluene, DMF, EtOH, 1,4-dioxane	lower yield
**4**	25-60 °C	reduced yield
**5**	100 °C	no improvement
**6**	1:1 isocyanide:boronic acid	diminished efficiency
**7**	added TEMPO (2 equiv)	product almost fully suppressed (radical probe)

Table S33. Optimization for Yuan et al. - Co(II) coupling-spirocyclization (Fig. 37).

Entry	Deviation from standard conditions	Yield / outcome
**1**	none (standard conditions)	85%
**2**	CoC_2O_4, $[Rh(cod)Cl]_2$	57–71%
**3**	$Pd(OAc)_2$, CuI, Co(TPP)	0–19%
**4**	DMF, DCM, DCE, toluene, CH_3OH	39–81%
**5**	25 °C / 60 °C / 80 °C	85% / 56% / 82%
**6**	$PhNH_2$, n-$BuNH_2$, CH_3OH	Hydration: 0%
**7**	$Co(acac)_2$	Hydration: 0%
**8**	$CoCl_2$ / $Co(acac)_3$	Hydration: 30% / 44%
**9**	DCM, DCE, toluene, dioxane, water	Hydration: 0–45%

Table S34. Optimization for Li et al. - Y(III) cascade with aziridines (Fig. 38).

Entry	Deviation from standard conditions	Yield / outcome
**1**	none (standard conditions)	78%
**2**	no Lewis acid	no reaction
**3**	$Ni(ClO_4)_2.6H_2O$, $Al(OTf)_3$	no reaction
**4**	$Sc(OTf)_3$, $Zn(OTf)_2$	15% / 28%
**5**	$Yb(OTf)_3$, $Mg(OTf)_2$	75% / 66%
**6**	4 Å MS removed	59%
**7**	3 A / 5 Å MS	65% / 47%
**8**	solvent, temperature, concentration changes	no improvement

Table S35. Optimization for Lin et al. - $BiCl_3$ tandem cyclization (Fig. 39).

Entry	Deviation from standard conditions	Yield / outcome
**1**	none (standard conditions)	85% (single diastereomer)
**2**	$Zn(OTf)_2$, $Fe(OTf)_3$, $Bi(OTf)_3$, BiI_3, $BiBr_3$ (vs $BiCl_3$)	$BiCl_3$ best (65%)
**3**	4 A / 5 Å MS	68 / 70%
**4**	3 Å MS, Na_2SO_4, $MgSO_4$	lower yield
**5**	DCM, toluene, EtOAc, THF, MeCN, dioxane	inferior to DCE
**6**	80 °C	0–70%
**7**	50 °C	no reaction
**8**	30-40 mol% $BiCl_3$	no improvement

Table S36. Optimization for Yamaoka et al. - Lewis acid domino with TMSCN (Fig. 40).

Entry	Deviation from standard conditions	Yield / outcome
**1**	none (standard conditions)	85%, 90:10 *dr*
**2**	TfOH	91%, 60:40 *dr*
**3**	$Cu(OTf)_2$ / $Sc(OTf)_3$	lower yield
**4**	added HFIP	79%, 60:40 *dr*
**5**	added *t*BuOH	75%, 60:40 *dr*
**5**	temperature variation	little effect
**6**	hexane	9%, 90:10 *dr*
**7**	DCE	85%, 65:35 *dr*
**8**	toluene	47%, 80:20 *dr*
**9**	dioxane	77%, 85:15 *dr*

Table S37. Optimization for Zhao et al. - In(III) [2+2+2] allenamide cascade (Fig. 41).

Entry	Deviation from standard conditions	Yield / outcome
**1**	none (standard conditions)	87% (*dr* & *Z/E* >99:1)
**2**	$Cu(OTf)_2$ in THF	low yield 38%
**3**	$Cu(OTf)_2$ in DCM	61%
**4**	$CuBr_2$ or $NiCl_2$	no product
**5**	$FeCl_3$	unsatisfactory yield 41%
**6**	In(III) salts	78–84%, (*dr* 83:17–99:1)

Table S38. Optimization for Zheng et al. - Mg(II) diastereodivergent cascade (Fig. 42).

Entry	Deviation from standard conditions	Yield / outcome
**1**	none (standard conditions)	75%, 8:1:1 *dr*
**2**	no catalyst, or CuI / Ag_2CO_3	0–22%
**3**	$Mg(ClO_4)_2$	65%, 4:7:1 *dr*
**4**	TfOH, TsOH, MsOH, H_3PO_4, $Ni(ClO4)_2{\cdot}6H_2O$, $Sc(OTf)_3$, $In(OTf)_3$, $Cu(OTf)_2$, $Zn(OTf)_2$	not better than $Mg(OTf)_2$
**5**	5 mol% $Mg(OTf)_2$	69%, 5:1:1 *dr*
**6**	added HOAc	epimerizes to syn isomer (84%), <1:19 *dr*

Table S39. Optimization for Liu et al. - rearrangement coupling of tetrahydro-*β*-carbolines (Fig. 43).

Entry	Deviation from standard conditions	Yield / outcome
**1**	none (standard conditions)	C-N: 92%; C-C: 73%
**2**	$CHCl_3$, CCl_4, THF, CH_3CN, DCE, DMF, DMSO, acetone, DMA, dioxane, 2-Me-THF, MeOH, ethyl acetate	C-N: lower yield
**3**	$AlCl_3$	C-N: 88%
**4**	no Lewis acid	C-C: no reaction
**5**	FePc, $ZnCl_2$, CuI, $Cu(acac)_2$, $CuBr_2$, AcOH, 1N HCl, 1N H_2SO_4	C-C: lower yield

Table S40. Optimization for Qin et al. - diastereoselective chroman/spiroindolenine (Fig. 44).

Entry	Deviation from standard conditions	Yield / outcome
**1**	none (standard conditions)	94%, 19:1 *dr*
**2**	K_2CO_3, DBU, NaOH, KOH, KO^{t}Bu	lower yields
**3**	$CHCl_3$, CH_2Cl_2, DCE, dioxane, Et_2O, THF, toluene, xylene	lower yields
**5**	lower temperature	slightly reduced *dr* (decreased to 10:1)
**6**	higher temperature	no yield improvement

Table S41. Optimization for Yuan et al. - metal-free radical CF_3 annulation (Fig. 45).

Entry	Deviation from standard conditions	Yield / outcome
**1**	none (standard conditions)	80%
**2**	K_2CO_3, tBuOK, Na_2HPO_4	39–67%
**3**	KOH	trace
**4**	MeOH, MeCN, THF, DMSO	39-67%
**5**	1,4-dioxane	trace
**6**	substrate ratio 1:1.2 / 1:1.5	56% / 58%
**7**	20 °C / 60 °C	60% / 66%
**8**	air	55%
**9**	12 h	same as 6 h

Table S42. Optimization for Chen et al. - catalyst-free with sulfonyl triazoles (Fig. 47).

Entry	Deviation from standard conditions	Yield / outcome

**1**	none (standard conditions)	78%
**2**	$CHCl_3$/THF 1:1 or 5:1	lower yield
**3**	70 / 90 °C	lower yield 43 / 39%
**4**	triazole 1.5 equiv	lower yield 64%
**5**	indole 2.5 equiv	lower yield 72–78%
**6**	Rh catalyst	failed (isonitrile deactivation)
**7**	$CHCl_3$, DCM, CCl_4, DCE, TCE, toluene, PhCl, THF, dioxane, DME, MTBE, CH_3CN, $CHCl_3$/dioxane	lower yield

Table S43. Optimization for Zhao et al. - Selectfluor oxidative spirocyclization (Fig. 48).

Entry	Deviation from standard conditions	Yield / outcome
**1**	none (standard conditions)	90%
**2**	DCM, EtOAc	ineffective
**3**	DMF / DMA	38 / 30%
**4**	MeCN alone or other ratios	lower yield
**5**	NFSI	30%
**6**	DAST, Deoxofluor, CsF	0%
**7**	50 °C	58%
**8**	different Selectfluor loading	incomplete conversion / side reactions

Table S44. Optimization for Bag et al. - NIS ipso-iodocyclization/addition (Fig. 49).

Entry	Deviation from standard conditions	Yield / outcome
**1**	none (standard conditions)	94% (single diastereomer)
**2**	I_2 (1.1 equiv)	75%
**3**	*N*-iodosaccharin (1.1 equiv)	66%
**4**	other iodinating reagents	less effective / ineffective
**5**	1,3-diiodo-5,5-dimethylhydantoin (0.6 equiv)	excellent yield (94%)

Table S45. Optimization for Ueda et al. - cooperative acid/thiourea ipso-FC (Fig. 50).

Entry	Deviation from standard conditions	Yield / outcome
**1**	none (standard conditions)	94%
**2**	Rh catalyst in DCM	failed
**3**	Ag catalyst	poor yield
**4**	benzoic acid and variants	traces
**5**	more acidic catalysts	57–72%
**7**	maleic acid alone	79%
**6**	thiourea alone	inactive
**7**	alternative H-bonding catalysts / solvents	no improvement
**8**	chiral thiourea catalysts	near-racemic

Table S46. Optimization for Zhang et al. - IBX oxidative C2/C3 cyclization (Fig. 51).

Entry	Deviation from standard conditions	Yield / outcome
**1**	none (standard conditions)	96%, 10:1 *dr*
**2**	HFIP/H_2O 1:1	74%
**3**	HFIP/H_2O 5:1	79%
**4**	pure HFIP or pure water	reaction failed
**5**	TFE/H_2O, TFP/H_2O, OFP/H_2O	reduced yield, longer times
**6**	25 °C / 45 °C	77% / 79%

Table S47. Optimization for Yasui et al. - (thio)chloroformylation/spirocyclization (Fig. 52).

Entry	Deviation from standard conditions	Yield / outcome
**1**	none (standard conditions)	73% (triphosgene)
**2**	DCM	0%

**3**	DCE	20%
**4**	dioxane	45%
**5**	PhCl, DMA	0%
**4**	$AgBF_4$ (1 or 2 equiv)	31% / trace amounts
**5**	$AlCl_3$ or ZnI_2	53–56%
**6**	Et_3N (different amounts)	up to 78%
**7**	triphosgene (0.33 equiv)	71%

Table S48. Optimization for Dai et al. - sulfur ylide (2+1)/(1+1+1) (Fig. 53).

Entry	Deviation from standard conditions	Yield / outcome
**1**	none (standard conditions)	(2+1): sulfonium: 87%, 6:1 *dr*; Corey-Chaykovsky: 88%, >20:1 *dr* (1+1+1): 73%, 7:1 *dr*
**2**	Cs_2CO_3, K_3PO_4, NaOAc, $NaHCO_3$, Et_3N, DBU, TMG, DBACO	(2+1): lower yield
**3**	DCM, toluene, $CHCl_3$, THF, EtOH, EA	(2+1): lower yield
**4**	0 °C	(2+1): no improvement
**5**	$Mg(OTf)_2$, $Sc(OTf)_3$, $Al(OiPr)_3$	(1+1+1): lower yield
**6**	K_2CO_3, K_3PO_4, Et_3N, DBU, TMG, DBACO	(1+1+1): lower yield
**7**	MeCN, DCM, THF	(1+1+1): lower yield
**8**	0.5 mL toluene / 2 mL toluene	(1+1+1): 23% / 26%

Table S49. Optimization for Wang et al. - base-controlled hydrazonyl annulation (Fig. 54).

Entry	Deviation from standard conditions	Yield / outcome
**1**	none (standard conditions)	tetracycle: 73%<br>pentacycle: 64% / 29%
**2**	Et_3N	tetracycle: 38%<br>pentacycle: 23% / 12% (poor selectivity)
**3**	DBU / *t*BuOK / $NaHCO_3$	tetracycle: 24% / 11% / 15%<br>pentacycle: 0%
**4**	Cs_2CO_3 vs K_3PO_4	Cs_2CO_3 favors pentacycle; K_3PO_4 favors tetracycle
**5**	MeOH, dioxane, DMF	lower yield

Table S50. Optimization for Wang et al. - catalyst-free with quinone esters (Fig. 55).

Entry	Deviation from standard conditions	Yield / outcome
**1**	none (standard conditions)	90% (chromenoindole)
**2**	lower than 120 °C	79–86%
**3**	higher than 120 °C	83%
**4**	THF, MTBE, DME, MeCN, toluene, DCE	significantly lower
**5**	ethanol	trace

Table S51. Optimization for Zhang et al. - base-promoted formal [3+2] (Fig. 56).

Entry	Deviation from standard conditions	Yield / outcome
**1**	none (standard conditions)	95%, >19:1 *dr*
**2**	DCM / toluene / MeCN	53% / 67% / 25%
**3**	HFIP or H_2O	<5%
**4**	K_2CO_3 / DABCO / DBU / TEA	74% / 47% / 46% / 40%
**5**	$NaHCO_3$	<5%

Table S52. Optimization for Lv et al. - visible-light radical CF_3/cyclization (Fig. 57).

Entry	Deviation from standard conditions	Yield / outcome
**1**	none (standard conditions)	83%
**2**	*fac*-$Ir(ppy)_3$ / $[Ir(ppy)_2(dtbbpy)]PF_6$ / $[Ru(bby)_3]Cl_2$ / 4CzIPN / Eosin Y / Eosin B	22–64%
**4**	$NaHCO_3$ / Na_2CO_3 / $KHCO_3$ / DABCO	lower yields
**5**	DMSO / DCE / MeCN / THF / acetone	lower yields
**6**	no photocatalyst	no product

Table S53. Optimization for Moustakim et al. - UV-A continuous-flow photocyclization (Fig. 58).

Entry	Deviation from standard conditions	Yield / outcome
**1**	none (standard conditions)	82%
**2**	UV-A / UV-B / 450 nm LED	lower yields
**3**	batch	16–45%
**4**	dioxane / cyclohexane / PhH:MeOH / PhH:MeCN / dioxane:MeOH	16–38%

Table S54. Optimization for Ranga Rao et al. - visible-light/$K_2S_2O_8$ cascade (Fig. 59).

Entry	Deviation from standard conditions	Yield / outcome
**1**	none (standard conditions)	82%, single diastereomer
**2**	$Na_2S_2O_8$ / $PhI(OAC)_2$ / oxone / $(NH_4)_2S_2O_8$ / TBHP	lower yields
**3**	$K_2S_2O_8$ (2, 2.5, 3.5 equiv)	lower yields
**4**	DMF, DMSO, dioxane, CH_3CN	traces
**5**	2,2,2-trichlro ethane	65%
**6**	Photocatalyst	40–43%

Table S55. Optimization for Yang et al. - visible-light energy-transfer (T-ESPT) (Fig. 60).

Entry	Deviation from standard conditions	Yield / outcome
**1**	none (standard conditions)	85%
**2**	trifluoroethanol (TFE)	3%
**3**	DCM, MeOH, MeCN, DMF, DMSO	no product
**4**	other photocatalysts	ineffective
**5**	no light or no photocatalyst	no reaction
**6**	PC-I (0.5 / 1 / 4 mol%)	70% / 80% / 80%

Table S56. Optimization for Wang et al. - L-proline three-component condensation (Fig. 61).

Entry	Deviation from standard conditions	Yield / outcome
**1**	none (standard conditions)	91%
**2**	no catalyst	0% (24 h)
**3**	room temperature	trace
**4**	80 °C	82%
**5**	20 mol% L-proline	90%
**6**	*p*-TSA / AcOH / TsOH	45% / 40% / 46%
**7**	piperidine / Et_3N / DBU	28% / 20% / 31%
**8**	MeCN / DMF / dioxane / THF	62% / 66% / 60% / 55%
**9**	water / EtOH-H_2O	5% / 16%

Table S57. Optimization for Pan et al. - SPINOL-CPA asymmetric cascade (Fig. 62).

Entry	Deviation from standard conditions	Yield / outcome
**1**	none (standard conditions)	99%, 92:8 *er* (single *dr*)
**2**	other catalysts	less effective
**3**	DCE / PhMe / MeCN / CCl_4 / AcOEt / $CHCl_3$	no *ee* improvement / lower yield

**4**	room temperature	lower *ee*
**5**	-30 °C	longer time (72 h), lower yield and *ee*

Table S58. Optimization for Zhang et al. - BINOL-IDPA [3+2]/spirocyclization (Fig. 63).

Entry	Deviation from standard conditions	Yield / outcome
**1**	none (standard conditions)	92%, 99% *ee*, >19:1 *dr*
**2**	other catalysts	lower yield
**3**	toluene, DCE, CH_2Cl_2	lower yield
**4**	THF	0%
**5**	no additive / 3Å MS / 5Å MS	lower yield
**6**	48 h	no improvement
